# Mechanisms of nuclear pore complex disassembly by the mitotic Polo-like kinase 1 (PLK-1) in *C. elegans* embryos

**DOI:** 10.1126/sciadv.adf7826

**Published:** 2023-07-19

**Authors:** Sylvia Nkombo Nkoula, Griselda Velez-Aguilera, Batool Ossareh-Nazari, Lucie Van Hove, Cristina Ayuso, Véronique Legros, Guillaume Chevreux, Laura Thomas, Géraldine Seydoux, Peter Askjaer, Lionel Pintard

**Affiliations:** ^1^ Université Paris Cité, CNRS, Institut Jacques Monod, F-75013 Paris, France.; ^2^ Programme Équipe Labellisée Ligue contre le Cancer, Paris, France.; ^3^ Andalusian Center for Developmental Biology (CABD), CSIC/JA/Universidad Pablo de Olavide, Seville, Spain.; ^4^ HHMI and Department of Molecular Biology and Genetics, Johns Hopkins University School of Medicine, Baltimore, MD, USA.

## Abstract

The nuclear envelope, which protects and organizes the genome, is dismantled during mitosis. In the *Caenorhabditis elegans* zygote, nuclear envelope breakdown (NEBD) of the parental pronuclei is spatially and temporally regulated during mitosis to promote the unification of the maternal and paternal genomes. Nuclear pore complex (NPC) disassembly is a decisive step of NEBD, essential for nuclear permeabilization. By combining live imaging, biochemistry, and phosphoproteomics, we show that NPC disassembly is a stepwise process that involves Polo-like kinase 1 (PLK-1)–dependent and –independent steps. PLK-1 targets multiple NPC subcomplexes, including the cytoplasmic filaments, central channel, and inner ring. PLK-1 is recruited to and phosphorylates intrinsically disordered regions (IDRs) of several multivalent linker nucleoporins. Notably, although the phosphosites are not conserved between human and *C. elegans* nucleoporins, they are located in IDRs in both species. Our results suggest that targeting IDRs of multivalent linker nucleoporins is an evolutionarily conserved driver of NPC disassembly during mitosis.

## Teaser

PLK-1 targets intrinsically disordered regions of multiple multivalent nucleoporins to dismantle the nuclear pore complexes.

## INTRODUCTION

During each round of cell division in metazoans, cells undergo a profound reorganization: The replicated DNA condenses into individual chromosomes, and the nuclear envelope, which protects and organizes the genome, breaks down during mitosis (*1*, *2*). This step is critical for microtubules to access kinetochores and form the mitotic spindle. How nuclear envelope breakdown (NEBD) is regulated remains incompletely understood, particularly during development.

**Fig. 1.**
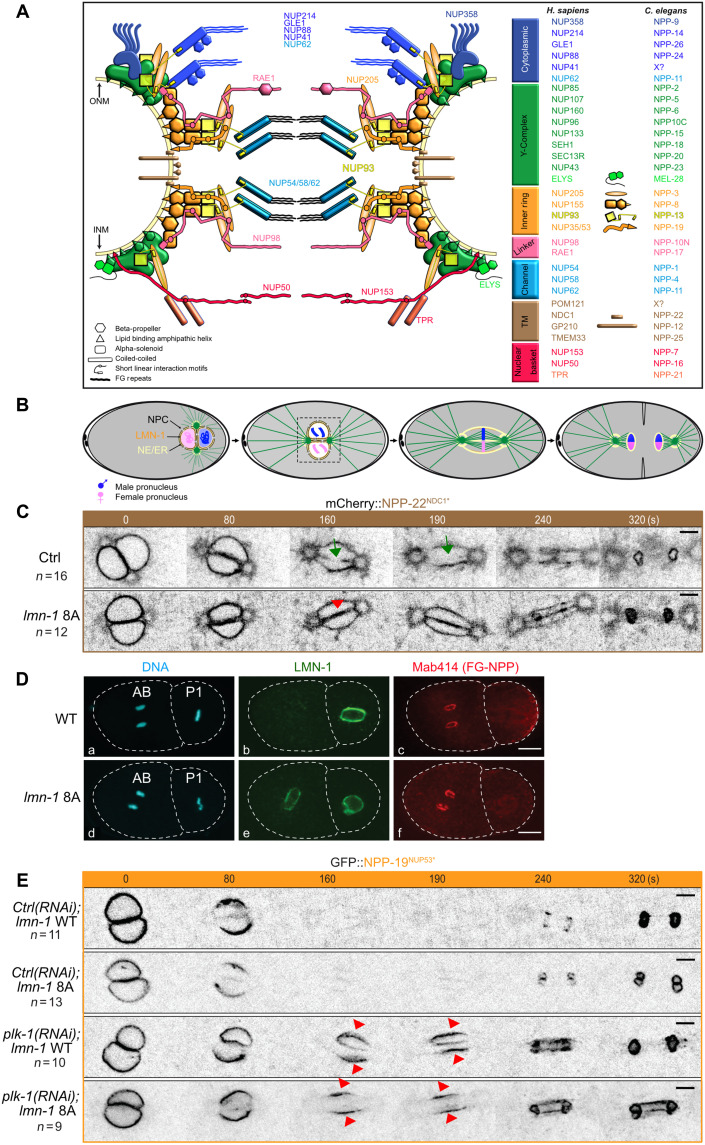
PLK-1 triggers nuclear pore complexes disassembly independently of lamina depolymerization. (**A**) Schematic of the nuclear pore complex and nomenclature of the NUPs in human cells and in *C. elegans* (NPP). (**B**) After meeting of the female (pink) and male (blue) pronuclei at the posterior pole, the nucleo-centrosomal complex undergoes a 90° rotation to align along the anteroposterior axis of the embryo. Parental chromosomes congress on the metaphase plate before the initiation of anaphase. In telophase, the NE reforms around decondensing chromosomes. During mitosis, remnant NE (yellow) remains around the segregating chromosomes. NPC, nuclear pore complex; NE/ER, nuclear envelope/endoplasmic reticulum. (**C**) Micrographs of wild-type (WT) or *lmn-1* 8A mutant embryos expressing GFP::NPP-22^NDC1^ in mitosis. The green arrow marks the presence of a membrane gap in control WT as opposed to *lmn-1* 8A embryos (red arrow). Timings in seconds are relative to pronuclei juxtaposition (0 s). The anterior is to the left in this and other figures. The asterisk (*) indicates the NUPs that are endogenously tagged in this and other figures. All panels are at the same magnification. Scale bars, 3 μm. *n* is the number of embryos analyzed in this and other figures. Data were collected from three independent experiments. (**D**) Confocal images of fixed WT and *lmn-1* 8A mutant two-cell embryos stained with LMN-1 (green) and Mab414 (red) antibodies and counterstained with 4′,6-diamidino-2-phenylindole (DAPI) (blue). All panels are at the same magnification. Scale bar, 10 μm. (**E**) Micrographs of live WT or *lmn-1* 8A embryos expressing GFP::NPP-19^NUP53^ in mitosis exposed to control, or *plk-1*(*RNAi*). The red arrowheads point to GFP::NPP-19^NUP53^ persisting on the NE during mitosis. Timings in seconds are relative to pronuclei juxtaposition (0 s). All panels are at the same magnification. Scale bars, 3 μm. Data were collected from three independent experiments.

The nuclear envelope is a double membrane composed of an outer and an inner phospholipid bilayer. Tethered to the inner nuclear membrane are the nuclear lamina and lamina-associated proteins which contribute to the mechanical integrity of the nucleus and organization of the genome during interphase. Transport across the two membranes occurs exclusively through nuclear pore complexes (NPCs), composed of roughly 30 evolutionarily conserved nucleoporins (NUPs) (Fig. 1A), present in 8 to 32 copies (*3*, *4*). Within the nuclear pores, NUPs assemble into modular subcomplexes organized as a three-ring stacked structure: one inner ring (in orange and yellow in Fig. 1A) and two outer rings, containing the Y-complexes (green), located on the cytoplasmic and nuclear faces of the nuclear pores (*3*–*5*). The inner ring and the Y-complexes, which are anchored to the membrane by interacting with transmembrane NUPs (brown) and the lipid bilayer, serve as building blocks for the scaffolding of nuclear pores (Fig. 1A). Attached to these rings are NUPs of the central channel (light blue), cytoplasmic filaments (dark blue), and nuclear basket (red) responsible for the translocation of substrates through the NPCs (Fig. 1A). NUPs with phenylalanine-glycine (FG) repeats line the central channel and serve a dual role: They form the permeability barrier by restricting the passive diffusion of macromolecules larger than ∼40 kDa, and they provide binding sites for nuclear transport receptors (*6*, *7*).

Disassembly of the NPC is a decisive step of NEBD required for nuclear permeabilization. In vertebrate cells, several mitotic kinases, including Cyclin B–Cdk1, the Polo-like kinase Plk1, Nek6/7, and possibly others, phosphorylate multiple NUPs to dismantle the NPC and coordinate NPC disassembly with cell cycle progression (*8*). In particular, the multisite phosphorylation of the FG-NUP NUP98, the “gatekeeper” of the NPC, promotes nuclear permeabilization (*8*–*10*). The dispersal of the soluble NUPs then rapidly and synchronously follows NUP98 removal (*11*). Phosphorylation of NUP53, a linker NUP of the inner ring, is critical in this process. NUP53 directly interacts with NUP205, NUP155, NUP93, the transmembrane NUP NDC1, and the lipid bilayer (Fig. 1A). Multisite phosphorylation of NUP53 by Cyclin-Cdk and Plk1 disrupts these interactions and promotes the disassembly of the central NPC (*8*, *10*). It also triggers the dissociation of the central channel subcomplex composed of the FG NUPss NUP54, NUP58, and NUP62 connected to the NPC by NUP93 (Fig. 1) (*12*, *13*). The mechanisms responsible for the disassembly of the other NPC subcomplexes are less well understood (*8*). Likewise, how other metazoans dismantle their NPCs has not been systematically investigated. Whether NUPs depart the NPCs in a similar order and via similar mechanisms is currently unclear.

The one-cell *Caenorhabditis elegans* embryo provides a dynamic developmental context to dissect the molecular mechanisms regulating NPC disassembly and NEBD (*14*, *15*). Nuclear envelope components are conserved in *C. elegans*, including the lamina, encoded by a single gene *lmn-1* (B-type lamin) (*16*) and most of the NUPs, although the sequence identity between human and *C. elegans* NUPs is not higher than 25% (Fig. 1A) (*15*, *17*).

In the one-cell embryo just after fertilization, parental chromosomes are initially located in separate pronuclei, each surrounded by a nuclear envelope (Fig. 1B). Coordinated disassembly of the pronuclear envelopes is required for the unification of the parental genomes after the first mitosis. In contrast to vertebrate cells, where NEBD occurs in prophase, NEBD is regulated both spatially and temporally in the semi-open mitosis of the *C. elegans* embryo (Fig. 1B) (*14*, *15*, *18*–*20*). The first reported sign of NEBD in the zygote is the deformation of the nuclear envelope in the vicinity of centrosomes, and its permeabilization, detectable by the presence of soluble tubulin in the pronuclear space (*21*). NPC disassembly starts in the vicinity of the centrosomes and progresses to the juxtaposed pronuclear envelopes located between the parental chromosomes (*18*, *19*, *22*, *23*). However, some NUPs and the lamina persist on the remnant envelopes surrounding the mitotic spindle until the metaphase-to-anaphase transition (*18*–*20*, *22*, *23*). After lamina depolymerization is completed, the proper mingling of both parental chromosome sets on the metaphase plate requires the formation of a membrane scission event (also called a membrane gap) (Fig. 1B) (*24*).

Mitotic kinases, including Cyclin B–Cdk1 (*25*) Aurora A (AIR-1) (*19*, *26*), and the Polo-like kinase 1 (PLK-1), have been implicated in NEBD in *C. elegans* (*20*, *22*, *27*, *28*). In particular, PLK-1 plays a prominent role in NEBD in the early embryo. Consistently, PLK-1 is dynamically recruited to the nuclear envelope in prophase just before NEBD (*20*) via its C-terminal phosphopeptide binding domain (*29*, *30*), the Polo-box domain (PBD) (*20*). While in vertebrate cells the Plk1 PBD only binds NUP53 on the inner ring complex (*10*), in *C. elegans*, the PBD binds NUPs of the central channel NPP-1^NUP54^, NPP-4^NUP58^, and NPP-11^NUP62^ primed by Cyclin B–Cdk1 and PLK-1 phosphorylation (*20*). Once recruited to the central channel NUPs, PLK-1 most likely contributes to dismantling the NPC, but its phosphorylation targets, with a structural role in the NPC, are currently unknown. Confirming genome-wide protein interaction maps using yeast two-hybrid assays (*31*), we previously showed that the PLK-1 PBD also binds a *C. elegans* NPP-19^NUP53^ (1 to 301 amino acids) fragment primed by phosphorylation (*20*), but the functional importance of this interaction remains to be investigated.

Beyond NPC disassembly, PLK-1 triggers lamina depolymerization in *C. elegans* by directly phosphorylating the lamina (*22*). In fact, expression of a *lmn-1* 8A allele, mutated on eight PLK-1 phosphorylation sites (*32*), is sufficient to prevent lamina depolymerization, membrane gap formation, and the fusion of the parental genomes during mitosis (*21*, *22*). Whether stabilization of the lamina affects the disintegration of the NPC is not known. Some NUPs and the lamina accumulate at the NE upon *plk-1* inactivation (*28*) suggesting that PLK-1 might regulate both lamina depolymerization and NPC disassembly. Alternatively, as the lamina interacts physically with NUPs (*33*–*35*), lamina persistence could alter NPC disassembly. The *lmn-1* 8A allele represents a unique genetic tool to test these possibilities.

Here, by combining live imaging of fluorescently tagged NUPs with biochemical and phosphoproteomic approaches, we characterize the process and mechanism of NPC disassembly in the one-cell *C. elegans* embryo. We show that NPC disassembly occurs in two major steps, PLK–independent and PLK-1–dependent. During the first step, the nuclear basket and the Y-complex subunits leave the NPC early in prophase but without affecting the nuclear permeability barrier. During the second step, PLK-1 is recruited to NUPs of the central channel, the inner ring, and phosphorylates intrinsically disordered regions (IDRs) of multivalent linker NUPs leading to nuclear permeabilization and complete NPC disassembly, independently of lamina depolymerization. This work provides a comprehensive analysis of the NPC disassembly mechanism in a living organism.

## RESULTS

### PLK-1 triggers the disassembly of soluble nucleoporins independently of lamina depolymerization

To investigate whether lamina stabilization affects NPC disassembly, we analyzed the dynamics of the transmembrane NUP NPP-22^NDC1^, endogenously tagged with mCherry, in wild-type (WT) versus *lmn-1*8A mutant one-cell embryos during mitosis using spinning disc confocal microscopy (Fig. 1, B and C). mCherry::NPP-22^NDC1^ localized to the nuclear envelope of the male and female pronuclei during their migration in WT embryos (Fig. 1C) (*21*, *36*). Then, as the embryos progressed through mitosis, it was progressively excluded from the region between the juxtaposed pronuclei, starting where the characteristic pronuclear membrane scission (gap) event occurs (Fig. 1C, green arrows) (*24*) and localized to the endoplasmic reticulum surrounding the centrosomes, recently named the “centriculum” (*37*). By contrast, in *lmn-1* 8A mutant embryos, where the lamina is not dismantled during mitosis, mCherry::NPP-22^NDC1^ also persisted on the juxtaposed pronuclear envelopes (Fig. 1C, red arrowhead). Thus, stabilization of the lamina can influence the dynamics of NPP-22^NDC1^ during mitosis.

Next, we monitored NUPs containing FG repeats using the Mab414 antibodies (*38*) in indirect immunofluorescence experiments. The asynchronous mitoses of the two-cell stage embryo (Fig. 1D, a and d) allow a direct comparison of nuclear envelope component behavior in metaphase (the P1 cell, right) and telophase (the AB cell, left). In WT embryos, FG NUPs were undetectable in metaphase (P1 blastomere) (Fig. 1Dc), while the lamina still localized around the DNA (Fig. 1Db) indicating that NUPs disassemble before lamina depolymerization. During nuclear envelope reformation, the FG NUP signal reaccumulated around the decondensing DNA in telophase in the AB blastomere (Fig. 1Dc), while the lamina had not yet polymerized (Fig. 1Db). In *lmn-1* 8A embryos, where lamina persists during mitosis (Fig. 1De), FG NUPs presented a similar localization as in WT embryos (Fig. 1Df), indicating that the persistence of the lamina in the nuclear envelope has no major impact on their disassembly or reassembly during mitosis.

To extend these observations, we filmed one-cell embryos expressing endogenously tagged GFP::NPP-19^NUP53^, a core subunit of the inner ring complex (*35*, *39*–*44*). GFP::NPP-19^NUP53^ disappeared from the nuclear envelope with the same kinetics in WT and *lmn-1* 8A mitotic embryos confirming that the persistence of the lamina does not affect the disassembly of soluble NUPs (Fig. 1E). However, GFP::NPP-19^NUP53^ readily persisted on the nuclear envelope throughout mitosis upon partial *plk-1* inactivation, both in WT and in *lmn-1* 8A embryos (Fig. 1E, red arrowheads).

Together, these observations indicate that PLK-1 promotes the disassembly of some NUPs independently of lamina depolymerization, although forcing maintenance of the lamina is sufficient to alter, directly or indirectly, the dynamics of the transmembrane NUP NPP-22^NDC1^ (Fig. 1C).

### PLK-1 targets the cytoplasmic filaments, the inner ring complex, and the central channel nucleoporins

To identify the nuclear pore subcomplexes targeted by PLK-1, we used spinning disc confocal microscopy to monitor the dynamics of fluorescently tagged NUPs from each NPC subcomplex during mitosis in control embryos, or upon partial *plk-1* inactivation. Since PLK-1 regulates several processes in the early embryo (*20*, *28*, *45*–*51*), we used partial RNA interference (RNAi) to exclude potential indirect effects.

We filmed embryos expressing fluorescently tagged NUPs of the cytoplasmic filaments (GFP::NPP-9^NUP358^, GFP::NPP-24^NUP88^), the Y-complex (GFP::NPP-5^NUP107^, GFP::MEL-28^ELYS^), the inner ring [superfolder (s)GFP::NPP-13^NUP93^, mCherry::NPP-8^NUP155^], the central channel (NPP-1^NUP54^::GFP and GFP::NPP-11^NUP62^), and the nuclear basket (sGFP::NPP-7^NUP153^ and NPP-21^TPR^::GFP). We also monitored the dynamics of NPP-10N^NUP98^ (tagged with NeonGreen), the *C. elegans* counterpart of NUP98, which belongs to multiple subcomplexes in human cells (*4*, *52*), and possibly also in *C. elegans*. Some NUPs were endogenously tagged with fluorescent markers using CRISPR-Cas9 (marked by an asterisk on the figures), whereas others were expressed from single-copy transgenes (see Material and Methods).

**Fig. 2.**
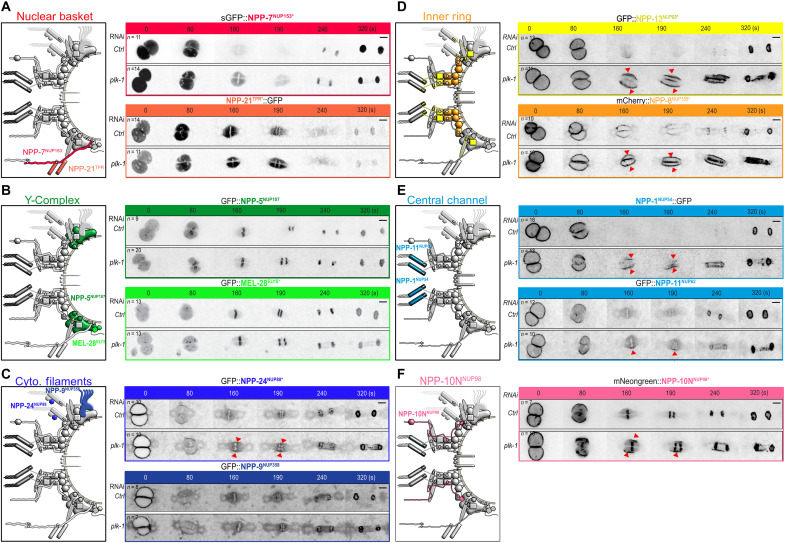
Nucleoporins of the cytoplasmic filaments, the central channel, and the inner ring accumulate at the nuclear envelope upon *plk-1* inactivation. (**A** to **F**) Micrographs of embryos expressing the indicated tagged NUPs exposed to control or *plk-1(RNAi)* starting when the pronuclei are juxtaposed, centered, and aligned along the anteroposterior axis of the embryo (time 0). The red arrowheads show the persisting NUPs on the NE during mitosis. All panels are at the same magnification. Scale bar, 3 μm. Data were collected from three independent experiments.

At time 0, defined as when the two pronuclei are juxtaposed and centered in the middle of the embryos, all the NUPs we monitored localized to the nuclear envelope (Fig. 2, C to F), except NUPs of the nuclear basket and of the Y-complex (Fig. 2, A and B). The basket NUPs GFP::NPP-7^NUP153^ and GFP::NPP-21^TPR^ had relocalized to the nucleoplasm at this time point (Fig. 2A and fig. S1, A and B), while the Y-complex subunits GFP::NPP-5^NUP107^ and GFP::MEL-28^ELYS^ had begun to accumulate at the kinetochores (Fig. 2B). Partial *plk-1* inactivation did not noticeably affect the dynamics of these NUPs during mitosis (Fig. 2, A and B). Both GFP::NPP-5^NUP107^ and GFP::MEL-28^ELYS^ localized to the kinetochores as in control embryos (Fig. 2B).

On the cytoplasmic face of the NPC, NUP88 forms a complex with NUP214 (*52*). As shown in Fig. 2C, *plk-1* inactivation affected the dynamics of GFP::NPP-24^NUP88^. While this NUP relocalized to the centriculum and the mitotic spindle area during mitosis in control and *plk-1(RNAi)* embryos, a fraction persisted on the remnant envelope surrounding the mitotic spindle during the metaphase-to-anaphase transition upon reduction of *plk-1* function (Fig. 2C). NPP-14^NUP214^::OLLAS similarly persisted on the nuclear envelope remnants upon partial inactivation of *plk-1* during mitosis (fig. S1C), indicating that PLK-1 controls the disassembly of the cytoplasmic filaments. GFP::NPP-9^NUP358^, which also localizes to the cytoplasmic face of the NPC, was however unaffected upon *plk-1* inactivation (Fig. 2C).

Next, we monitored the dynamics of the NUPs of the inner ring complex. Within this complex, in other systems, the linker nucleoporin NUP53 has a scaffolding role and binds NUP155, NUP93, and NUP205 (*35*, *40*–*44*). Previous immunofluorescence studies in early *C. elegans* embryos showed that NPP-3^NUP205^ and NPP-13^NUP93^ localize to the nuclear envelope before NEBD but then disappear first in the vicinity of the centrosomes (*19*). We confirmed these observations using endogenously tagged GFP::NPP-13^NUP93^. Before NEBD, when the two pronuclei are juxtaposed (time 0), GFP::NPP-13^NUP93^ localized to the nuclear envelope but after 160 s, it was barely detectable at the nuclear envelope (Fig. 2D). However, upon *plk-1* partial inactivation, GFP::NPP-13^NUP93^ persisted on the nuclear envelope throughout mitosis (Fig. 2D, red arrowheads), similar to GFP::NPP-19^NUP53^ (Fig. 1E). mCherry::NPP-8^NUP155^ also persisted on the nuclear envelope throughout mitosis upon *plk-1* inactivation and failed to relocalize to the centriculum (Fig. 2D). Thus, three components of the inner ring complex: NPP-8^NUP155^, NPP-13^NUP93^, and NPP-19^NUP53^ persist on the nuclear envelope upon partial *plk-1* inactivation.

Within the inner ring complex, NPP-13^NUP93^ recruits the FG NUPs NPP-1^NUP54^, NPP-4^NUP58^, and NPP-11^NUP62^, which form a trimeric complex located in the central channel of the NPC (Fig. 1A) (*12*, *20*). In control conditions, NPP-1^NUP54^::GFP localized to the nuclear envelope and was still detected on the remnant envelopes 80 s after juxtaposition of the pronuclei. As the embryo progressed through mitosis, it became undetectable and reaccumulated on the nuclear envelope only in telophase (Fig. 2E). However, NPP-1^NUP54^::GFP persisted on the nuclear envelope during mitosis upon partial depletion of PLK-1, consistent with previous observations (*28*). GFP::NPP-11^NUP62^ also persisted in the nuclear envelope upon *plk-1* inactivation (Fig. 2E). Thus, *plk-1* depletion also affects the disassembly of the central channel NUPs from the NPCs.

Last, we analyzed the dynamics of NeonGreen::NPP-10N^NUP98^, whose vertebrate homolog Nup98 is the gatekeeper of the NPC that links the inner ring to the Y-complex (*6*). In WT conditions, NeonGreen::NPP-10N^NUP98^ localized to the NE but relocalized to the kinetochores during mitosis. Upon PLK-1 depletion, NeonGreen::NPP-10N^NUP98^ also relocalized to the kinetochores but a fraction persisted on the nuclear envelope during mitosis (Fig. 2F, red arrows).

Together, these observations indicate that PLK-1 inactivation affects the disassembly of the cytoplasmic filaments (NPP-14^NUP214^ and NPP-24^NUP88^), the inner ring complex (NPP-8^NUP155^, NPP-13^NUP93^, NPP-19^NUP53^, and NPP-10N^NUP98^), and the central channel (NPP-1^NUP54^ and NPP-11^NUP62^) during mitosis.

### PLK-1 recruitment to the nuclear envelope precedes the disassembly of filament, central channel, and inner ring subcomplexes

The order and precise timing of NPC subcomplex disassembly have not yet been characterized in *C. elegans*. In human tissue culture cells, NUP98 is the first NUP to leave the NPC during mitosis, causing nuclear permeabilization (*9*, *11*). Our observations suggest that NUPs of the Y-complex and the nuclear basket leave the NPC well before NPP-10N^NUP98^, already during pronuclear migration and meeting (Fig. 2), possibly even before PLK-1::sGFP is recruited to the NE and the nuclear permeability barrier is ruptured.

To precisely characterize the order of NPC disassembly relative to PLK-1 recruitment to the NE, we used live-spinning disc confocal microscopy at high temporal resolution (one image/2 s). We quantified the levels of NUPs of the different subcomplexes and PLK-1::sGFP at the NE, relative to the rupture of the nuclear permeability barrier and anaphase onset (time 0). In this experiment, we used anaphase onset (which typically occurs 190 s after centration of the juxtaposed pronuclei) as time 0 to rigorously compare the timing of NUP departure from the nuclear envelope in different strains and embryos.

To determine when the NE becomes permeable, we quantified the amount of free mCherry::Histone, which is higher in the nucleoplasm than in the cytoplasm before NEBD but equilibrates between the two compartments once the NE becomes permeable (see Materials and Methods).

**Fig. 3.**
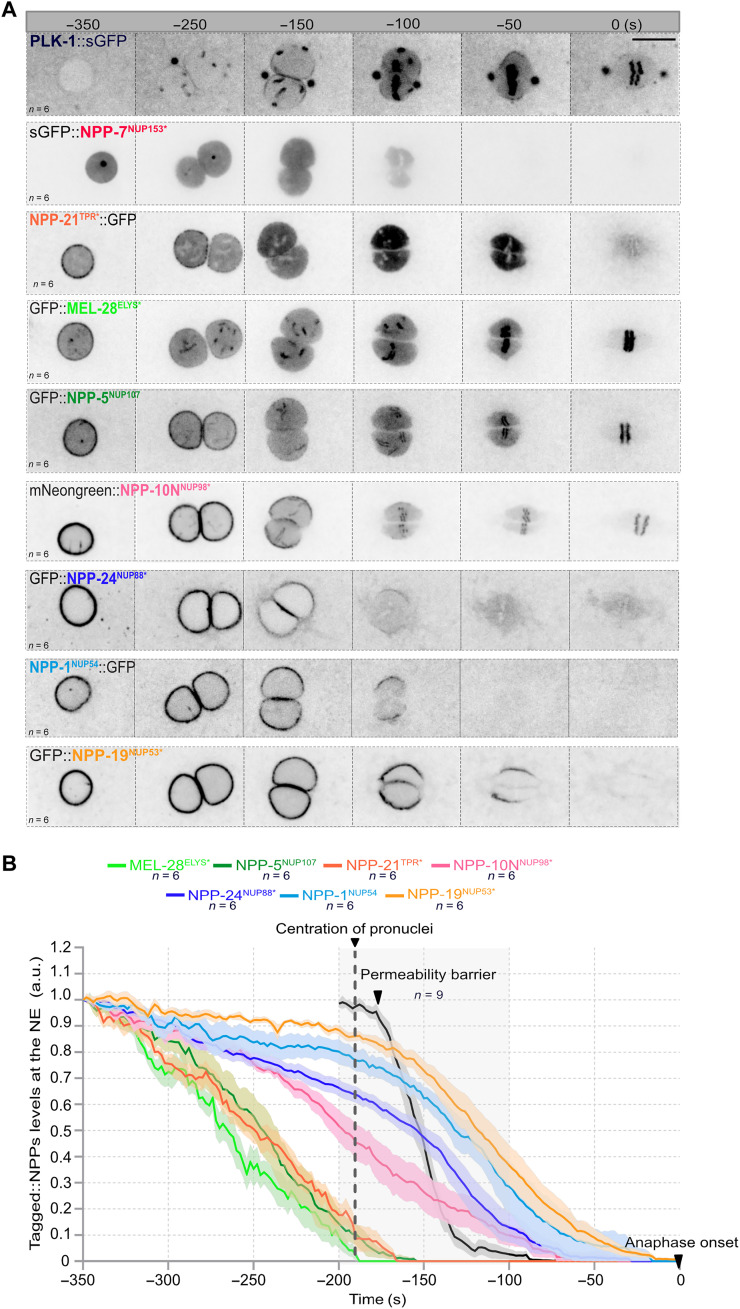
Timing of NPC subcomplexes disassembly in the *C. elegans* zygote relative to the rupture of the nuclear permeability barrier. (**A**) Micrographs of one-cell embryos expressing PLK-1::sGFP or the indicated tagged NUPs from pronuclear migration and meeting to anaphase onset (time 0). All panels are at the same magnification. Scale bar, 10 μm. (**B**) Graph presenting the quantification of GFP::NPP signal intensity at the NE in the *C. elegans* zygote from pronuclear nuclear migration to anaphase onset (time 0). The average signal intensity of the fluorescently tagged NPPs at the NE 350 s before anaphase was arbitrarily defined as 1. The means ± SEM is presented for *n* = 6 embryos. Data were collected from three independent experiments. The dark line presents the quantification of the nuclear permeability barrier starting 200 s before anaphase onset. a.u., arbitrary units.

This analysis confirmed that NUPs of the basket and the Y-complex leave the nuclear envelope well before it becomes permeable (Fig. 3A). GFP::NPP-7^NUP153^ localized to the NE during pronuclear migration (−410 s before anaphase; fig. S2A, arrow) but had already relocalized to the nucleoplasm before the pronuclear meeting. Likewise, NPP-21^TPR^::GFP had almost entirely relocalized to the nucleoplasm at the time of the pronuclear meeting (Fig. 3B and fig. S2B). The Y-complex subunit GFP::MEL-28^ELYS^ similarly disappeared from the nuclear envelope by pronuclei meeting and relocalized to the kinetochores, concomitantly with chromosome condensation (Fig. 3B and fig. S2C). The other Y-complex subunits GFP::NPP-5^NUP107^, GFP::NPP-6^NUP160^, and GFP::NPP-18^SEH1^ departed the NE slightly after GFP::MEL-28^ELYS^ but all behaved similarly (Fig. 3B and fig. S2, D to F), suggesting that the entire Y-complex leaves the NE early in prophase to relocalize to the kinetochores. Quantifications of the signal at the pronuclear envelope revealed that GFP::NPP-7^NUP153^ is the first NUP to leave the NE followed by GFP::MEL-28^ELYS^, NPP-21^TPR^::GFP, and the other Y-complex subunits (Fig. 3B and fig. S2G). All these subunits leave the NE before the recruitment of PLK-1::sGFP, which starts to accumulate at the NE 250 s before anaphase onset (Fig. 3A). PLK-1::sGFP accumulation at the NE precedes the departure of NPP-10N^NUP98^, NPP-24^NUP54^, NPP-1^NUP54^, and NPP-19^NUP53^. As shown Fig. 3, NPP-10N^NUP98^ leaves the NE slightly before the cytoplasmic filament NUP NPP-24^NUP88^ and the central channel NUP NPP-1^NUP54^, which disappears from the NE before NPP-19^NUP53^ (Fig. 3 and fig. S3). The NE becomes permeable only when NPP-10N^NUP98^ is removed from the NPC (Fig. 3A). More specifically, we observed a loss of permeability barrier when almost 60% of NPP-10N was removed from the NE (Fig. 3B).

Together, these data indicate that the Y-complex and the nuclear basket NUPs leave the NE before PLK-1 recruitment, which precedes the departure of NPP-10N^NUP98^ and the rupture of the nuclear permeability barrier (Fig. 3).

### A biochemical screen for PLK-1 PBD–interacting phospho-nucleoporins

We next searched for the direct PLK-1 substrates at the NPCs. PLK-1 uses its conserved C-terminal PBD to bind its substrates phosphorylated on specific sequence motifs [aka Polo-docking sites (S-pS/pT-P/X)] that are created by other priming kinases (non–self-priming) or are self-primed by PLK-1 itself. This provides an efficient mechanism to regulate PLK-1 subcellular localization and substrate selectivity in space and time (*29*, *30*, *53*–*55*). We previously showed that PLK-1 uses its PBD domain to localize to the nuclear envelope in the early *C. elegans* embryo where it binds NUPs of the central channel NPP-1^NUP54^, NPP-4^NUP58^, and NPP-11^NUP62^ phosphorylated on Polo-docking sites by PLK-1 and cyclin B–Cdk1 (*20*).

**Fig. 4.**
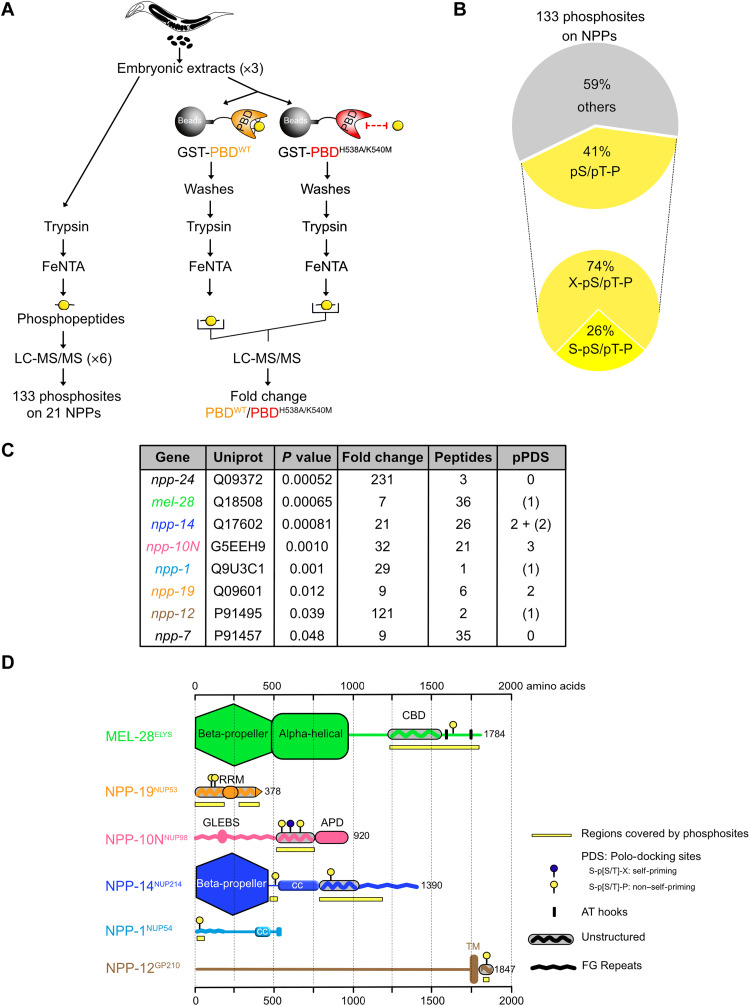
A biochemical and phosphoproteomic screen for PLK-1 targets at the nuclear pore complexes. (**A**) Flowchart of the approach used to map the NUPs phosphoproteome and identify NUPs specifically binding the Plk1 PBD. Embryonic extracts prepared from young adults were digested with trypsin and phosphopeptides were affinity-purified using FeNTA immobilized metal ion affinity chromatography columns before identification by tandem mass spectrometry (MS/MS). A fraction of the embryonic extracts were incubated on an affinity matrix consisting of the glutathione *S*-transferase (GST)–PBD WT or mutated on the phosphopincers. After several washing steps, retained proteins were digested with trypsin and phosphopeptides were affinity-purified on FeNTA before identification by liquid chromatography–MS/MS (LC-MS/MS). (**B**) Circle chart showing the proportion of phosphorylation sites identified on NUPs presenting the consensus for phosphorylation by proline-directed kinases [(pS/pT-P), Cyclin-Cdk]. The circle chart below presents the percentage of these phosphosites matching the consensus for non–self-priming phosphorylation (S-pS/pT-P). (**C**) Table summarizing the label-free quantitative MS analysis of the GST-PBD WT or mutated on the phosphopincers pull-downs from three independent *C. elegans* embryonic extracts. The *P* value (<0.05) indicates statistical significance for the enrichment of the NUPs in the GST-PBD pull-down. pPDS (phosphorylated Polo-docking sites) indicate the number of Polo-docking sites identified in the different NUPs. The value in parentheses corresponds to cases where a phosphorylated peptide contains a Polo-docking site but the exact position of the phosphorylation is ambiguous. (**D**) Domain organization and distribution of the phosphorylation sites on the NUPs specifically retained on the GST-PLK-1 PBD affinity matrix: MEL-28^ELYS^, NPP-19^NUP53^, NPP-10N^NUP98^, NPP-14^NUP214^, NPP-1^NUP54^, and NPP-12^gp210^. CBD, chromatin-binding domain; RRM, RNA recognition motif; APD, auto-proteolytic domain; CC, coiled-coiled.

To determine which NUPs are phosphorylated on Polo-docking sites in vivo, we analyzed the *C. elegans* embryos phosphoproteome using FeNTA affinity chromatography and liquid chromatography–tandem mass spectrometry (LC-MS/MS) (Fig. 4A). While previous phosphoproteomics studies identified a total of 62 sites on NUPs (PHOSIDA) (*56*), we mapped 133 phosphosites on 21 of the 28 NUPs characterized in worms (Fig. 4B, table S1, and data S1). Forty-one percent (54 of 133) of these sites match the minimal consensus for Cyclin-Cdk–dependent phosphorylation (pS/pT-P), among which 26% (14 of 54) are part of Polo-docking sites suggesting that Cyclin-Cdk primes PLK-1 binding to multiple NUPs (Fig. 4B). We identified 14 phosphorylated Polo-docking sites matching the consensus for non–self-priming (S-pS/pT-P) in the NUPs of the cytoplasmic filaments NPP-14^NUP214^ (five Polo-docking sites), the central channel NPP-1^NUP54^ (one Polo-docking site), in the transmembrane NUPs NPP-12^gp210^ (one Polo-docking site) and NPP-22^NDC1^ (one Polo-docking site), in the subunits of the inner ring complex NPP-8^NUP155^ (one Polo-docking site) and NPP-19^NUP53^ (one Polo-docking site), but also in NPP-10N^NUP98^ (two Polo-docking sites), NPP-16^NUP50^ (one Polo-docking site), and MEL-28^ELYS^ (one Polo-docking site) (table S1 and data S1). In addition, we identified one phosphorylated Polo-docking site matching the consensus for self-priming (S-pS/pT-X) in NPP-10N^NUP98^ (table S1 and data S1).

To identify bona fide Plk1 PBD-interacting phospho-NUPs, we combined affinity purification with phosphoproteomics (Fig. 4A). We loaded embryonic extracts on an affinity matrix containing either the glutathione *S*-transferase (GST)–Plk1 PBD WT or a control GST-Plk1 PBD^H538A/K540M^, carrying mutations on the “phosphopincers” (*29*, *30*, *53*), and thus defective in phosphopeptides binding (Fig. 4A). The retained proteins were digested with trypsin, and phosphopeptides were purified on FeNTA affinity chromatography (FeNTA) before identification by LC-MS/MS. Here, we specifically concentrated our analysis on NUPs; full details of this analysis will be published elsewhere.

Through this approach, we recovered phosphopeptides corresponding to NPP-1^NUP54^, MEL-28^ELYS^, NPP-10N^NUP98^, NPP-14^NUP214^, NPP-19^NUP53^, NPP-7^NUP153^, NPP-12^gp210^, and NPP-24^NUP88^ (*P* < 0.05, *n* = 3 independent experiments using different embryo extracts; fig. S4 and table S2), but we unambiguously detected phosphorylated Polo-docking sites only in NPP-10N^NUP98^ (three sites), NPP-14^NUP214^ (two sites), and NPP-19^NUP53^ (two sites) (Fig. 4C, table S3, and data S2). Several MEL-28^ELYS^ phosphopeptides were recovered, including one encompassing a Polo-docking site, but the exact position of the phosphorylation could not be unambiguously assigned on this peptide. We note that this specific Polo-docking site was already detected phosphorylated in embryo extracts (table S1 and data S1). Likewise, we recovered in the PBD pull-downs phosphopeptides covering one Polo-docking site in NPP-1^NUP54^ and NPP-12^gp210^. As for MEL-28^ELYS^, both Polo-docking sites were unambiguously phosphorylated in the embryonic extracts strongly suggesting that MEL-28^ELYS^, NPP-1^NUP54^, and NPP-12^gp210^ were specifically recovered in the PLK-1 PBD pull-downs via these phosphorylated Polo-docking sites.

Next, we analyzed the distribution of the phosphorylation sites on the various functional domains of these NUPs; MEL-28^ELYS^, the first NUP to associate with chromatin at the end of mitosis (*57*, *58*), is a large protein composed of multiple functional domains: an N-terminal β-propeller domain, a central α helical domain, and an unstructured C-terminal region that includes two AT hooks (Fig. 4D). The N-terminal domains are required for NPC targeting and kinetochore localization, whereas the C-terminal domain is required for chromatin binding (*59*). Most of the phosphorylation sites identified on MEL-28^ELYS^, including the phosphorylated Polo-docking site, localized on the chromatin-binding region (Fig. 4D), suggesting that phosphorylation could modulate this activity.

Phosphorylation sites were also restricted to a defined region in NPP-10N^NUP98^, which contains Glycine-Leucine-Phenylalanine-Glycine (GLFG) amino acids repeats and a Rae1 binding site in the N-terminal region, similar to its human counterpart NUP98 (Fig. 4D). The C-terminal part, located upstream of the conserved autoproteolytic domain, is however poorly conserved (fig. S5A) (*44*). This region is predicted to be unstructured in both human NUP98 and *C. elegans* NPP-10N^NUP98^. Most of the phosphorylation sites localized to this IDR of NPP-10N^NUP98^ are embedded in motifs matching the consensus for PLK-1 phosphorylation or PLK-1 docking (Fig. 4D and fig. S5A).

Likewise, NPP-14^NUP214^, besides containing an evolutionarily conserved β-propeller domain, is mainly unstructured with FG repeats in the C-terminal part of the protein. Here again, most of the phosphosites localized to the nonconserved and unstructured region (Fig. 4D). Phosphorylated Polo-docking sites were also identified in the unstructured regions of NPP-19^NUP53^, which contains an RNA recognition motif dimerization domain and a C-terminal alpha helix required for membrane binding (*60*). Last, the unique phosphorylated Polo-docking sites (SpTP) found in NPP-1^NUP54^ and NPP-12^gp210^ also localized in the unstructured N-terminal FG repeat of NPP-1^NUP54^ and C-terminal cytoplasmic tail of the integral transmembrane protein NPP-12^gp210^ (Fig. 4D).

In summary, most of the identified phosphorylation sites are distributed in unstructured, IDRs of the NUPs, specifically interacting with the PLK-1 PBD. The identification of NPP-1^NUP54^, NPP-14^NUP214^, NPP-19^NUP53^, and NPP-10N^NUP98^ as specific phospho-binding partners of the Plk1 PBD is consistent with our findings showing that PLK-1 depletion affects the disassembly of these NUPs, and their direct binding partners, during mitosis (Fig. 2).

### NPP-10N^NUP98^ contributes to PLK-1 recruitment to the nuclear envelope, possibly through direct phospho-dependent binding to the PBD

**Fig. 5.**
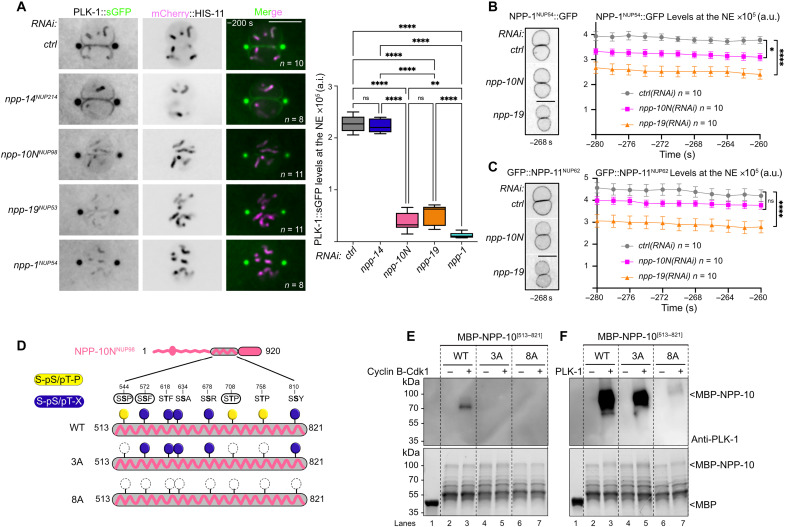
Phosphorylated NPP-10N^NUP98^ binds the PLK-1 PBD and contributes to PLK-1 recruitment to the nuclear envelope. (**A**) Micrographs of an embryo expressing PLK-1::sGFP (shown alone and in green in the merged images) and mCherry::HIS-11 (magenta, in the merged image) exposed to control (*ctrl*), *npp-14^NUP214^*, *npp-10N^NUP98^*, *npp-19^NUP53^*, or *npp-1^NUP54^* RNAi. All panels are at the same magnification. Scale bar, 10 μm. The graph on the right presents the quantification of PLK-1::sGFP at the NE in the different RNAi conditions 200 s before anaphase onset. **P < 0.01 and ****P < 0.0001; ns, not significant. Data were collected from three independent experiments. (**B** and **C**) Micrographs of embryos expressing NPP-1^NUP54^::GFP (B) or GFP::NPP-11^NUP62^ (C) exposed to control (*ctrl*), *npp-10N^NUP98^*, or *npp-19^NUP53^* RNAi. The graphs on the right present the quantification of NPP-1^NUP54^::GFP or GFP::NPP-11^NUP62^ average intensity at the NE at six time points relative to anaphase onset (timing in seconds is relative to anaphase onset). *P < 0.05 and ****P < 0.0001. Data were collected from three independent experiments. (**D**) Schematic and domain organization of NPP-10N^NUP98^. The C-terminal disordered NPP-10N^NUP98^ (513 to 821) fragment contains three Polo-docking sites matching the consensus for non–self-priming (yellow, phosphorylatable by Cyclin-Cdk1) and five sites matching the consensus for self-priming (blue, phosphorylatable by PLK-1). The sites that are circled have been identified in vivo. Besides the WT, two additional fragments containing the three non–self-priming Polo-docking sites (3A), or all the Polo-docking sites substituted with alanine are presented. (**E** and **F**) In vitro kinase assays were performed with Cyclin B–Cdk1 or PLK-1 kinases and the NPP-10N^NUP98^ (513 to 821) fragments WT, 3A, or 8A tagged with the maltose-binding protein (MBP) as substrates. The samples were subjected to SDS–polyacrylamide gel electrophoresis (PAGE), followed by a far-Western ligand-binding assay using the PBD fused to GST (top). The bottom panel shows the Stain-Free Blot (Chemidoc, Bio-Rad) of the same membrane.

We have previously reported that NUPs of the central channel recruit PLK-1 to the NE (*20*). We thus investigated whether NPP-14^NUP214^, NPP-10N^NUP98^, and NPP-19^NUP53^, identified as specific phosphorylated binding partners of the PBD, also contribute to the recruitment of PLK-1 to the nuclear envelope in prophase. To this end, we used spinning disc confocal microscopy to monitor the recruitment of endogenously tagged PLK-1::sGFP to the nuclear envelope. RNAi-mediated depletion of NPP-14^NUP214^ did not affect PLK-1::sGFP recruitment to the nuclear envelope (Fig. 5A). However, partial depletion of NPP-10N^NUP98^ or NPP-19^NUP53^ drastically reduced PLK-1::sGFP signal at the nuclear envelope (Fig. 5A). Knocking down the central channel NUP NPP-1^NUP54^ resulted in a similar phenotype (Fig. 5A), consistent with our previous observations (*20*).

Central channel NUPs are anchored to the NPC via interaction with NPP-13^NUP93^, which is itself connected through NPP-19^NUP53^ binding (Fig. 1A). Thus, inhibition of *npp-19^NUP53^* might be expected to cause a reduction of the central channel NUPs at the nuclear envelope, and consequently of PLK-1::sGFP. Accordingly, *npp-19^NUP53^* inhibition did reduce NPP-1^NUP54^::GFP and GFP::NPP-11^NUP62^ signals at the nuclear envelope (Fig. 5, B and C). However, *npp-10N^NUP98^* depletion only mildly reduced central channel NUP levels at the nuclear envelope (Fig. 5, B and C) while profoundly reducing PLK-1::sGFP levels (Fig. 5A). This result suggested that NPP-10N^NUP98^ may directly contribute to PLK-1::sGFP recruitment, presumably by interacting with the PLK-1 PBD, which would be consistent with the NPP-10N^NUP98^ phosphorylated consensus Polo-docking sites we identified in the PBD pull-downs (Fig. 4). We tested this hypothesis using a far-Western ligand-binding assay. We pre-phosphorylated the NPP-10N^NUP98^ C-terminal fragment (513 to 821) using Cyclin B–Cdk1 or PLK-1 kinases, and then tested its ability to interact with the PLK-1 PBD. NPP-10N^NUP98^ (513 to 821) pre-phosphorylated by either kinase readily interacted with the PLK-1-PBD (Fig. 5, E and F, lane 3) but not with a version of the PBD carrying mutated phosphopincers (fig. S5, B and C, lane 3), demonstrating that this interaction is phospho-dependent. To test whether the Polo-docking sites accounted for PBD binding, we then analyzed NPP-10N^NUP98^ (513 to 821) fragments containing various alanine substitutions in the predicted Polo-docking sites.

The NPP-10N^NUP98^ fragment harbors three putative Polo-docking sites matching non–self-priming and binding (one Serine-Serine-Proline (SSP) and two Serine-Threonine-Proline (STP) sites) (Fig. 5D and fig. S5A), and we recovered phosphopeptides specifically phosphorylated on these three sites in the PLK-1 PBD pull-downs (Fig. 4 and table S3). In the far-Western binding assay, alanine substitutions of these sites abrogated NPP-10N^NUP98^ binding to the PLK-1 PBD, primed by Cyclin B–Cdk1 (Fig. 5E, lane 5). However, these alanine substitutions did not disrupt binding when NPP-10N^NUP98^ was primed by PLK-1 (Fig. 5F, lane 5), indicating that PLK-1 phosphorylates additional Polo-docking sites. Consistently, sequence analysis identified at least five other putative Polo-docking sites matching the self-priming and binding consensus (SSX/STX, where X is any aminoacid). Four of these five sites perfectly match PLK-1 phosphorylation sites (see the “Eukaryotic Linear Motif resource for Functional Sites in Proteins” website, http://elm.eu.org/) (fig. S5A), among which one was recovered phosphorylated in the PBD pull-downs (Fig. 4). Alanine substitutions of the eight Polo-docking sites fully abolished the binding of NPP-10N^NUP98^ to the PLK-1 PBD primed by PLK-1 (Fig. 5F, lane 7).

We conclude that Cyclin B–Cdk1 and PLK-1 both phosphorylate NPP-10N^NUP98^ on multiple Polo-docking sites in its unstructured region, thereby promoting its binding to the PLK-1 PBD. This interaction contributes to the recruitment of PLK-1 to the NPC, where it docks on NPP-10N^NUP98^. PLK-1 docking might be required for subsequent phosphorylations or it might disrupt NPP-10N^NUP98^ binding to its partners at the NPC, both of which would promote NPP-10N^NUP98^ dissociation from the NPC.

### Embryos expressing NPP-19^NUP53^ 10A defective in PLK-1 binding are deficient in inner ring complex disassembly

We then investigated the functional importance of the PLK-1 - NPP-19^NUP53^ interaction for the disassembly of the inner ring complex (Figs. 1E, 2D, and 3). To this end, we searched for all the NPP-19^NUP53^ Polo-docking sites responsible for interacting with the PLK-1 PBD to engineer an NPP-19^NUP53^ variant specifically defective in PLK-1 binding and evaluate its impact on NPC disassembly in vivo.

**Fig. 6.**
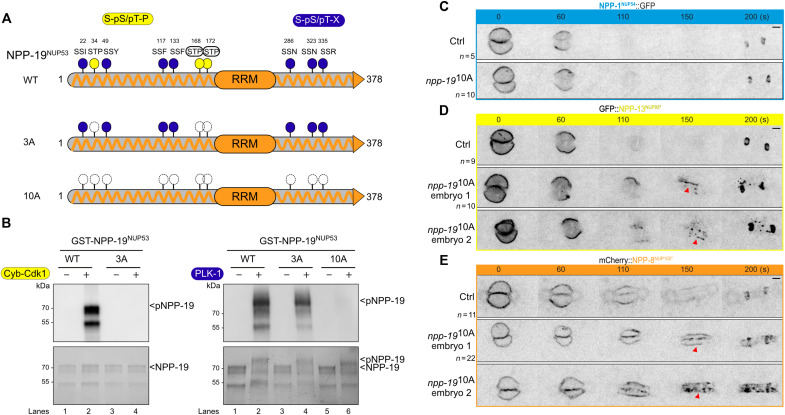
Multisite phosphorylation of NPP-19^NUP53^ is required for the disassembly of the inner ring complex. (**A**) NPP-19^NUP53^ contains three Polo-docking sites matching the consensus for non–self-priming (yellow) and seven sites matching the consensus for self-priming (blue). Besides the WT, two additional NPP-19^NUP53^ variants containing the three non–self-priming Polo-docking sites (3A) or all the Polo-docking sites (10A) substituted with alanine are presented. The sites that are circled have been identified in vivo. (**B**) In vitro kinase assays were performed with Cyclin B–Cdk1 or PLK-1 kinases and the NPP-19^NUP53^ WT, 3A, or 10A tagged with the GST as substrates. The samples were subjected to SDS-PAGE, followed by a far-Western ligand-binding assay using the PBD fused to GST (top). The bottom panel shows the Stain-Free Blot (Chemidoc, Bio-Rad) of the same membrane. (**C** to **E**) Micrographs of control (top) or *npp-19^NUP53^* 10A mutant embryos (bottom) expressing NPP-1^NUP54^::GFP (C), GFP::NPP-13^NUP93^ (D), or GFP::NPP-8^NUP155^ (E) during mitosis. Timings in seconds are relative to pronuclei juxtaposition (0). All panels are at the same magnification. Scale bar, 3 μm. Data were collected from three independent experiments.

NPP-19^NUP53^ contains 10 potential Polo-docking sites relatively well conserved in nematode species (fig. S6); seven sites match the consensus for self-priming (S-S/T-X), whereas three sites match the consensus for non–self-priming (S-S/T-P) (Fig. 6A), one of which was identified in the PLK-1 PBD pull-downs (Fig. 4 and table 1). Alanine substitution of the three non–self-priming sites (S-A-P) abolished NPP-19^NUP53^ binding to the Plk1 PBD primed by Cyclin B–Cdk1 in a far-Western binding assay (Fig. 6B, left, lane 4). However, these three alanine substitutions did not notably affect the interaction when PLK-1 was the priming kinase (Fig. 6B, right, lane 4). Alanine substitutions of all 10 Polo-docking sites were required to eliminate the ability of PLK-1 to trigger the interaction between NPP-19 10A and the Plk1 PBD (Fig. 6B, lane 6). We obtained similar results using the shorter and more soluble NPP-19^NUP53^ (1 to 301) fragment tagged with 6xHis (fig. S7, C and D). These studies indicate that Cyclin B–Cdk1 and PLK-1 both prime NPP-19^NUP53^ binding to the Plk1 PBD via the phosphorylation of multiple Polo-docking sites, similar to NPP-10N^NUP98^.

Next, we used CRISPR-Cas9–based mutagenesis to engineer an *npp-19^NUP53^* allele carrying alanine substitutions in the 10 Polo-docking sites to investigate the functional consequences of a defect in PLK-1 binding to NPP-19^NUP53^ for NPC disassembly in vivo. These 10 alanine substitutions did not alter NPP-19^NUP53^ stability nor its ability to localize to the NPCs, as revealed by the normal levels of expression and the proper localization of NPP-19^NUP53^ 10A to the nuclear envelope (fig. S8, A and B). Furthermore, *npp-19*^NUP53^ 10A mutant embryos were viable and did not present the major cell cycle defects previously observed in *npp-19^NUP53^* loss-of-function mutant embryos (fig. S8C) (*39*). PLK-1::sGFP localized to the nuclear envelope in prophase in these *npp-19^NUP53^* 10A mutant embryos (fig. S8D), indicating that the defect in PLK-1::sGFP recruitment to the NE previously observed in *npp-19(RNAi)* embryos (Fig. 5A) is due to the loss of NPP-19–associated PLK-1 recruiters (see Discussion). The dynamics of the central channel NUP NPP-1^NUP54^::GFP was also largely unaffected in *npp-19^NUP53^* 10A mutant embryos during mitosis (Fig. 6C). However, disassembly of the inner ring complex NUPs GFP::NPP-13^NUP93^ and mCherry::NPP-8^NUP155^, which are direct binding partners of NPP-19^NUP53^, was compromised. GFP::NPP-13^NUP93^ persisted at the nuclear envelope during mitosis in *npp-19^NUP53^* 10A mutant embryos (Fig. 6D, red arrowheads). Likewise, mCherry::NPP-8^NUP155^ remained associated with the nuclear envelope throughout mitosis in *npp-19^NUP53^* 10A mutant embryos and failed to relocalize to the centriculum, as observed in control embryos (Fig. 6E). These observations indicate that phospho-dependent recruitment of PLK-1 to NPP-19^NUP53^, at the core of the NPCs, is required for efficient inner ring complex disassembly.

## DISCUSSION

**Fig. 7.**
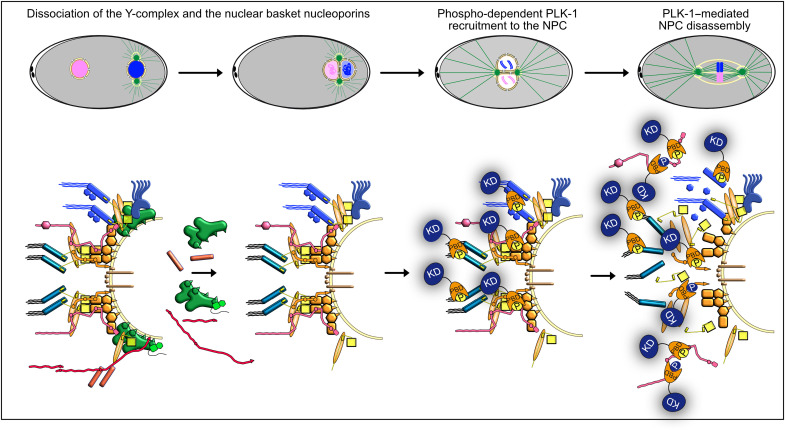
PLK-1 is recruited to multiple multivalent nucleoporins to dismantle the nuclear pore complexes. Dynamics of NPCs during the first mitotic division of the *C. elegans* embryo. During pronuclear migration and meeting, the Y-complex and nuclear basket NUPs leave the NPC. PLK-1 is then recruited to the NPC, 250 s before anaphase onset, via its PBD domain in a phospho-dependent manner just before NEBD when the two pronuclei are juxtaposed. PLK-1 is recruited via the multivalent NUPs NPP-10N^NUP98^, NPP-19^NUP53^, the central channel NUPs, and by NPP-14^NUP214^ phosphorylated at Polo-docking sites by Cyclin B–Cdk1 and PLK-1 itself. PLK-1 recruitment and docking on NUPs promote NPC disassembly.

Using a combination of live imaging, biochemistry, and phosphoproteomics, we characterized NPC disassembly in the one-cell *C. elegans* embryos and dissected the role of PLK-1 in this process. First, we show unequivocally that PLK-1 promotes NPC disassembly independently of lamina depolymerization. Then, we demonstrate that NPC disassembly is a stepwise process that involves PLK-1–dependent and –independent steps. Our findings, summarized in Fig. 7, indicate that, after the departure of the Y-complex and the nuclear basket from the NPC, PLK-1 is dynamically recruited to and phosphorylates IDRs of several multivalent linker NUPs to trigger the disassembly of multiple NPC subcomplexes, including the cytoplasmic filaments, the inner ring complex, and the central channel.

### Characterization of NPC disassembly in early *C. elegans* embryo

NPCs are massive structures assembled from various NUP subcomplexes that dynamically assemble and disassemble during the cell cycle (*5*). While the mechanisms driving NPC assembly are beginning to emerge (*61*, *62*), how NPCs are disassembled in various organisms is not well understood. In particular, the order of component disassembly is still elusive. This question has been mainly addressed using cultured human cells (*8*). In this context, NUP98, the gatekeeper of the nuclear pore, is the first NUP to leave the NPC (*9*, *11*, *63*) followed by the rapid and nearly synchronous disassembly of the other NUPs (*11*). NUP98 maintains the permeability barrier of the NPC by virtue of the self-cohesive interactions of its N-terminal GLFG domain (*6*), and thus, its departure from the NPCs facilitates nuclear permeabilization at the onset of mitosis (*9*, *11*).

In the early *C. elegans* embryo, the order of NUP disassembly had yet to be investigated. We found here that the nuclear basket NUPs NPP-7^NUP153^, NPP21^TPR^, and the Y-complex subunits MEL-28^ELYS^, NPP-5^NUP107^, NPP-6^NUP160^, and NPP-18^SEH-1^ all leave the NPC early in prophase, while the two pronuclei are still migrating or are about to meet at the posterior pole of the embryo, well before their nuclear envelope becomes permeable. It is only later, when NPP-10N^NUP98^, the central channel and the inner ring complex NUPs leave the NPC that the permeability barrier is breached. Thus, the order of NPC disassembly in the *C. elegans* embryo appears to be different from that of human cells, with some NUP subcomplexes leaving the NPC earlier without affecting the permeability barrier. This is a physiological demonstration that the NPC can lose NUPs, and specifically the Y-complex, without compromising the diffusion barrier. This echoes a study in human cells in which auxin-mediated depletion of the entire Y-complex had little impact on the stability of the inner ring complex and the central channel NUPs (*64*).

The functional relevance of the earlier departure of the nuclear basket and the Y-complex subunits from the NPC in *C. elegans* as compared to human cells is presently unclear. A portion of the Y-complex relocalizes to the kinetochore during mitosis in human cells (*65*) and in *C. elegans* (*66*) where it contributes to kinetochore assembly and chromosome segregation (*66*, *67*). Perhaps, the holocentric nature of kinetochores of *C. elegans* requires earlier recruitment of the Y subunits for their proper assembly.

Regarding the early departure of the nuclear basket from the NPC, recent studies in budding yeast revealed that nuclear basket assembly and maintenance depend on active nuclear mRNA metabolism, particularly processing and polyadenylation of transcripts (*68*). It is worth mentioning that the first division of the embryo occurs in the absence of transcription, which may explain the absence of a nuclear basket at the nuclear pores during the first interphase.

### PLK-1 is independently recruited to multiple nucleoporins

While PLK-1 is not required for the removal of the nuclear basket and the Y-complex subunits from the NPC, it is critically required for the disassembly of the central channel, the cytoplasmic filaments, and the inner ring complex. After activation in the cytoplasm via phosphorylation of its activation segment (*69*, *70*), PLK-1 is recruited to the nuclear envelope in prophase (*10*, *20*), before they are permeable. Our results suggest that, after its delivery to the NPC, PLK-1 uses its PBD to dock on the IDRs of multiple NUPs, phosphorylated on Polo-docking sites by Cyclin-Cdk1 and PLK-1 itself. PLK-1 docks not only on NPP-19^NUP53^ as in human cells (*10*) but also on NPP-10N^NUP98^, NPP-14^NUP214^, and the central channel NUPs phosphorylated on multiple Polo-docking sites. Accordingly, alanine substitution of the 10 Polo-docking sites in NPP-19^NUP53^, which abrogates its binding to the PLK-1 PBD in vitro, is not sufficient to prevent PLK-1 recruitment to the NPC, clearly indicating the presence of additional PLK-1 recruiters at the NPC in *C. elegans*. NPP-1^Nup54^, NPP-4^Nup58^, NPP-11^Nup62^, and NPP-10N^NUP98^, which line the central channel, are the most accessible NUPs and thus likely act as primary recruiters of PLK-1 at the NPC.

These NUPs all possess phosphorylated Polo-docking sites matching both non–self- and self-priming and binding motifs, suggesting that Cyclin B–Cdk1 might dictate the initial binding of the first PLK-1 molecules before PLK-1 itself promotes its own docking and further accumulation required for NPC disassembly. PLK-1 might also dock on the transmembrane NUP NPP-12^gp210^, which is required for efficient NPC disassembly and lamina depolymerization (*24*), possibly via phosphorylation of its C-terminal tail (*25*). Unfortunately, tagging NPP-12^gp210^ was deleterious and partially inactivated the proteins which precluded a functional analysis of NPP-12^gp210^ phosphorylation for NPC disassembly.

### How does PLK-1 trigger NPC disassembly?

In vitro binding experiments and structural studies have shown that NUP98 and NUP53 interact with several NUPs and thereby serve as multivalent linkers within the NPC scaffold (*35*, *40*–*44*, *71*). NUP98 binds through its C-terminal NPC targeting domain subunits of the Y-complex and members of the inner ring complex notably NUP205 and NUP155 (*43*, *44*). Likewise, NUP53 uses multiple regions to interact with distinct subunits of the inner ring complex including NUP93, NUP155, and NUP205 but also with the transmembrane protein NDC1 (*35*, *40*–*44*, *60*, *71*, *72*). Previous studies in human cells showed that NUP98 and NUP53 are targeted by multiple kinases including Cyclin B-Cdk1, NIMA (Never In Mitosis A), and Plk1 (*9*, *10*). Cyclin-Cdk1 phosphorylates NUP98 on 8 sites and NUP53 on 16 sites (figs. S5 and S6) (*9*, *10*). Notably, the introduction in human NUP98 of two phosphomimetic residues is sufficient to abrogate its binding to the NUP155 C-terminal domain (*9*, *43*). Structural analysis of the NUP98-NUP155 interface using *Chaetomium thermophilium* proteins has revealed a possible mechanism that could explain how the phosphorylation of these two residues affects this interaction. NUP98 phosphorylation induces structural rearrangements destroying the hydrophobic interface required for NUP155 binding (*9*, *43*). This type of mechanism is ideally suited for the unzipping of NUP-NUP interactions because the phosphorylatable residues are directly accessible to the mitotic kinases. Another (nonexclusive) possibility is that the docking of PLK-1 on NUPs competes with and displaces interactions between these NUPs and their partners. In support of this hypothesis, we have noticed that several Polo-docking sites in NPP-19^NUP53^ localize within or near conserved regions of interaction among NUP53 and its binding partners (fig. S6). For instance, NUP53 binds NUP93 via a linear 10-residue motif (SGAPPVRSIY), which recognizes a hydrophobic surface on the NUP93 solenoid (*44*). This motif, which is evolutionarily conserved between NUP53 orthologues, is immediately flanked by Polo-docking sites, both in *C. elegans* and in human NUP53 (fig. S6). Furthermore, PLK-1 can dimerize on substrates containing several Polo-docking sites (*73*, *74*), which would increase the steric hindrance at the NPC. PLK-1 docking on NUPs might also allosterically destabilize NPC subcomplexes, as reported in the case of Kap121 binding to Nup53 (*75*). We thus speculate that PLK-1 docking on NPP-19^NUP53^ by itself might contribute to displacing NPP-19^NUP53^-NPP-13^NUP93^ interactions. Similarly, PLK-1 docking might displace the interactions between NPP-19^NUP53^ and NPP-8^NUP155^ or NPP-3^NUP205^ because several Polo-docking sites are also present in the regions of interactions (fig. S6). Last, PLK-1 docking on NPP-10N^NUP98^ could similarly disrupt its interaction with partners (fig. S5).

Beyond NPP-19^NUP53^ and NPP-10N^NUP98^, PLK-1 appears to have multiple independent targets at the NPC in *C. elegans* including NPP-14^NUP214^ and the central channel NUPs. This is also supported by our finding that worms expressing the NPP-19^NUP53^ 10A variant specifically defective in PLK-1 docking display problems in inner ring disassembly but not in central channel NUP disassembly.

Comprehensive identification of all the sites phosphorylated by PLK-1 on NUPs and the functional dissection of their roles in NPC disassembly will be a major future challenge. Obtaining or modeling the structure of the *C. elegans* NPC would be helpful in this endeavor.

Overall, despite some noticeable differences between the mechanisms of NPC disassembly in human cells versus *C. elegans*, NPP-10N^NUP98^ and NPP-19^NUP53^ are major phosphorylation targets in both species. The phosphorylated sites on NPP-10N^NUP98^ and NPP-19^NUP53^ are poorly conserved between human and *C. elegans* proteins, and targeted by different kinases, yet they are concentrated in the exact same regions. Phosphorylation of IDRs of multivalent linker NUPs thus appears to be an evolutionarily conserved mechanism driving NPC disassembly during mitosis.

### Limitations of the study

Monitoring the live dynamics of endogenous NUPs requires their tagging with large fluorescent markers, NeonGreen, mCherry, or GFP. While all the lines used in this study expressing endogenously tagged NUPs are viable, some of them are slightly compromised in function, in particular, lines expressing mCherry::NPP-8^NUP155^ or NeonGreen::NPP-10N^NUP98^. For instance, NeonGreen::NPP-10N^NUP98^ was synthetic lethal with *npp-14^NUP214^* null. We also noticed some cumulative defects when trying to generate lines coexpressing two tagged NUPs, in particular combining GFP::NPP-13^NUP93^ and mCherry::NPP-8^NUP155^ that belong to the same complex. This precluded the simultaneous visualization of multiple tagged NUPs in the same embryo. Although we have not tested all combinations, care should be taken when analyzing the dynamics of NUPs in strains coexpressing multiple tagged NUPs.

Unfortunately, we could not look at NPP-19 10A during mitotic progression, as we do not have an endogenously tagged fluorescent version, and it is technically challenging to visualize persisting NPP-19 foci during mitosis by immunostaining using our antibody. Last, it is worth mentioning that the *C. elegans* phosphoproteome has been generated from embryonic extracts that are not synchronized, and thus, the reported phosphosites on NUPs might be present in interphase or mitosis.

## MATERIALS AND METHODS

### Nematode strains and RNAi

*C. elegans* strains were cultured and maintained using standard procedures (*76*). RNAi was performed by the feeding method using HT115 bacteria essentially as described (*77*) except that 2 mM isopropyl-β-D-thiogalactopyranoside (IPTG) was added to the nematode growth media (NGM) plates and in the bacterial culture just before seeding the bacteria. As a control, animals were exposed to HT115 bacteria harboring the empty feeding vector L4440 (mock RNAi). RNAi clones were obtained from the Arhinger library (Open Source BioScience). For partial *npp-1* and *npp-14* inactivation, L4 animals were fed with bacteria for 24 hours at 20°C. For partial *npp-19* and *npp-10* inactivation, L4 animals were fed with bacteria for 7 to 9 h at 20°C. For partial *plk-1* inactivation, L4 animals were fed with bacteria for 7 to 9 hours at 23°C.

### CRISPR-Cas9 genome engineering

To endogenously tag *npp-21*, a double-stranded polymerase chain reaction (PCR) product encoding green fluorescent protein (GFP) and flanked by 33- to 35-nucleotide sequences with homology to *npp-21* was synthesized in two steps using first primers B767 and B783 followed by primers B769 and B784. Plasmid pBN221 *npp-21* single-guide RNA (sgRNA) was generated by whole-plasmid PCR amplification using pBN180 (*59*) as a template and primers B635 and B782. Strain BN424 *dpy-10(cn64) npp-21::gfp* was generated by microinjection of the GFP PCR fragment together with plasmids pBN221 *npp-21* sgRNA, pBN207 *dpy-10* sgRNA (*59*), #1286 *eft-3p::Cas9* (*78*), and *dpy-10* single-stranded oligodeoxynucleotide (ssODN) B725, into the gonads of N2 young adult hermaphrodites (*79*). Next, *npp-21::gfp* was segregated from *dpy-10(cn64)* by successive crosses with BN189 and N2 to generate BN1062 *npp-21::gfp lmn-1p::mCh::his-58*.

Targeting plasmids pBN317 *g>f>p::npp-19* and pBN394 *g>f>p::npp-24* [“>“denotes an Flippase Recognition Target (FRT) site] for SapTrap CRISPR-Cas9 gene modification were obtained as described (*80*) using primers B969-B974 and B1085-B1090, respectively, as well as pMLS256, pMLS288, and pBN312 as donor and receptor plasmids (*80*, *81*). Plasmid pBN404 *g>f>p::npp-19* was derived from pBN317 by whole-plasmid PCR using primers B1070 + B1071 to eliminate the unc-119(+) gene. Strain BN739 *npp-24(bq15[g>f>p::npp-24])* II was generated by microinjection of targeting plasmid pBN394 into the gonads of HT1593 *unc-119(ed3)* III young adult hermaphrodites (*80*). Targeting plasmid was injected at 65 ng/μl together with plasmids #1286 *eft-3p::Cas9* (*78*) (25 ng/μl), pBN1 (10 ng/μl), pCFJ90 (2.5 ng/μl), and pCFJ104 (5 ng/μl). Successful modification of the target loci was confirmed by the detection of fluorescent fusion proteins in the nuclear envelope throughout the body of WT moving animals without ectopic mCherry expression from the coinjection markers. The *unc-119(+)* selection marker was next excised by microinjection with Cre expression plasmid pMLS328 (*80*). Last, uncoordinated hermaphrodites were outcrossed to N2 males to remove the *unc-119(ed3)* allele.

Endogenous tagging of *npp-19* was performed based on protocols for nested CRISPR (*82*) and “hybrid” partially single-stranded DNA donors (*83*). First, we inserted a ssODN containing gfp 5′ and 3′ sequences immediately after the *npp-19* ATG initiation codon by microinjection of ssODNs B1205 (1 μM) and B1304 (4 μM), CRISPR RNA (crRNA)s B1206 (2.5 μM) and B1069 (10 μM), trRNA (12.5 μM) and Cas9 (3 μM) into the gonads of HT1593 *unc-119(ed3)* III young adult hermaphrodites. WT-moving F1s (heterozygous for unc-119) were isolated, left to lay eggs, and analyzed by PCR with primers B1305 and B1306. A sequence-verified line was established (*npp-19(bq27[ssODN B1304]) II; unc-119(ed3) III*) and microinjected with ssODN B1205 (1 μM), crRNAs B1206 (2.5 μM) and B1311 (10 μM), trRNA (12.5 μM), Cas9 (3 μM), and a hybrid PCR product (~0.1 μg/μl) generated by mixing PCR A (amplified from pBN404 with primers B1266 and B1267) and PCR B (amplified from pBN404 with primers B1268 and B1269). Successful candidates were verified by the expression of GFP::NPP-19 in the nuclear envelope in all cell types and crossed with BN189 to generate BN1018 *g>f>p::npp-19 lmn-1p::mCherry::his-58*.

### Generation of *npp-19^NUP53^* 10A allele

The worm strain expressing *npp-19* 10A was generated via CRISPR-Cas9 insertion of a recoded genomic DNA (gDNA) of *npp-19* harboring the mutation of the residues S22, T34, S48, S117, S133, T168, T172, S286, S323, and S335 into alanines (SunyBiotech). The recoded part of *npp-19* gDNA consisted of the nucleotides 271 to 936 which are targeted by the RNAi against *npp-19* from the Arhinger library. WT and RNAi-resistant sequences are as follows.

### Wild-type sequence (nucleotides 271 to 936)

TAGCCCGCTCAACACTGCATCGGCACCATGTTCGGATATTTTCGCTGTTTCTGCACCGGCAGTGCCGCAGCATTTGAAGGATACACCGGGCTCTAAATCAGTTCATTGGTCTCCATCTTTGGTGCAATCTGGTGAAAAGTCGGCGGCACAAACACAGAATACACCTGCCAACTTGTCTTTCGGAGGAAATTCATCATTTTCGGCGCCGACAAAGCCAGCTCCTCAGTCGATTCAGACATCATCTTTCGGTGGTCAAGCGATGCATGgttagaatttacattttttcgcgaatatttaaaataaaattgattttcagCACCACCTCTTCGATCTCTTCGCGACAAAGTTGAACCAGCGAAAAAGATCTCCAGACGAAATACATTCACTGCCAGATCAACACCACTTTCCACTCCAATCACTCAACGAGTCACATCCAGGTTGGCTGAAGCAGAAGAACAACCAATGGAAGAAGAAGCCGACGCAGCTGATACCTGGGTCACTGTTTTTGGATTTCAGCCAAGCCAAGTGTCGATTTTGTTGAATTTATTCTCGCGGCACGGGGAAGTAGTTAGTCATCAGACTCCATCAAAAGGAAACTTCATACATATGCGCTATTCGTGTGTCACACACGCTCAACAAGCTATTTCTCGAAACGGAACTCTCCTC.

### Recoded sequence (nucleotides 271 to 936)

TTCACCTCTCAACACTGCGAGCGCGCCATGCAGCGATATTTTCGCCGTTTCTGCGCCTGCCGTACCTCAGCATTTGAAGGATACGCCTGGATCTAAAAGCGTCCATTGGTCTCCATCTCTGGTTCAATCTGGCGAAAAGAGCGCGGCACAAACGCAAAATACGCCGGCTAACCTGTCCTTCGGAGGAAATAGCGCCTTCTCGGCGCCTACGAAGCCAGCCCCGCAGTCCATTCAGACGAGCGCTTTCGGCGGTCAAGCGATGCATGgttagaatttacattttttcgcgaatatttaaaataaaattgattttcagCACCACCGCTTAGGTCTCTCAGAGATAAAGTCGAACCAGCAAAAAAGATCTCTAGAAGGAATACGTTCACTGCCCGCAGCgCCCCACTCTCCgCACCAATCACTCAAAGGGTCACGTCCCGGTTGGCCGAGGCGGAGGAGCAACCAATGGAGGAAGAAGCTGACGCGGCCGACACCTGGGTTACAGTTTTTGGATTTCAACCATCACAAGTAAGCATTTTGTTGAATCTATTCAGCAGGCACGGCGAAGTGGTTTCGCATCAGACACCAAGCAAAGGAAACTTCATTCACATGCGCTATAGCTGTGTCACGCACGCCCAACAAGCCATTTCTAGGAACGGAACTCTTCTC.

### Molecular biology

The plasmids and oligonucleotides used in this study are listed in the key resource table. Gateway cloning was performed according to the manufacturer’s instructions (Invitrogen). All the constructs were verified by DNA sequencing (Eurofins Genomics).

### Biochemical assays

Western blot analysis was performed using standard procedures.

### Protein production and purification

#### GST-NPP-19NUP53

 The expression of NPP-19 full-length N-terminally fused to GST was induced by the addition of 1 mM IPTG to 1 liter of LB cultures of exponentially growing *Escherichia coli* BL21 DE3 pLysS (Invitrogen) strain [optical density (OD) = 0.6], before incubation for 3 hours at 25°C. After pelleting by centrifugation, the bacteria were resuspended in lysis buffer [0.5 M NaCl, 5% glycerol, 50 mM tris-HCl (pH 8), 1× protease inhibitors (cOmplete Protease Inhibitor Cocktail tablets, Roche)] before lysis by sonication. The soluble portion of the lysate was loaded on 500 μl of glutathione (reduced form) beads (slurry) and incubated for 1 hour. The beads were washed with at least 100 ml of lysis buffer and the proteins were eluted with 20 mM glutathione solution in 50 mM tris-HCl (pH 8). Proteins were aliquoted and flash-frozen in liquid nitrogen and stored at −80°C.

#### 6x(His)-NPP-19^NUP53^ (1 to 301)

The expression of NPP-19 (1 to 301) fragment N-terminally fused to a 6xHis tag was induced by the addition of 1 mM IPTG to 1 liter of LB cultures of exponentially growing *E. coli* BL21 DE3 pLysS (Invitrogen) strain (OD = 0.6) before incubation for 3 hours at 25°C. After pelleting by centrifugation, the bacteria were resuspended in lysis buffer [0.5 M NaCl, 5% glycerol, 50 mM tris-HCl (pH 8), and 1× protease inhibitors) before lysis by sonication. The soluble portion of the lysate was loaded on a 1-ml Hi-Trap Column (GE Healthcare) previously loaded with a 1 M NiCl_2_ solution. The column was washed with 10 volumes of lysis buffer, and bound proteins were eluted in a lysis buffer containing 600 mM imidazole (pH 8). After dialysis with lysis buffer to remove imidazole, proteins were concentrated, aliquoted, flash-frozen in liquid nitrogen, and stored at −80°C.

#### MBP-NPP-10NNUP98 (513 to 821)

The expression of NPP-10N N-terminally fused to maltose-binding protein (MBP) was induced by the addition of 1 mM IPTG to 1 liter of LB cultures of exponentially growing *E. coli* BL21 DE3 pLysS (Invitrogen) strain (OD = 0.6) before incubation for 3 hours at 25°C. After pelleting by centrifugation, the bacteria were resuspended in lysis buffer [0.5 M NaCl, 5% glycerol, 50 mM tris-HCl (pH 8), and 1× protease inhibitors] before lysis by sonication. The soluble portion of the lysate was loaded on a 1-ml MBP-Trap Column (GE, healthcare). The column was washed with 10 volumes of lysis buffer, and bound proteins were eluted in a lysis buffer containing 20 mM maltose (pH 8). Proteins were aliquoted and flash-frozen in liquid nitrogen and stored at −80°C.

#### 6x(His)-PLK-1

To produce *C. elegans* 6x(His)-PLK-1, insect Sf9 cells were infected with appropriate baculovirus and then lysed in lysis buffer [phosphate-buffered saline (PBS) (pH 7.2), 250 mM NaCl, 30 mM imidazole, and protease and phosphatase inhibitors (Roche)], passing the cell suspension 30 times through a 21-gauge syringe needle. The lysate was clarified by centrifugation for 10 min at 16,000*g*, and the supernatant was injected into the HiTrap Chelating HP column loaded with nickel sulfate (GE Healthcare). Proteins were eluted by an imidazole gradient using a fast protein LC Äkta System (GE, Healthcare). Most purified elution fractions were pooled, diluted volume to volume in the lysis buffer without imidazole and containing 50% glycerol, concentrated on a centrifugal concentrator (Vivaspin VS15RH12; Vivaproducts), flash-frozen in liquid nitrogen, and stored at −80°C.

#### GST-Plk1 PBD (H. s) WT and GST-Plk1 PBD H538A/K540M

The human GST-Plk1 PBD WT or phosphate pincer (GST-Plk1 PBD H538A/K540M) mutant fusion proteins were induced by the addition of 1 mM IPTG to 1 liter of LB cultures of exponentially growing *E. coli* BL21 DE3 pLysS (Invitrogen) strain (OD = 0.6) before incubation for 3 hours at 25°C. After pelleting by centrifugation, the bacteria were resuspended in lysis buffer [10 mM tris (pH 8), 150 mM NaCl, 1 mM EDTA, 5 mM dithiothreitol (DTT), 0.05% NP-40, 1 mM phenylmethylsulfonyl fluoride, and 1× protease inhibitors) before lysis by sonication. The soluble portion of the lysate was loaded on a 1-ml GST-Trap Column (GE, healthcare). After extensive washes, the bound proteins were eluted in a lysis buffer containing 20 mM glutathione (pH 8). Proteins were aliquoted and flash-frozen in liquid nitrogen and stored at −80°C.

### Far-Western ligand-binding assay

GST-NPP-19 full-length, GST-NPP-19 (1 to 301), or MBP-NPP-10N (513 to 821) fragments, phosphorylated in vitro by PLK-1 or Cyclin B–Cdk1 as described (*20*), were separated on stain-free SDS–polyacrylamide gel electrophoresis 10% gel (Bio-Rad). The gel was imaged and then transferred to a polyvinylidene difluoride membrane (0.45 μm) for 1.5 hours at 90 V. After saturation overnight at 4°C in blocking solution (4% milk in tris-buffered saline with 0.1% Tween 20), the membranes were incubated with 2 μg of GST-PBD WT or the GST-PBD H538A/K540M mutant of Plk1 (version of the PBD was unable to bind phosphopeptides, negative control) during 5 hours at 4°C. After extensive washing steps (every 15 min for at least 3 hours) with the blocking solution at 4°C, the membrane was incubated overnight at 4°C with a human Plk1 antibody (1 of 1000) in a typical Western blot experiment to reveal the GST-PBD immobilized on the membrane.

### *C. elegans* embryonic extracts and GST-PBD pull-downs

#### Preparation of embryonic extracts

Liquid cultures of N2 worms synchronized at the L1 stage were grown on an S medium using *E. coli* HB101 bacteria as a food source (*84*, *85*). Worms were harvested at the young adult stage by filtration using nylon mesh (Sefar Nitex, 35-mm mesh). After several washing steps, embryos were isolated by bleaching, washed several times with M9 buffer, and resuspended in one volume of 100 mM NaCl, frozen as beads in liquid nitrogen following cryo-lysis by cryogenic grinding (RETSCH MM400), and kept at −80°C. Three cryo-lysates were prepared from three independent liquid cultures (A, B, and C). For pull-down experiments, 500 mg of each embryonic cryo-lysed was resuspended in 1 ml of lysis buffer [25 mM tris-HCl (pH 7.5), 100 mM NaCl, 2 mM MgCl2, 1 mM DTT, phosphatase inhibitors (PhosSTOP EASYpack, Roche), and protease inhibitors (cOmplete Protease Inhibitor Cocktail tablets, Roche)] and incubated with 1600 U of benzonase nuclease (Sigma-Aldrich) for 30 min at 4°C. The lysates were clarified by centrifugation (twice 13,000*g*, 20 min at 4C°) to obtain embryonic extracts (roughly 3 mg/ml).

#### Preparation of the GST-PBD affinity matrix

GST-PBD WT or GST-PBD H538A/K540M mutant (GST-PBDmut) affinity matrix was obtained by incubating 165 μg of GST-PBDwt or GST-PBDmut proteins with 150 μl of glutathione beads in binding buffer [50 mM tris-Cl (pH 8) and 500 mM NaCl]. After three washing steps, the beads were equilibrated in a lysis buffer.

#### Loading on GST-PBD beads

Five hundred microliters of each embryonic extract was applied to GST-PBDwt or GST-PBDmut affinity matrix for 4 hours at 4°C. After washing the beads with a lysis buffer, the proteins were digested on beads by trypsin, the phosphopeptides were purified by Fe-NTA immobilized metal ion affinity chromatography and subjected to MS analysis. Fifty microliters of the same embryonic extracts (input) was digested by trypsin, and the phosphopeptides were purified by Fe-NTA and subjected to MS analysis.

### Mass spectrometry analysis

#### Material

MS grade acetonitrile (ACN), MS grade H_2_O, MS grade formic acid (FA), and High-Select Fe-NTA Phosphopeptide Enrichment Kit were from Thermo Fisher Scientific (Waltham, MA, USA). Sequencing-grade trypsin was from Promega (Madison, WI, USA). Trifluoroacetic acid (TFA), DTT, iodoactetamide (IAA), and ammonium bicarbonate (NH_4_HCO_3_) were from Sigma-Aldrich (St. Louis, MO, USA). Sep-Pak classic C18 cartridges were from Waters (Milford, MA, USA).

#### Samples preparation before LC-MS/MS analysis

One hundred fifty micrograms of protein from embryonic extracts was precipitated using a six-time volume of cold acetone (−20°C). Vortexed tubes were incubated overnight at −20°C, and then centrifuged for 10 min at 11,000 rpm at 4°C. The protein pellets were dissolved in 8 M urea and a 25 mM NH_4_HCO_3_ buffer. Samples were then reduced with 10 mM DTT final and alkylated with 20 mM IAA, and then diluted below 1 M urea before trypsin digestion overnight at 37°C (enzyme/substrate ratio of 1/50). Beads from pull-down experiments were incubated overnight at 37°C with 100 μl of 25 mM NH_4_HCO_3_ buffer containing 4 μg of sequencing-grade trypsin.

The digested peptides were desalted on Sep-Pak classic C18 cartridges. Cartridges were sequentially washed with 2 ml of methanol, 2 ml of a 70% (v/v) aqueous ACN containing 1% (v/v) TFA, and equilibrated with 2 ml of 1% (v/v) aqueous TFA. The digested peptides were acidified with 1% (v/v) aqueous TFA, applied to the column, and washed with 2× 1 ml of a 1% (v/v) aqueous FA. Peptides were eluted by applying 2× 500 μl of a 70% (v/v) aqueous ACN containing 0.1% (v/v) FA. Eluates were vacuum-dried. Phosphopeptide enrichment was then performed according to the manufacturer’s procedure starting from the lyophilized peptide samples. Eluted phosphopeptides were loaded and desalted on Evotips provided by Evosep (Odense, Denmark) according to the manufacturer’s procedure before LC-MS/MS analysis.

#### LC-MS/MS acquisition

Samples were analyzed on a timsTOF Pro 2 mass spectrometer (Bruker Daltonics, Bremen, Germany) coupled to an Evosep one system (Evosep, Odense, Denmark) operating with the 30SPD method developed by the manufacturer. Briefly, the method is based on a 44-min gradient and a total cycle time of 48 min with a C18 analytical column (0.15 × 150 mm, 1.9-μm beads, ref. EV-1106) equilibrated at 40°C and operated at a flow rate of 500 nl/min. H_2_O/0.1% FA was used as solvent A and ACN/ 0.1% FA as solvent B.

The timsTOF Pro 2 was operated in PASEF mode (*86*) over a 1.3-s cycle time. Mass spectra for MS and MS/MS scans were recorded between 100 and 1700 mass/charge ratio (*m/z*). Ion mobility was set to 0.75 to 1.25 V·s/cm^2^ over a ramp time of 180 ms. The data-dependent acquisition was performed using six PASEF MS/MS scans per cycle with a near 100% duty cycle. Low *m/z* and singly charged ions were excluded from PASEF precursor selection by applying a filter in the *m/z* and ion mobility space. The dynamic exclusion was activated and set to 0.8 min, and a target value of 16,000 was specified with an intensity threshold of 1000. Collisional energy was ramped stepwise as a function of ion mobility.

### Data analysis

MS raw files were processed using PEAKS Online X (build 1.6, Bioinformatics Solutions Inc.). Data were searched against the *C. elegans* Wormpep release 2021_07 database (total entries, 28,411). Parent mass tolerance was set to 20 parts per million (ppm), with fragment mass tolerance at 0.05 Da. Specific tryptic cleavage was selected and a maximum of two missed cleavages was authorized. For identification, the following posttranslational modifications were included: acetyl (Protein N-term), oxidation (M), deamidation (NQ), and phosphorylation (STY) as variables and half of a disulfide bridge (C) (pull-down) or carbamidomethylation (C) (total lysates) as fixed. Identifications were filtered based on a 1% FDR (false discovery rate) threshold at both peptide and protein group levels. Label-free quantification was performed using the PEAKS Online X quantification module, allowing a mass tolerance of 20 ppm, a collision cross section (CCS) error tolerance of 0.05 and 0.5 min of retention time shift tolerance for match between runs. Protein abundance was inferred using the top N peptide method and Total Ion Chromatogram (TIC) was used for normalization. Multivariate statistics on protein or peptide measurements were performed using Qlucore Omics Explorer 3.8 (Qlucore AB, Lund, Sweden). A positive threshold value of 1 was specified to enable a log_2_ transformation of abundance data for normalization, i.e., all abundance data values below the threshold will be replaced by 1 before the transformation. The transformed data were lastly used for statistical analysis, i.e., evaluation of differentially present proteins or peptides between two groups using a Student’s bilateral *t* test and assuming equal variance between groups. A *P* value better than 0.01 was used to filter differential candidates.

The confidence of modification sites is estimated by an AScore, which calculates an ambiguity score as −10 × log10(*p*). The *P* value indicates the likelihood that the peptide is matched by chance (AScore = 13 for a *P* value of 0.05).

### Immunofluorescence and microscopy

Fixation and indirect immunofluorescence of *C. elegans* embryos were performed essentially as described on subbing solution-coated slides (*87*). After freeze-crack and fixation with cold dehydrated methanol, slides were washed 3× 5 min, blocked for 1 hour in PBS + 2% BSA and incubated overnight at 4°C with primary antibodies diluted in PBS + 2% BSA. Working dilutions for the primary antibodies were 1/500 for rabbit lamin (LMN-1) and mouse Mab414 antibodies and 1/500 for rabbit NPP-19^NUP53^ antibodies (*39*). Slides were later incubated for 1 hour at room temperature with secondary antibodies, anti-Rabbit (1/1000), and anti-Mouse (1/1000) coupled to the Alexa 568 and 488 fluorophores, respectively. Next, embryos were mounted in VECTASHIELD Mounting Medium with DAPI (Vector). Fixed embryos were imaged using a spinning disc confocal microscope with 63×/numerical aperture (NA)1.4 objectives. Captured images were processed using ImageJ and Adobe Photoshop.

For cell cycle timing analysis in live specimens by differential interference contrast (DIC) microscopy, embryos were obtained by cutting open gravid hermaphrodites using two 21-gauge needles. Embryos were handled individually and mounted on a coverslip in 3 μl of M9 buffer. The coverslip was placed on a 3% agarose pad. DIC images were acquired by an Axiocam Hamamatsu ICc one camera (Hamamatsu Photonics, Bridgewater, NJ) mounted on a Zeiss AxioImager A1 microscope equipped with a Plan Neofluar 100×/1.3 NA objective (Carl Zeiss AG, Jena, Germany), and the acquisition system was controlled by Axiovision software (Carl Zeiss AG, Jena, Germany). Images were acquired at 10-s intervals.

Live imaging was performed at 23°C using a spinning disc confocal head (CSU-X1; Yokogawa Corporation of America) mounted on an Axio Observer Z1 inverted microscope (Zeiss) equipped with 491- and 561-nm lasers (OXXIUS 488 nm 150 mW; OXXIUS Laser 561 nm, 150 mW) and scientific complementary metal-oxide semiconductor PRIME 95 camera (Photometrics). Acquisition parameters were controlled by MetaMorph software (Molecular Devices). In all cases, a 63×, Plan-Apochromat 63×/1.4 Oil (Zeiss) lens was used, and approximately five *z* sections were collected at 1 μm and 10-s intervals. Captured images were processed using ImageJ and Adobe Photoshop.

### Reproducibility, quantification, and statistical analysis

All experiments presented in this manuscript have been repeated at least three times except the LC-MS/MS analysis performed once, using three independent embryonic extracts obtained from different worms cultures.

Tagged NPPs levels at the nuclear envelope were measured in two steps. First, we used the pixel classification workflow from Ilastik software (*88*), which assigns labels to pixels based on pixel features and user annotations. We classified pixels into two labels, one corresponding to the pixels at the NE and the other to the background. For every pixel of the image, Ilastik software estimated the probability that each pixel belongs to each label. The resulting probability maps for each image were then used for quantitative analysis. Once we had generated the pixel classification and the probability maps of each image, we then used the Fiji software to obtain the fluorescence intensity at the nuclear envelope by using the macro described in the Supplementary Materials. The macro consisted of thresholding, making a binary mask, creating a selection of the binary mask by making the region of interest of each frame, and measuring the integral density at the nuclear envelope. The average signal intensity of Tagged::NPPs at the NE 350 s before anaphase was arbitrarily defined as 1. The raw values were plotted only for the levels of NPP-1^NUP54^::GFP (Fig. 5B) and GFP::NPP-11^NUP62^ (Fig. 5C) at the nuclear envelope.

To determine when the NE becomes permeable (Fig. 3A), we quantified the fluorescence intensity of mCherry::Histone in the nucleoplasm starting at −200 s before anaphase onset (0 s) in the different strains analyzed Fig. 3B. We performed two separate steps of pixel classification. In the first step, we classified pixels corresponding to the chromosomes in one label and the pixels from the nucleoplasm and the cytoplasm to another label. In the second step, we classified the pixels of the total mCherry::Histone signal of both pronuclei to one label and the pixels corresponding to the background to another label. The resulting probability maps of each image were exported and used for quantitative analysis. We then used the Fiji software and applied the macro described in the Supplementary Materials to obtain the integral density of mCherry::Histone at the chromosomes (value A) and in the nucleus of both pronuclei (value B). We then obtained the integral density mCherry::Histone at the nucleoplasm (value C) by applying the formula (B − A = C) for each time point. The average signal intensity of mCherry::Histone at the nucleoplasm at 200 s before anaphase was arbitrarily defined as 1. The results are presented as means ± SEM.

To quantify PLK-1::sGFP levels at the NE in different RNAi conditions (Fig. 5A), we selected embryos at 200 s before anaphase onset and performed the quantification in three steps. First, we used the Fiji software to exclude the PLK-1::sGFP signal on chromosomes by masking the chromosomes with mCherry::Histone. Second, we used the freehand tool to select the two juxtaposed pronuclei and exclude the PLK-1::sGFP signal at the centrosomes. Third, we performed pixel classification with Ilastik software into two labels, one corresponding to pixels at the NE and the other to the background. Next, we used the Fiji software to obtain the fluorescence intensity at the nuclear envelope using the macro described in the Supplementary Materials. The raw integrated density values were plotted.

Statistical analyses were performed using ordinary one-way (Fig. 5A) or two-way (Fig. 5, B and C) analysis of variance (ANOVA) with multiple comparisons. *P < 0.05 was considered significant. All the graphs and calculations were performed using GraphPad Prism version 6.00 for Mac OS X, GraphPad Software (La Jolla, CA, USA).

## Supplementary Materials

### This PDF file includes:

Tables S1 to S3 Figs. S1 to S8 Data S1 and S2 Legends for tables S4 to S6 References

## Notes

View/request a protocol for this paper from *Bio-protocol*.

## References

[1] L. Champion, M. I. Linder, U. Kutay, Cellular reorganization during mitotic entry. Trends Cell Biol. 27, 26–41 (2017). 27528558 10.1016/j.tcb.2016.07.004

[2] P. De Magistris, W. Antonin, The dynamic nature of the nuclear envelope. Curr. Biol. 28, R487–R497 (2018). 29689232 10.1016/j.cub.2018.01.073

[3] B. Hampoelz, A. Andres-Pons, P. Kastritis, M. Beck, Structure and assembly of the nuclear pore complex. Annu. Rev. Biophys. 48, 515–536 (2019). 30943044 10.1146/annurev-biophys-052118-115308

[4] D. H. Lin, A. Hoelz, The structure of the nuclear pore complex (an update). Annu. Rev. Biochem. 88, 725–783 (2019). 30883195 10.1146/annurev-biochem-062917-011901 PMC6588426

[5] E. Dultz, M. Wojtynek, O. Medalia, E. Onischenko, The nuclear pore complex: Birth, life, and death of a cellular Behemoth. Cell 11, 1456 (2022). 10.3390/cells11091456 PMC9100368 35563762

[6] B. B. Hülsmann, A. A. Labokha, D. Görlich, The permeability of reconstituted nuclear pores provides direct evidence for the selective phase model. Cell 150, 738–751 (2012). 22901806 10.1016/j.cell.2012.07.019

[7] H. B. Schmidt, D. Görlich, Transport selectivity of nuclear pores, phase separation, and membraneless organelles. Trends Biochem. Sci. 41, 46–61 (2016). 26705895 10.1016/j.tibs.2015.11.001

[8] U. Kutay, R. Jühlen, W. Antonin, Mitotic disassembly and reassembly of nuclear pore complexes. Trends Cell Biol. 31, 1019–1033 (2021). 34294532 10.1016/j.tcb.2021.06.011

[9] E. Laurell, K. Beck, K. Krupina, G. Theerthagiri, B. Bodenmiller, P. Horvath, R. Aebersold, W. Antonin, U. Kutay, Phosphorylation of Nup98 by multiple kinases is crucial for NPC disassembly during mitotic entry. Cell 144, 539–550 (2011). 21335236 10.1016/j.cell.2011.01.012

[10] M. I. Linder, M. Köhler, P. Boersema, M. Weberruss, C. Wandke, J. Marino, C. Ashiono, P. Picotti, W. Antonin, U. Kutay, Mitotic disassembly of nuclear pore complexes involves CDK1- and PLK1-mediated phosphorylation of key interconnecting nucleoporins. Dev. Cell 43, 141–156.e7 (2017). 29065306 10.1016/j.devcel.2017.08.020 PMC5654724

[11] E. Dultz, E. Zanin, C. Wurzenberger, M. Braun, G. Rabut, L. Sironi, J. Ellenberg, Systematic kinetic analysis of mitotic dis- and reassembly of the nuclear pore in living cells. J. Cell Biol. 180, 857–865 (2008). 18316408 10.1083/jcb.200707026 PMC2265396

[12] H. Chug, S. Trakhanov, B. B. Hülsmann, T. Pleiner, D. Görlich, Crystal structure of the metazoan Nup62•Nup58•Nup54 nucleoporin complex. Science 350, 106–110 (2015). 26292704 10.1126/science.aac7420

[13] M. Heusel, M. Frank, M. Köhler, S. Amon, F. Frommelt, G. Rosenberger, I. Bludau, S. Aulakh, M. I. Linder, Y. Liu, B. C. Collins, M. Gstaiger, U. Kutay, R. Aebersold, A global screen for assembly state changes of the mitotic proteome by SEC-SWATH-MS. Cell Syst. 10, 133–155.e6 (2020). 32027860 10.1016/j.cels.2020.01.001 PMC7042714

[14] L. Pintard, B. Bowerman, Mitotic cell division in *Caenorhabditis elegans*. Genetics 211, 35–73 (2019). 30626640 10.1534/genetics.118.301367 PMC6325691

[15] O. Cohen-Fix, P. Askjaer, Cell biology of the *Caenorhabditis elegans* nucleus. Genetics 205, 25–59 (2017). 28049702 10.1534/genetics.116.197160 PMC5216270

[16] J. Liu, T. Rolef Ben-Shahar, D. Riemer, M. Treinin, P. Spann, K. Weber, A. Fire, Y. Gruenbaum, Essential roles for *Caenorhabditis elegans* lamin gene in nuclear organization, cell cycle progression, and spatial organization of nuclear pore complexes. Mol. Biol. Cell 11, 3937–3947 (2000). 11071918 10.1091/mbc.11.11.3937 PMC15048

[17] V. Galy, I. W. Mattaj, P. Askjaer, *Caenorhabditis elegans* nucleoporins Nup93 and Nup205 determine the limit of nuclear pore complex size exclusion in vivo. Mol. Biol. Cell 14, 5104–5115 (2003). 12937276 10.1091/mbc.E03-04-0237 PMC284812

[18] K. K. Lee, Y. Gruenbaum, P. Spann, J. Liu, K. L. Wilson, *C. elegans* nuclear envelope proteins emerin, MAN1, lamin, and nucleoporins reveal unique timing of nuclear envelope breakdown during mitosis. Mol. Biol. Cell 11, 3089–3099 (2000). 10982402 10.1091/mbc.11.9.3089 PMC14977

[19] V. Hachet, C. Busso, M. Toya, A. Sugimoto, P. Askjaer, P. Gonczy, The nucleoporin Nup205/NPP-3 is lost near centrosomes at mitotic onset and can modulate the timing of this process in *Caenorhabditis elegans* embryos. Mol. Biol. Cell 23, 3111–3121 (2012). 22740626 10.1091/mbc.E12-03-0204 PMC3418306

[20] L. Martino, S. Morchoisne-Bolhy, D. K. Cheerambathur, L. Van Hove, J. Dumont, N. Joly, A. Desai, V. Doye, L. Pintard, Channel nucleoporins recruit PLK-1 to nuclear pore complexes to direct nuclear envelope breakdown in *C. elegans*. Dev. Cell 43, 157–171.e7 (2017). 29065307 10.1016/j.devcel.2017.09.019 PMC8184135

[21] G. Velez-Aguilera, B. Ossareh-Nazari, L. Van Hove, N. Joly, L. Pintard, Cortical microtubule pulling forces contribute to the union of the parental genomes in the *Caenorhabditis elegans* zygote. eLife 11, e75382 (2022). 35259092 10.7554/eLife.75382 PMC8956289

[22] G. Velez-Aguilera, S. Nkombo Nkoula, B. Ossareh-Nazari, J. Link, D. Paouneskou, L. Van Hove, N. Joly, N. Tavernier, J. M. Verbavatz, V. Jantsch, L. Pintard, PLK-1 promotes the merger of the parental genome into a single nucleus by triggering lamina disassembly. eLife 9, e59510 (2020). 33030429 10.7554/eLife.59510 PMC7544505

[23] M. Rahman, I. Y. Chang, A. Harned, R. Maheshwari, K. Amoateng, K. Narayan, O. Cohen-Fix, *C. elegans* pronuclei fuse after fertilization through a novel membrane structure. J. Cell Biol. 219, e201909137 (2020). 31834351 10.1083/jcb.201909137 PMC7041684

[24] A. Audhya, A. Desai, K. Oegema, A role for Rab5 in structuring the endoplasmic reticulum. J. Cell Biol. 178, 43–56 (2007). 17591921 10.1083/jcb.200701139 PMC2064419

[25] V. Galy, W. Antonin, A. Jaedicke, M. Sachse, R. Santarella, U. Haselmann, I. Mattaj, A role for gp210 in mitotic nuclear-envelope breakdown. J. Cell Sci. 121, 317–328 (2008). 18216332 10.1242/jcs.022525

[26] N. Portier, A. Audhya, P. S. Maddox, R. A. Green, A. Dammermann, A. Desai, K. Oegema, A microtubule-independent role for centrosomes and Aurora A in nuclear envelope breakdown. Dev. Cell 12, 515–529 (2007). 17419991 10.1016/j.devcel.2007.01.019 PMC2973840

[27] D. Chase, C. Serafinas, N. Ashcroft, M. Kosinski, D. Longo, D. K. Ferris, A. Golden, The Polo-like kinase PLK-1 is required for nuclear envelope breakdown and the completion of meiosis in *Caenorhabditis elegans*. Genesis 26, 26–41 (2000). 10660671 10.1002/(sici)1526-968x(200001)26:1<26::aid-gene6>3.0.co;2-o

[28] M. M. Rahman, M. Munzig, K. Kaneshiro, B. Lee, S. Strome, T. Müller-Reichert, O. Cohen-Fix, C., *Caenorhabditis elegans* Polo-like kinase PLK-1 is required for merging parental genomes into a single nucleus. Mol. Biol. Cell 26, 4718–4735 (2015). 26490119 10.1091/mbc.E15-04-0244 PMC4678026

[29] A. E. Elia, L. C. Cantley, M. B. Yaffe, Proteomic screen finds pSer/pThr-binding domain localizing Plk1 to mitotic substrates. Science 299, 1228–1231 (2003). 12595692 10.1126/science.1079079

[30] A. E. Elia, P. Rellos, L. F. Haire, J. W. Chao, F. J. Ivins, K. Hoepker, D. Mohammad, L. C. Cantley, S. J. Smerdon, M. B. Yaffe, The molecular basis for phosphodependent substrate targeting and regulation of Plks by the Polo-box domain. Cell 115, 83–95 (2003). 14532005 10.1016/s0092-8674(03)00725-6

[31] M. Boxem, Z. Maliga, N. Klitgord, N. Li, I. Lemmens, M. Mana, L. de Lichtervelde, J. D. Mul, D. van de Peut, M. Devos, N. Simonis, M. A. Yildirim, M. Cokol, H. L. Kao, A. S. de Smet, H. Wang, A. L. Schlaitz, T. Hao, S. Milstein, C. Fan, M. Tipsword, K. Drew, M. Galli, K. Rhrissorrakrai, D. Drechsel, D. Koller, F. P. Roth, L. M. Iakoucheva, A. K. Dunker, R. Bonneau, K. C. Gunsalus, D. E. Hill, F. Piano, J. Tavernier, S. van den Heuvel, A. A. Hyman, M. Vidal, A protein domain-based interactome network for *C. elegans* early embryogenesis. Cell 134, 534–545 (2008). 18692475 10.1016/j.cell.2008.07.009 PMC2596478

[32] J. Link, D. Paouneskou, M. Velkova, A. Daryabeigi, T. Laos, S. Labella, C. Barroso, S. P. Piñol, A. Montoya, H. Kramer, A. Woglar, A. Baudrimont, S. M. Markert, C. Stigloher, E. Martinez-Perez, A. Dammermann, M. Alsheimer, M. Zetka, V. Jantsch, Transient and partial nuclear lamina disruption promotes chromosome movement in early meiotic prophase. Dev. Cell 45, 212–225.e7 (2018). 29689196 10.1016/j.devcel.2018.03.018 PMC5920155

[33] Y. C. Lussi, I. Hügi, E. Laurell, U. Kutay, B. Fahrenkrog, The nucleoporin Nup88 is interacting with nuclear lamin A. Mol. Biol. Cell 22, 1080–1090 (2011). 21289091 10.1091/mbc.E10-05-0463 PMC3069011

[34] T. Al-Haboubi, D. K. Shumaker, J. Köser, M. Wehnert, B. Fahrenkrog, Distinct association of the nuclear pore protein Nup153 with A- and B-type lamins. Nucleus 2, 500–509 (2011). 21983083 10.4161/nucl.2.5.17913

[35] L. A. Hawryluk-Gara, E. K. Shibuya, R. W. Wozniak, Vertebrate Nup53 interacts with the nuclear lamina and is required for the assembly of a Nup93-containing complex. Mol. Biol. Cell 16, 2382–2394 (2005). 15703211 10.1091/mbc.E04-10-0857 PMC1087243

[36] M. S. Mauro, G. Celma, V. Zimyanin, M. M. Magaj, K. H. Gibson, S. Redemann, S. Bahmanyar, Ndc1 drives nuclear pore complex assembly independent of membrane biogenesis to promote nuclear formation and growth. eLife 11, e75513 (2022). 35852146 10.7554/eLife.75513 PMC9296133

[37] R. Maheshwari, M. M. Rahman, S. Drey, M. Onyundo, G. Fabig, M. A. Q. Martinez, D. Q. Matus, T. Müller-Reichert, O. Cohen-Fix, A membrane reticulum, the centriculum, affects centrosome size and function in *Caenorhabditis elegans*. Curr. Biol. 33, 791–806.e7 (2023). 36693370 10.1016/j.cub.2022.12.059 PMC10023444

[38] J. P. Aris, G. Blobel, Yeast nuclear envelope proteins cross react with an antibody against mammalian pore complex proteins. J. Cell Biol. 108, 2059–2067 (1989). 2661560 10.1083/jcb.108.6.2059 PMC2115612

[39] E. Ródenas, E. P. Klerkx, C. Ayuso, A. Audhya, P. Askjaer, Early embryonic requirement for nucleoporin Nup35/NPP-19 in nuclear assembly. Dev. Biol. 327, 399–409 (2009). 19146848 10.1016/j.ydbio.2008.12.024

[40] L. A. Hawryluk-Gara, M. Platani, R. Santarella, R. W. Wozniak, I. W. Mattaj, Nup53 is required for nuclear envelope and nuclear pore complex assembly. Mol. Biol. Cell 19, 1753–1762 (2008). 18256286 10.1091/mbc.E07-08-0820 PMC2291426

[41] J. Fischer, R. Teimer, S. Amlacher, R. Kunze, E. Hurt, Linker Nups connect the nuclear pore complex inner ring with the outer ring and transport channel. Nat. Struct. Mol. Biol. 22, 774–781 (2015). 26344569 10.1038/nsmb.3084

[42] J. Kosinski, S. Mosalaganti, A. von Appen, R. Teimer, A. L. DiGuilio, W. Wan, K. H. Bui, W. J. Hagen, J. A. Briggs, J. S. Glavy, E. Hurt, M. Beck, Molecular architecture of the inner ring scaffold of the human nuclear pore complex. Science 352, 363–365 (2016). 27081072 10.1126/science.aaf0643 PMC8926079

[43] D. H. Lin, T. Stuwe, S. Schilbach, E. J. Rundlet, T. Perriches, G. Mobbs, Y. Fan, K. Thierbach, F. M. Huber, L. N. Collins, A. M. Davenport, Y. E. Jeon, A. Hoelz, Architecture of the symmetric core of the nuclear pore. Science 352, eaaf1015 (2016). 10.1126/science.aaf1015 PMC5207208 27081075

[44] S. Petrovic, D. Samanta, T. Perriches, C. J. Bley, K. Thierbach, B. Brown, S. Nie, G. W. Mobbs, T. A. Stevens, X. Liu, G. P. Tomaleri, L. Schaus, A. Hoelz, Architecture of the linker-scaffold in the nuclear pore. Science 376, eabm9798 (2022). 35679425 10.1126/science.abm9798 PMC9867570

[45] D. M. Rivers, S. Moreno, M. Abraham, J. Ahringer, PAR proteins direct asymmetry of the cell cycle regulators Polo-like kinase and Cdc25. J. Cell Biol. 180, 877–885 (2008). 18316412 10.1083/jcb.200710018 PMC2265398

[46] Y. Nishi, E. Rogers, S. M. Robertson, R. Lin, Polo kinases regulate *C. elegans* embryonic polarity via binding to DYRK2-primed MEX-5 and MEX-6. Development 135, 687–697 (2008). 18199581 10.1242/dev.013425

[47] Y. Budirahardja, P. Gonczy, PLK-1 asymmetry contributes to asynchronous cell division of *C. elegans* embryos. Development 135, 1303–1313 (2008). 18305005 10.1242/dev.019075

[48] N. Tavernier, A. Noatynska, C. Panbianco, L. Martino, L. Van Hove, F. Schwager, T. Léger, M. Gotta, L. Pintard, Cdk1 phosphorylates SPAT-1/Bora to trigger PLK-1 activation and drive mitotic entry in *C. elegans* embryos. J. Cell Biol. 208, 661–669 (2015). 25753036 10.1083/jcb.201408064 PMC4362466

[49] B. Han, K. R. Antkowiak, X. Fan, M. Rutigliano, S. P. Ryder, E. E. Griffin, Polo-like kinase couples cytoplasmic protein gradients in the *C. elegans* zygote. Curr. Biol. 28, 60–69.e8 (2018). 29276126 10.1016/j.cub.2017.11.048 PMC5763555

[50] A. Noatynska, C. Panbianco, M. Gotta, SPAT-1/Bora acts with Polo-like kinase 1 to regulate PAR polarity and cell cycle progression. Development 137, 3315–3325 (2010). 20823068 10.1242/dev.055293

[51] A. J. Kim, E. E. Griffin, PLK-1 regulation of asymmetric cell division in the early *C. elegans* embryo. Front Cell Dev. Biol. 8, 632253 (2020). 33553173 10.3389/fcell.2020.632253 PMC7859328

[52] C. J. Bley, S. Nie, G. W. Mobbs, S. Petrovic, A. T. Gres, X. Liu, S. Mukherjee, S. Harvey, F. M. Huber, D. H. Lin, B. Brown, A. W. Tang, E. J. Rundlet, A. R. Correia, S. Chen, S. G. Regmi, T. A. Stevens, C. A. Jette, M. Dasso, A. Patke, A. F. Palazzo, A. A. Kossiakoff, A. Hoelz, Architecture of the cytoplasmic face of the nuclear pore. Science 376, eabm9129 (2022). 35679405 10.1126/science.abm9129 PMC9348906

[53] K. Y. Cheng, E. D. Lowe, J. Sinclair, E. A. Nigg, L. N. Johnson, The crystal structure of the human Polo-like kinase-1 polo box domain and its phospho-peptide complex. EMBO J. 22, 5757–5768 (2003). 14592974 10.1093/emboj/cdg558 PMC275415

[54] J. E. Park, N. K. Soung, Y. Johmura, Y. H. Kang, C. Liao, K. H. Lee, C. H. Park, M. C. Nicklaus, K. S. Lee, Polo-box domain: A versatile mediator of Polo-like kinase function. Cell. Mol. Life Sci. 67, 1957–1970 (2010). 20148280 10.1007/s00018-010-0279-9 PMC2877763

[55] V. Archambault, G. Lépine, D. Kachaner, Understanding the Polo kinase machine. Oncogene 34, 4799–4807 (2015). 25619835 10.1038/onc.2014.451

[56] F. Gnad, J. Gunawardena, M. Mann, PHOSIDA 2011: The posttranslational modification database. Nucleic Acids Res. 39, D253–D260 (2011). 21081558 10.1093/nar/gkq1159 PMC3013726

[57] C. Franz, R. Walczak, S. Yavuz, R. Santarella, M. Gentzel, P. Askjaer, V. Galy, M. Hetzer, I. W. Mattaj, W. Antonin, MEL-28/ELYS is required for the recruitment of nucleoporins to chromatin and postmitotic nuclear pore complex assembly. EMBO Rep. 8, 165–172 (2007). 17235358 10.1038/sj.embor.7400889 PMC1796766

[58] V. Galy, P. Askjaer, C. Franz, C. López-Iglesias, I. W. Mattaj, MEL-28, a novel nuclear-envelope and kinetochore protein essential for zygotic nuclear-envelope assembly in *C. elegans*. Curr. Biol. 16, 1748–1756 (2006). 16950114 10.1016/j.cub.2006.06.067

[59] G. Gómez-Saldivar, A. Fernandez, Y. Hirano, M. Mauro, A. Lai, C. Ayuso, T. Haraguchi, Y. Hiraoka, F. Piano, P. Askjaer, Identification of conserved MEL-28/ELYS domains with essential roles in nuclear assembly and chromosome segregation. PLOS Genet. 12, e1006131 (2016). 27341616 10.1371/journal.pgen.1006131 PMC4920428

[60] B. Vollmer, A. Schooley, R. Sachdev, N. Eisenhardt, A. M. Schneider, C. Sieverding, J. Madlung, U. Gerken, B. Macek, W. Antonin, Dimerization and direct membrane interaction of Nup53 contribute to nuclear pore complex assembly. EMBO J. 31, 4072–4084 (2012). 22960634 10.1038/emboj.2012.256 PMC3474928

[61] S. Otsuka, J. Ellenberg, Mechanisms of nuclear pore complex assembly–two different ways of building one molecular machine. FEBS Lett. 592, 475–488 (2018). 29119545 10.1002/1873-3468.12905 PMC6220763

[62] S. Otsuka, J. O. B. Tempkin, W. Zhang, A. Z. Politi, A. Rybina, M. J. Hossain, M. Kueblbeck, A. Callegari, B. Koch, N. R. Morero, A. Sali, J. Ellenberg, A quantitative map of nuclear pore assembly reveals two distinct mechanisms. Nature 613, 575–581 (2023). 36599981 10.1038/s41586-022-05528-w PMC9849139

[63] M. E. Hase, V. C. Cordes, Direct interaction with nup153 mediates binding of Tpr to the periphery of the nuclear pore complex. Mol. Biol. Cell 14, 1923–1940 (2003). 12802065 10.1091/mbc.E02-09-0620 PMC165087

[64] S. G. Regmi, H. Lee, R. Kaufhold, B. Fichtman, S. Chen, V. Aksenova, E. Turcotte, A. Harel, A. Arnaoutov, M. Dasso, The nuclear pore complex consists of two independent scaffolds. bioRxiv 2020.11.13.381947; 10.1101/2020.11.13.381947 (2020).

[65] N. Belgareh, G. Rabut, S. W. Baï, M. van Overbeek, J. Beaudouin, N. Daigle, O. V. Zatsepina, F. Pasteau, V. Labas, M. Fromont-Racine, J. Ellenberg, V. Doye, An evolutionarily conserved NPC subcomplex, which redistributes in part to kinetochores in mammalian cells. J. Cell Biol. 154, 1147–1160 (2001). 11564755 10.1083/jcb.200101081 PMC2150808

[66] E. Ródenas, C. González-Aguilera, C. Ayuso, P. Askjaer, Dissection of the NUP107 nuclear pore subcomplex reveals a novel interaction with spindle assembly checkpoint protein MAD1 in *Caenorhabditis elegans*. Mol. Biol. Cell 23, 930–944 (2012). 22238360 10.1091/mbc.E11-11-0927 PMC3290650

[67] M. Zuccolo, A. Alves, V. Galy, S. Bolhy, E. Formstecher, V. Racine, J. B. Sibarita, T. Fukagawa, R. Shiekhattar, T. Yen, V. Doye, The human Nup107-160 nuclear pore subcomplex contributes to proper kinetochore functions. EMBO J. 26, 1853–1864 (2007). 17363900 10.1038/sj.emboj.7601642 PMC1847668

[68] P. Bensidoun, T. Reiter, B. Montpetit, D. Zenklusen, M. Oeffinger, Nuclear mRNA metabolism drives selective basket assembly on a subset of nuclear pore complexes in budding yeast. Mol. Cell 82, 3856–3871.e6 (2022). 36220102 10.1016/j.molcel.2022.09.019 PMC10300651

[69] D. Kachaner, D. Garrido, H. Mehsen, K. Normandin, H. Lavoie, V. Archambault, Coupling of Polo kinase activation to nuclear localization by a bifunctional NLS is required during mitotic entry. Nat. Commun. 8, 1701 (2017). 29167465 10.1038/s41467-017-01876-8 PMC5700101

[70] L. Pintard, V. Archambault, A unified view of spatio-temporal control of mitotic entry: Polo kinase as the key. Open Biol. 8, 180114 (2018). 30135239 10.1098/rsob.180114 PMC6119860

[71] T. Stuwe, C. J. Bley, K. Thierbach, S. Petrovic, S. Schilbach, D. J. Mayo, T. Perriches, E. J. Rundlet, Y. E. Jeon, L. N. Collins, F. M. Huber, D. H. Lin, M. Paduch, A. Koide, V. Lu, J. Fischer, E. Hurt, S. Koide, A. A. Kossiakoff, A. Hoelz, Architecture of the fungal nuclear pore inner ring complex. Science 350, 56–64 (2015). 26316600 10.1126/science.aac9176 PMC4826903

[72] J. Mansfeld, S. Güttinger, L. A. Hawryluk-Gara, N. Panté, M. Mall, V. Galy, U. Haselmann, P. Mühlhäusser, R. W. Wozniak, I. W. Mattaj, U. Kutay, W. Antonin, The conserved transmembrane nucleoporin NDC1 is required for nuclear pore complex assembly in vertebrate cells. Mol. Cell 22, 93–103 (2006). 16600873 10.1016/j.molcel.2006.02.015

[73] K. Zhu, Z. Shan, L. Zhang, W. Wen, Phospho-pon binding-mediated fine-tuning of Plk1 activity. Structure 24, 1110–1119 (2016). 27238966 10.1016/j.str.2016.04.012

[74] P. Singh, M. E. Pesenti, S. Maffini, S. Carmignani, M. Hedtfeld, A. Petrovic, A. Srinivasamani, T. Bange, A. Musacchio, BUB1 and CENP-U, Primed by CDK1, are the main PLK1 kinetochore receptors in mitosis. Mol. Cell 81, 67–87.e9 (2021). 33248027 10.1016/j.molcel.2020.10.040 PMC7837267

[75] B. J. Blus, J. Koh, A. Krolak, H. S. Seo, E. Coutavas, G. Blobel, Allosteric modulation of nucleoporin assemblies by intrinsically disordered regions. Sci. Adv. 5, eaax1836 (2019). 31807700 10.1126/sciadv.aax1836 PMC6881172

[76] S. Brenner, The genetics of *Caenorhabditis elegans*. Genetics 77, 71–94 (1974). 4366476 10.1093/genetics/77.1.71 PMC1213120

[77] R. S. Kamath, M. Martinez-Campos, P. Zipperlen, A. G. Fraser, J. Ahringer, Effectiveness of specific RNA-mediated interference through ingested double-stranded RNA in *Caenorhabditis elegans*. Genome Biol. 2, RESEARCH0002 (2001). 11178279 10.1186/gb-2000-2-1-research0002 PMC17598

[78] A. E. Friedland, Y. B. Tzur, K. M. Esvelt, M. P. Colaiácovo, G. M. Church, J. A. Calarco, Heritable genome editing in *C. elegans* via a CRISPR-Cas9 system. Nat. Methods 10, 741–743 (2013). 23817069 10.1038/nmeth.2532 PMC3822328

[79] J. A. Arribere, R. T. Bell, B. X. Fu, K. L. Artiles, P. S. Hartman, A. Z. Fire, Efficient marker-free recovery of custom genetic modifications with CRISPR/Cas9 in *Caenorhabditis elegans*. Genetics 198, 837–846 (2014). 25161212 10.1534/genetics.114.169730 PMC4224173

[80] M. L. Schwartz, E. M. Jorgensen, SapTrap, a toolkit for high-throughput CRISPR/Cas9 gene modification in *Caenorhabditis elegans*. Genetics 202, 1277–1288 (2016). 26837755 10.1534/genetics.115.184275 PMC4905529

[81] C. Muñoz-Jiménez, C. Ayuso, A. Dobrzynska, A. Torres-Mendéz, P. C. Ruiz, P. Askjaer, An efficient FLP-based toolkit for spatiotemporal control of gene expression in *Caenorhabditis elegans*. Genetics 206, 1763–1778 (2017). 28646043 10.1534/genetics.117.201012 PMC5560786

[82] J. Vicencio, C. Martínez-Fernández, X. Serrat, J. Cerón, Efficient generation of endogenous fluorescent reporters by nested CRISPR in *Caenorhabditis elegans*. Genetics 211, 1143–1154 (2019). 30696716 10.1534/genetics.119.301965 PMC6456308

[83] G. A. Dokshin, K. S. Ghanta, K. M. Piscopo, C. C. Mello, Robust genome editing with short single-stranded and long, partially single-stranded DNA donors in *Caenorhabditis elegans*. Genetics 210, 781–787 (2018). 30213854 10.1534/genetics.118.301532 PMC6218216

[84] T. Stiernagle, *Maintenance of C. elegans*. (Oxford Univ. Press, 2006), pp. 1–11. 10.1895/wormbook.1.101.1 PMC4781397 18050451

[85] E. Zanin, J. Dumont, R. Gassmann, I. Cheeseman, P. Maddox, S. Bahmanyar, A. Carvalho, S. Niessen, J. R. Yates, K. Oegema, A. Desai, Affinity purification of protein complexes in *C. elegans*. Methods Cell Biol. 106, 289–322 (2011). 22118282 10.1016/B978-0-12-544172-8.00011-6 PMC3319706

[86] F. Meier, S. Beck, N. Grassl, M. Lubeck, M. A. Park, O. Raether, M. Mann, Parallel accumulation-serial fragmentation (PASEF): Multiplying sequencing speed and sensitivity by synchronized scans in a trapped ion mobility device. J. Proteome Res. 14, 5378–5387 (2015). 26538118 10.1021/acs.jproteome.5b00932

[87] N. Joly, E. Beaumale, L. Van Hove, L. Martino, L. Pintard, Phosphorylation of the microtubule-severing AAA^+^ enzyme Katanin regulates *C. elegans* embryo development. J. Cell Biol. 219, e201912037 (2020). 32412594 10.1083/jcb.201912037 PMC7265321

[88] S. Berg, D. Kutra, T. Kroeger, C. N. Straehle, B. X. Kausler, C. Haubold, M. Schiegg, J. Ales, T. Beier, M. Rudy, K. Eren, J. I. Cervantes, B. Xu, F. Beuttenmueller, A. Wolny, C. Zhang, U. Koethe, F. A. Hamprecht, A. Kreshuk, ilastik: Interactive machine learning for (bio)image analysis. Nat. Methods 16, 1226–1232 (2019). 31570887 10.1038/s41592-019-0582-9

[89] E. Voronina, G. Seydoux, The *C. elegans* homolog of nucleoporin Nup98 is required for the integrity and function of germline P granules. Development 137, 1441–1450 (2010). 20335358 10.1242/dev.047654 PMC2853846

[90] L. Thomas, B. Taleb Ismail, P. Askjaer, G. Seydoux, Nucleoporin foci are stress-sensitive condensates dispensable for *C. elegans* nuclear pore assembly. *EMBO J.*, e112987 (2023). 10.15252/embj.2022112987 PMC10308366 37254647

[91] M. Sarov, J. I. Murray, K. Schanze, A. Pozniakovski, W. Niu, K. Angermann, S. Hasse, M. Rupprecht, E. Vinis, M. Tinney, E. Preston, A. Zinke, S. Enst, T. Teichgraber, J. Janette, K. Reis, S. Janosch, S. Schloissnig, R. K. Ejsmont, C. Slightam, X. Xu, S. K. Kim, V. Reinke, A. F. Stewart, M. Snyder, R. H. Waterston, A. A. Hyman, A genome-scale resource for in vivo tag-based protein function exploration in *C. elegans*. Cell 150, 855–866 (2012). 22901814 10.1016/j.cell.2012.08.001 PMC3979301

[92] N. Hattersley, D. Cheerambathur, M. Moyle, M. Stefanutti, A. Richardson, K. Y. Lee, J. Dumont, K. Oegema, A. Desai, A nucleoporin docks protein phosphatase 1 to direct meiotic chromosome segregation and nuclear assembly. Dev. Cell 38, 463–477 (2016). 27623381 10.1016/j.devcel.2016.08.006 PMC5094371

[93] R. S. Kamath, A. G. Fraser, Y. Dong, G. Poulin, R. Durbin, M. Gotta, A. Kanapin, N. Le Bot, S. Moreno, M. Sohrmann, D. P. Welchman, P. Zipperlen, J. Ahringer, Systematic functional analysis of the *Caenorhabditis elegans* genome using RNAi. Nature 421, 231–237 (2003). 12529635 10.1038/nature01278

[94] M. Kumar, S. Michael, J. Alvarado-Valverde, B. Mészáros, H. Sámano-Sánchez, A. Zeke, L. Dobson, T. Lazar, M. Örd, A. Nagpal, N. Farahi, M. Käser, R. Kraleti, N. E. Davey, R. Pancsa, L. B. Chemes, T. J. Gibson, The eukaryotic linear motif resource: 2022 release. Nucleic Acids Res. 50, D497–D508 (2022). 34718738 10.1093/nar/gkab975 PMC8728146

[95] A. M. Waterhouse, J. B. Procter, D. M. Martin, M. Clamp, G. J. Barton, Jalview version 2—A multiple sequence alignment editor and analysis workbench. Bioinformatics 25, 1189–1191 (2009). 19151095 10.1093/bioinformatics/btp033 PMC2672624

[96] C. A. Schneider, W. S. Rasband, K. W. Eliceiri, NIH Image to ImageJ: 25 years of image analysis. Nat. Methods 9, 671–675 (2012). 22930834 10.1038/nmeth.2089 PMC5554542

