# Supplementary material for: Mechanisms of nuclear pore complex disassembly by the mitotic Polo-like kinase 1 (PLK-1) in C. elegans embryos
Source: Sci Adv. 2023 Jul 19;9(29):eadf7826. doi: 10.1126/sciadv.adf7826 (PMC10355831; doi:10.1126/sciadv.adf7826)
Supplement: Supplementary file 1 — Tables S1 to S3 Figs. S1 to S8 Data S1 and S2 Legends for tables S4 to S6 References [file sciadv.adf7826_sm.pdf]

Science Advances
AAAS

Supplementary Materials for

Mechanisms of nuclear pore complex disassembly by the mitotic Polo-like kinase 1 (PLK-1) in *C.* elegans embryos

Sylvia Nkombo Nkoula *et al.*

Corresponding author: Lionel Pintard, lionel.pintard@ijm.fr

Sci. Adv. **9**, eadf7826 (2023)
DOI: 10.1126/sciadv.adf7826

The PDF file includes:

- Tables S1 to S3
- Figs. S1 to S8
- Data S1 and S2
- Legends for tables S4 to S6
- References

Other Supplementary Material for this manuscript includes the following:

- Tables S4 to S6

Table S1: List of strains used in this study:

Name/alias	Description	Genotype	Source	Infos
N2		*C. elegans* N2 Bristol	CGC	
HT1593		*unc-119(ed3)III*	CGC	
OCF3/WLP584	NPP-1::GFP	*unc-119(ed3) III; ltIs37 IV; jjIs1092. jjIs1092 [(pNUT1) npp-1::GFP + unc-119(+)]. ltIs37 [pie-1p::mCherry::his-58 + unc-119(+)]IV.*	Cohen-fix lab	
BN69	GFP::NPP-5	*npp-5(tm3039)/mIn1 [mIs14 dpy-10(e128)] II; pie-1p::GFP::npp-5 (bqls51) pie-1p::mCherry::his-58 (ltIs37) IV*	(66)	
PHX1469/WLP799	sGFP::NPP-7	*npp-7(syb1469)I*	This study, SunyBiotech	
PHX1475/WLP800	mcherry::NPP-8	*npp-8(syb1475)IV*	This study, SunyBiotech	
JH2184	GFP::NPP-9	*unc-119(ed3) III; axIs1595. axIs1595 [pie-1p::GFP::npp-9(orf)::npp-9 3'UTR + unc-119(+)]*	(89)	
JH3867	Neongreen::NPP-10, mCherry::Histone	*mNeonGreen::npp-10 (ax4538) III; lmn-1p::mCherry::his-58::pie-1utr(bqSi189)II*	(90)	
JH3872	NPP-14::OLLAS	*npp-14::OLLAS (ax4548)I*	(90)	
TH239/WLP613	GFP::NPP-11	*unc-119(ed3)III::ddls77[npp-11::TY1::EGFP:: 3xFLAG + unc-119(+)]*	(91)	
PHX1526/WLP805	GFP::NPP-13	*npp-13(syb1526)I*	This study, SunyBiotech	
BN189	mcherry::Histone	*bqSi189[pBN13(unc-119(+) lmn-1p::mCherry::his-58)]II*	(59)	
BN424	NPP-21::GFP	*dpy-10(cn64) npp-21(bq1[npp-21::gfp])II*	This study	Made by injection of N2
BN678	NPP-21::GFP, mcherry::Histone	*dpy-10(cn64) npp-21(bq1[npp-21::gfp]) bqSi189[pBN13(unc-119(+) lmn-1p::mCherry::his-58)]II*	This study	Made by crossing BN189 and BN424
BN1062	NPP-21::GFP, mcherry::Histone	*npp-21(bq1[npp-21::gfp]) bqSi189[pBN13(unc-119(+) lmn-1p::mCherry::his-58)]II*	This study	Made by crossing N2 and BN678
BN1015/WLP824	GFP::NPP-19	*npp-19(bq29[GFP::npp-19])II*	This study	
BN1018/WLP894	GFP::NPP-19, mCherry::Histone	*npp-19(bq29[G>F>P::npp-19]) bqSi189[pBN13(unc-119(+) lmn-1p::mCherry::his-58)]*	This study	Made by injection of HT1593

PHX1474/WLP801	mCherry::NPP-22	*npp-22(syb1474)V*	This study	SunyBiotech
BN738	GFP::NPP-24	*npp-24(bq14[GFP-Frt-KO>unc-119(+)>npp-24])II; unc-119(ed3)III*	This study	Made by injection of HT1593
BN739	GFP::NPP-24	*npp-24(bq15[G>F>P::npp-24]) II; unc-119(ed3) III*	This study	Made by injection of BN738 with Cre expression plasmid
BN740	GFP::NPP-24, mcherry::Histone	*npp-24(bq15[G>F>P::npp-24]) bqSi189[pBN13(unc-119(+) lmn-1p::mCherry::his-58)]II*	This study	Made by crossing BN189 and BN739
BN452	GFP::MEL-28, mcherry::Histone	*bqSi189 [lmn-1p::mCherry::his-58 + unc-119(+)] II. bq5[GFP::mel-28]) III.*	(*59*)	
WLP820	GFP::NPP-7, mcherry::Histone	*npp-7(syb1469)I; mcherry:his58 II*	This study	
UV2059/ WLP758	LMN-1 8A	*lmn-1(jf140[S21,22,24,32,397,398, 403,405A]) I/hT2 [bli-4(e937) let-?(q782) qIs48*] (I;III)	(*22*)	
WLP893	LMN-1 8A, GFP::NPP-19	*lmn-1(jf140[S21,22,24,32,397,398, 403,405A]) I/hT2 [bli-4(e937) let-?(q782) qIs48*] (I;III); *npp-19(bq29[G>F>P::npp-19])II*	This study	Made by crossing WLP758 and WLP894
WLP922	LMN-1 8A, GFP::NPP-19, mCherry::Histone	*lmn-1(jf140[S21,22,24,32,397,398, 403,405A]) I/hT2 [bli-4(e937) let-?(q782) qIs48*] (I;III); *npp-19(bq29[G>F>P::npp-19]) bqSi189[pBN13(unc-119(+) lmn-1p::mCherry::his-58)]*	This study	Made by crossing WLP758 and WLP894
WLP926	LMN-1 8A, mCherry::NPP-22	*lmn-1(jf140[S21,22,24,32,397,398, 403,405A]) I/hT2 [bli-4(e937) let-?(q782) qIs48*] (I;III) *npp-22(syb1474)V*	This study	Made by crossing WLP758 and WLP801
PHX1534	NPP-19 10A	*npp-19 10A(syb1524)II*	This study	SunyBiotech
WLP806	NPP-19 10A	*npp-19 10A(syb1524)II*	This study	Made by crossing PHX1534 with N2
WLP809	NPP-19 10A	*npp-19 10A(syb1524)II*	This study	Made by crossing PHX1534 with N2

WLP844	NPP-19 10A, sGFP::NPP-13	*npp-13(syb1526)I; npp-19 10A(syb1524)II*	This study	Made by crossing WLP805 and WLP821
WLP821	NPP-19 10A, mcherry::NPP-8	*npp-19 10A(syb1524)II; npp-8(syb1475)IV*	This study	Made by crossing WLP800 and WLP809
WLP1107	NPP-19 10A, GFP::NPP-11	*npp-19 10A(syb1524)II; unc-119(ed3)III::ddls77[npp-11::TY1::EGFP:: 3xFLAG + unc-119(+)]*	This study	Made by crossing WLP584 and WLP809
WLP1106	NPP-19 10A, NPP-1::GFP	*npp-19 10A(syb1524)II; unc-119(ed3) III; ltIs37 IV; jjIs1092. jjIs1092 [(pNUT1) npp-1::GFP + unc-119(+)]. ltIs37 [pie-1p::mCherry::his-58 + unc-119(+)] IV.*	This study	Made by crossing WLP613 and WLP809
OD2425	PLK-1::sGFP	*plk-1(lt18)* ([*plk-1::sgfp*] *loxp)*III	(*20*)	
WLP552	PLK-1::sGFP, mCherry::Histone	*plk-1(lt18)* ([*plk-1::sgfp*] *loxp)*III, *Pmex5 - mcherry-his-11- 3'UTR tbb-2* II	(*20*)	
WLP1160	*npp-19* 10A, PLK-1::sGFP	*npp-19* 10A*(syb1524)II; plk-1(lt18) ([plk-1::sgfp] loxp)III*	This study	WLP552 and WLP1006
OD999	GFP::NPP-18, mCherry::Histone	*unc- 119(ed3)III; ltSi245[pNH42;Pnpp-18::GFP- npp- 18; cb- unc-119(+)]II; ltIs37[pAA64; pie-1/mCherry::his- 58; unc-119 (+)] IV*	*(92)*	
OD1496	GFP::NPP-6, mCherry::Histone	*unc- 119(ed3)III; ltSi464[pNH103;Pmex-5::npp- 6::GFP::tbb-2:3'UTR; cbunc- 119(+)]I; ltIs37[pAA64;pie-1/mCherry::his- 58; unc- 119 (+)] IV*	(*92*)	

Table S2: Chemical and reagents used in this study:

Reagent type (species) or resource	Designation	Source or reference	Identifiers
Antibody	Plk1 (human) (Mouse monoclonal)	Merck Millipore	Cat#05–844 RRID:AB_310836
Antibody	GST (Rabbit polyclonal)	This study	L. Pintard Lab (Home made)
Antibody	Anti-Mouse IgG (Fab specific) -Peroxidase antibody (produced in goat)	Sigma	Cat#A9917 RRID:AB_258476
Antibody	Anti-Rabbit IgG (whole molecule) -Peroxidase antibody (Produced in goat)	Sigma	Cat#A0545 RRID:AB_257896
Antibody	Anti-Rabbit IgG (H+L) Cross-Adsorbed Secondary Antibody, Alexa Fluor 568 (Produced in goat) Fluor 568	Invitrogen	Cat#A-11011 RRID:AB_143157
Antibody	Anti-Mouse IgG (H+L) Cross-Adsorbed Secondary Antibody, Alexa Fluor 488 (Produced in goat)	Invitrogen	Cat#A-11001 RRID:AB_2534069
Antibody	OLLAS (Rat monoclonal)	Novus	Cat#NBP1-067113
Chemical compound, drug	Coomassie R250	Sigma	Cat#B014925G
Chemical compound, drug	Ponceau Red	Sigma	Cat#A1405
Chemical compound, drug	VECTASHIELD Mounting Medium with DAPI	Eurobio	Cat#H-1200
Chemical compound, drug	IPTG	Euromedex	Cat#EU0008-B
Chemical compound, drug	GLUCOSE	Sigma	Cat#G8270
Chemical compound, drug	Adenosine TriPhosphate (ATP)	Sigma	Cat#A2383
Chemical compound, drug	Imidazole	Sigma	Cat#I202
Chemical compound, drug	Glutathione	Sigma	Cat#G4251
Chemical compound, drug	HiTrap Chelating HP 5 × 1 mL	GE Healthcare	Cat#17-0408-01
Chemical compound, drug	MBPTrap HP 5 x 1mL	GE Healthcare	Cat#28-9187-78
Chemical compound, drug	Glutathion Sepharose 4B Fast Flow	GE Healthcare	Cat#17-0756-01
Chemical compound, drug	Pfu	Promega	Cat#M7741

Chemical compound, drug	DpnI	Biolabs	Cat#R0176S
Chemical compound, drug	NdeI	ThermoScientific	Cat# 10349709
Commercial assay or kit	ECL reagent	Millipore	Cat#WBKLS0500
Commercial assay or kit	BP Clonase II Enzyme Mix (Gateway cloning)	Invitrogen	Cat#11789–020
Commercial assay or kit	LR Clonase II Enzyme Mix (Gateway cloning)	Invitrogen	Cat#11791–020
Recombinant protein	Human Cyclin B-Cdk1 kinase	New England Biolabs	Cat#P6020L
Recombinant DNA reagent	L4440 (RNAi Feeding vector)	(*93*)	N/A
Recombinant DNA reagent	*plk-1* cloned into L4440	(*93*)	Arhinger Library
Recombinant DNA reagent	*npp-1* cloned into L4440	(*93*)	Arhinger Library
Recombinant DNA reagent	*npp-10* cloned into L4440	(*93*)	Arhinger Library
Recombinant DNA reagent	*npp-14* cloned into L4440	(*93*)	Arhinger Library
Recombinant DNA reagent	*npp-19* cloned into L4440	(*93*)	Arhinger Library
Recombinant DNA reagent	Gal4 pDEST DB-PLK-1 PBD H542A, K544M	(*50*)	pMG538
Recombinant DNA reagent	pGEX-4T (GST)	GE Healthcare	Cat#GE28-9545-49
Recombinant DNA reagent	pFasTBAC Hta PLK-1 *C. elegans*	(*48*)	pLP871
Recombinant DNA reagent	pGEX-6p1 GST-PLK-1 PBD *H. s*	Gift I. Sumara	N/A
Recombinant DNA reagent	pGEX-6p1 GST-PLK-1 PBD H538A/K540M *H. s*	Gift I. Sumara	N/A
Recombinant DNA reagent	pDONR201	ThermoFisher	N/A
Recombinant DNA reagent	pDONR201 NPP-19 (aa1-378)	This study	pLP1675
Recombinant DNA reagent	pDONR201 NPP-10N (aa513-821)	This study	pLP2630
Recombinant DNA reagent	pDEST17 His-NPP-19 (aa1-301)	This study	pLP2352
Recombinant DNA reagent	pDEST17 His-NPP-19 3A (aa1-301)	This study	pLP2361
Recombinant DNA reagent	pDEST17 His-NPP-19 8A (aa1-301)	This study	pLP2353
Recombinant DNA reagent	pDEST15	ThermoFisher	Cat#11802014
Recombinant DNA reagent	pDEST 15 GST-NPP-19 (aa1-378)	This study	pLP1858
Recombinant DNA reagent	pDEST 15 GST-NPP-19 3A (aa1-378)	This study	pLP2035
Recombinant DNA reagent	pDEST 15 GST-NPP-19 10A (aa1-378)	This study	pLP2143

Recombinant DNA reagent	pMAL Gateway	Thermofisher	pLP354
Recombinant DNA reagent	pDEST MBP-NPP-10N (aa513-821)	This study	pLP2633
Recombinant DNA reagent	pDEST MBP-NPP-10N 3A (aa513-821)	This study	pLP2682
Recombinant DNA reagent	pDEST MBP-NPP-10N 8A (aa513-821)	This study	pLP2637
Sequence-based reagent	Primers for cloning and site-directed mutagenesis (see oligonucleotide sequences table source)	This study	N/A
Software, algorithm	Clustal Omega	EMBL-EBI	https://www.ebi.ac.uk/Tools/msa/clustalo/
Software, algorithm	The eukaryotic linear resource for functional sites in proteins	(*94*)	http://elm.eu.org/
Software, algorithm	Jalview	(*95*)	https://www.jalview.org/
Software, algorithm	Adobe Illustrator CS6	Adobe	https://www.adobe.com/products/illustrator.html
Software, algorithm	Adobe Photoshop CS4	Adobe	https://www.adobe.com/products/photoshop.html
Software, algorithm	Affinity Designer	Affinity	https://affinity.serif.com/en-gb/
Software, algorithm	Image J	NIH; (*96*)	https://imagej.nih.gov/ij/
Software, algorithm	ZEN	Zeiss	https://www.zeiss.com/microscopy/int/products/microscope-software/zen.html
Software, algorithm	PRISM	Graphpad	https://www.graphpad.com/
Software, algorithm	Metamorph	Molecular Devices	https://www.metamorph.com/
Software, algorithm	Proteome Discoverer 2.2	Thermo Scientific	https://www.thermofisher.com/store/products/OPTON-30945#/OPTON-30945

Table S3: List of primers used in this study:

Name	Description	Sequence
OLP2993	for to amplify NPP-10 aa513 for Gateway entry	GGGGACAAGTTTGTACAAAAAAGCAGGCTTCATGAAGTCATTGAAGTCGCAG
OLP2994	rev to amplify NPP-19 aa821 for Gateway entry	GGGGACCACTTTGTACAAGAAAGCTGGGTCCTACTCGACTCGTCCTGGCCAG
OLP3021	for Oligo to mutate S544A in NPP-10	CCAGAAATTGATGGGAAGTCTGCGCCGGCTAGTACTCAACGACAGC
OLP3022	rev oligo to mutate S544A in NPP-10	GCTGTCGTTGAGTACTAGCCGGCGCAGACTTCCCATCAATTTCTGG
OLP3023	for oligo to mutate T708A in NPP-10	CAAGACGTTGTCACTTCCGCTCCAGCAGTTGACCCCG
OLP3024	rev oligo to mutate T708A in NPP-10	CGGGGTCAACTGCTGGAGCGGAAGTGACAACGTCTTG
OLP3025	for oligo to mutate T758A in NPP-10	CAGCAGCATCTGTTGTGTCGGCGCCTTCTGAAGAAACTG
OLP3025	rev oligo to mutate T758A in NPP-10	CAGTTTCTTCAGAAGGCGCCGACACAACAGATGCTGCTG
OLP1290	for to amplify NPP-19 aa1 for Gateway entry	GGGGACAAGTTTGTACAAAAAAGCAGGCTTCATGTTCTCGCATCTTAACC
OLP1291	rev to amplify NPP-19 aa378 for Gateway entry	GGGGACCACTTTGTACAAGAAAGCTGGGTCTTAGTTGAGTCCGATCGTGTTCC
OLP1528	rev to amplify NPP-19 aa301 for	GGGGACCACTTTGTACAAGAAAGCTGGGTCTTATGAGTTATACATGGAAGCAGATCGGT

	Gateway entry	
OLP1800	for to mutate T34A in NPP-19	ACCAGTTGAACAGTCTGCGCCGGCACTTCTTTTC
OLP1801	rev to mutate T34A in NPP-19	GAAAAGAAGTGCCGGCGCAGACTGTTCAACTGGT
OLP1802	for to mutate T168A and T172A in NPP-19	TTCACTGCCCGCAGCGCCCCACTCTCCGCACCAATCACTCAAAGGG
OLP1803	rev to mutate T168A and T172A in NPP-19	CCCTTTGAGTGATTGGTGCGGAGAGTGGGGCGCTGCGGGCAGTGAA
OLP2275	for (tm2886 internal wormbase)	CATCCGGAAGGCATAACTCA
OLP2276	rev (tm2886 internal wormbase)	CATGGAAGCAGATCGGTTTG

Supplementary Figures:

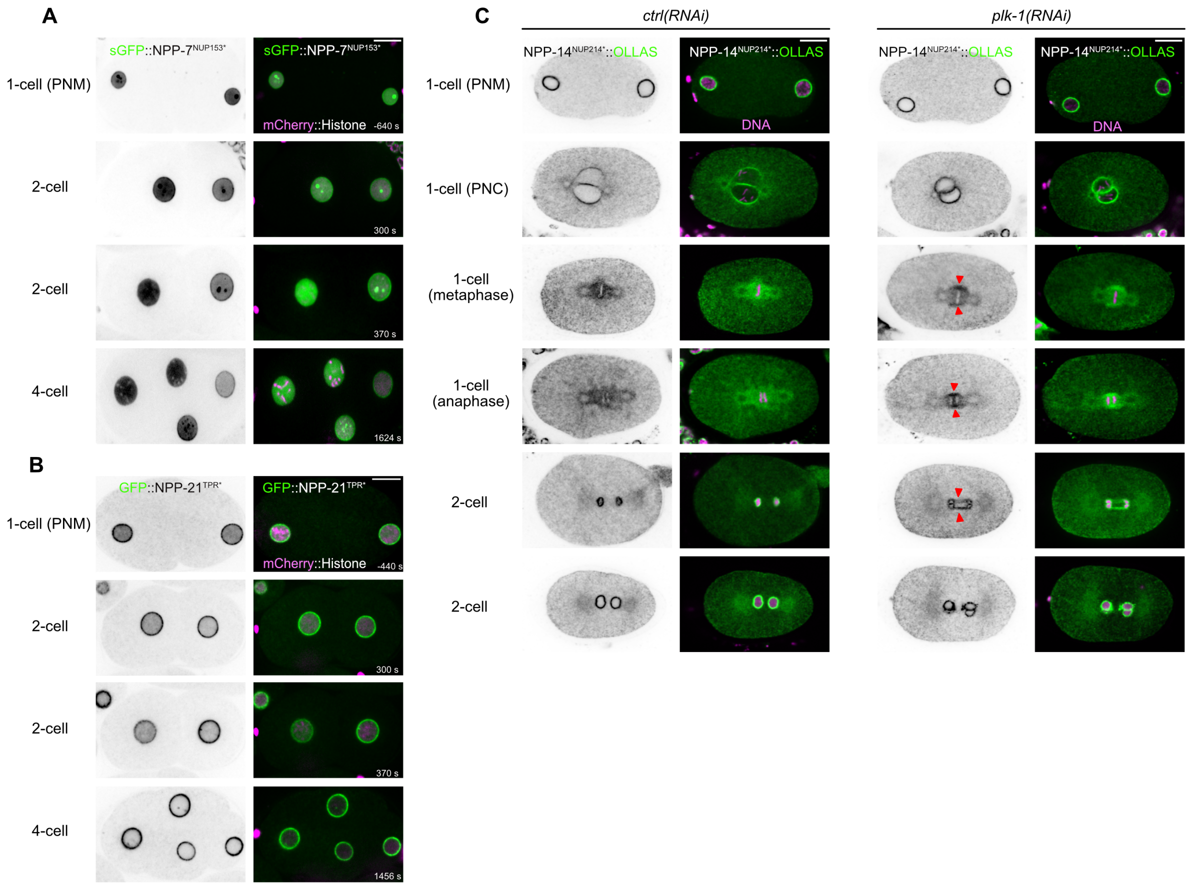

Fig. S1: Localization of endogenously tagged nucleoporins of the nuclear basket and the cytoplasmic filaments:

A-B Spinning disk confocal micrographs of embryos expressing endogenously tagged nuclear basket nucleoporins (A: sGFP::NPP-7^{NUP153} or B: NPP-21^{TPR}::GFP), shown alone and in green in the merged images, and mCherry::HIS-11 (magenta, in the merged image) in 1-cell embryo during pronuclear migration (PNM), and at the 2 and 4-cell stages. Time in second is relative to anaphase onset (time 0).

C- Fixed embryos expressing NPP-14^{NUP214} endogenously tagged with the OLLAS epitope exposed to mock (Ctrl: control) or *plk-1(RNAi)* stained with OLLAS antibody (green) and counterstained with DAPI (Magenta). The red arrowheads show persisting NPP-14^{NUP214}::OLLAS at the nuclear envelope during mitosis. Scale Bar, 10µm. PNM: pronuclear migration, PNC: pronuclear centration.

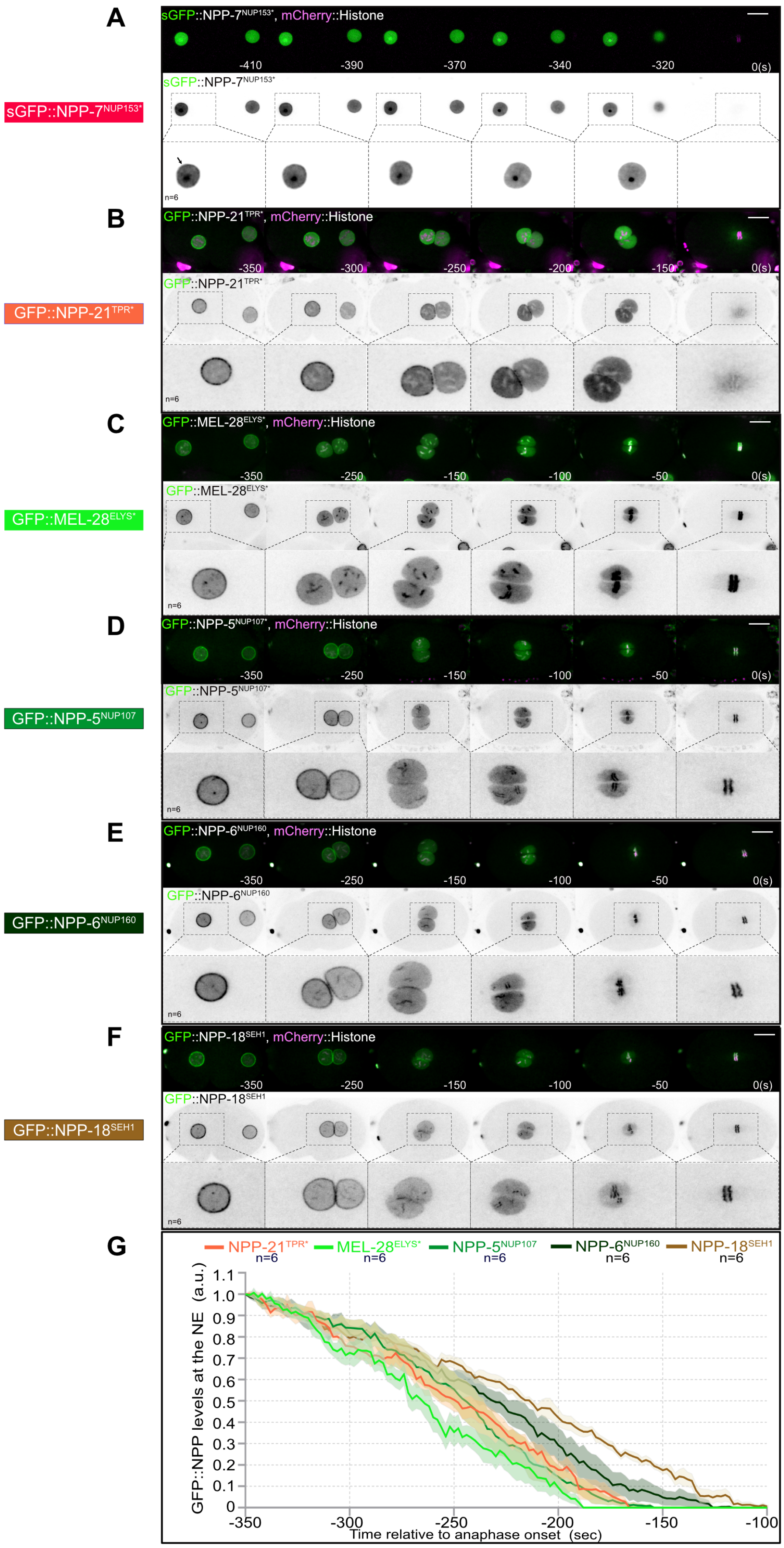

Fig. S2: Y-complex and nuclear basket nucleoporins dynamics during pronuclear migration

(A-F) Spinning disk confocal micrographs of embryos expressing the indicated tagged nucleoporins and mcherry::histone in one-cell embryos during pronuclear migration. The boxed regions, encompassing representative female pronuclei, are shown at higher magnification beneath. Time in second is relative to anaphase onset (time 0). Scale Bar, 10µm.
F- Quantification of GFP::NPP signal intensity above background at the NE in embryos of the indicated genotype during pronuclear migration. The mean +/- SEM is presented for n=6 embryos. Data were collected from three independent experiments. Time in second is relative to anaphase onset (time 0).

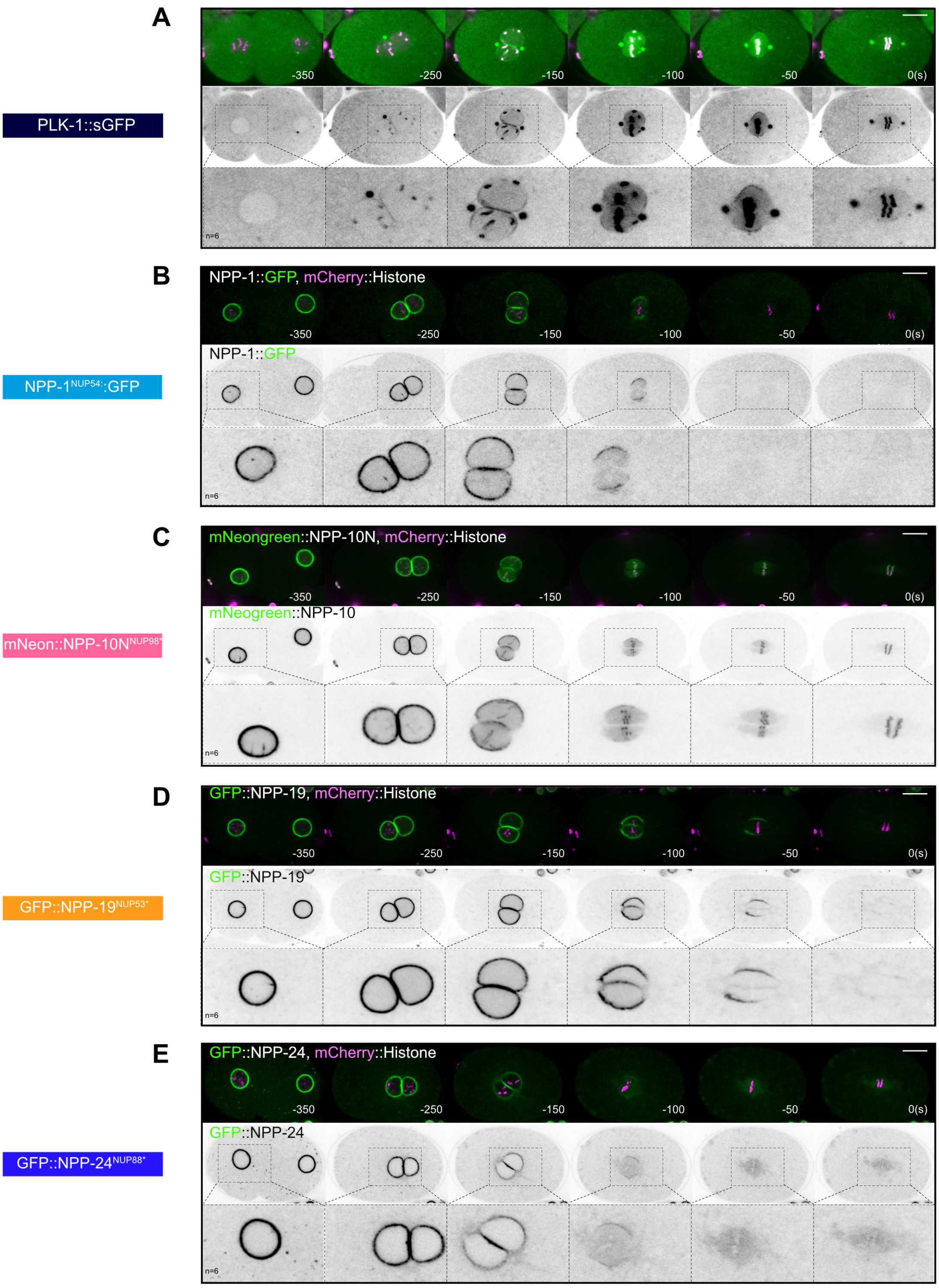

Fig. S3: PLK-1 and nucleoporins dynamics during the first embryonic division

(A-E) Spinning disk confocal micrographs of embryos expressing PLK-1::sGFP or the indicated tagged nucleoporins and mcherry::histone in one-cell embryos during mitosis. The boxed regions, encompassing representative female pronuclei, are shown at higher magnification beneath. Data were collected from three independent experiments. Time in second is relative to anaphase onset (time 0). Scale Bar, 10µm.

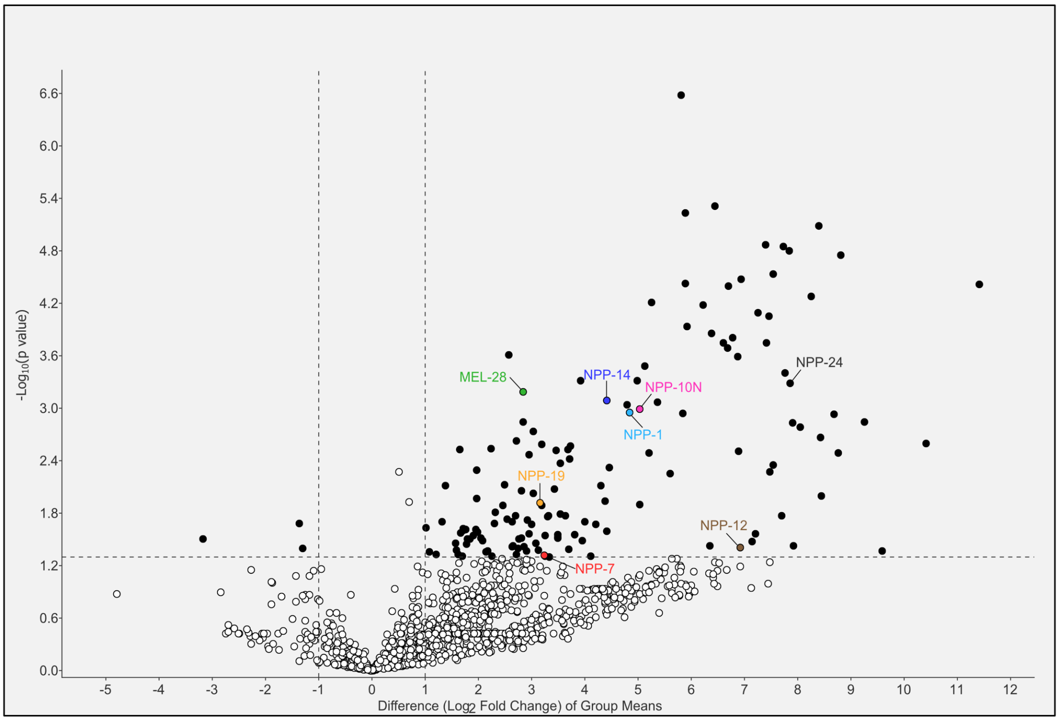

Fig. S4: Result of LC-MS/MS analysis of Plk1 PBD pull-downs from embryonic extracts. Volcano plot of -log10 p-values against log2 fold change (PBD^{WT}/PBD^{Mut}). Significantly enriched proteins (Log2 enrichment >1, p-value <0.05) are indicated, with nucleoporins highlighted.

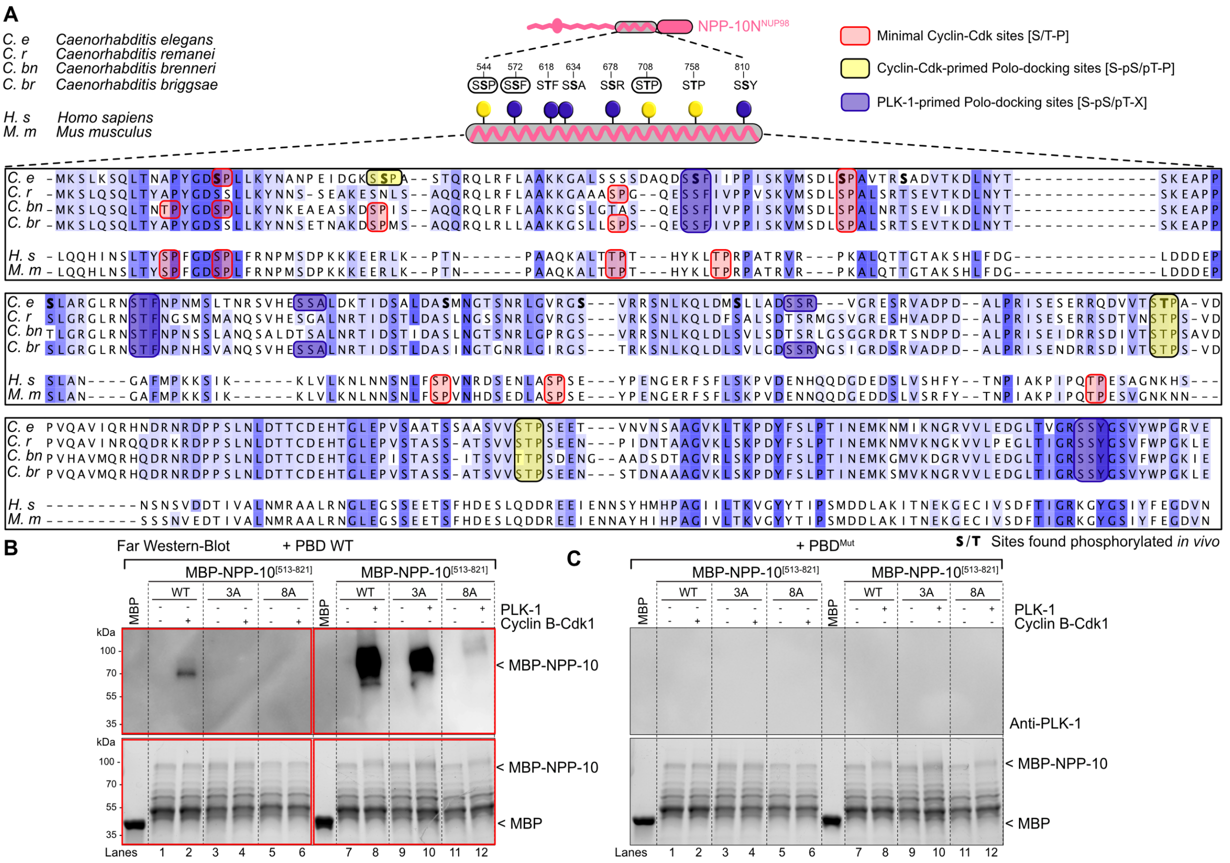

Fig. S5: NPP-10N^{NUP98} [513-821] fragment binds the Plk1 PBD via multiple Polo-docking sites

A- Mutliple sequence alignments of the C-terminal disordered NPP-10N^{NUP98} [513-821] fragment from *C. elegans*, *C. remanei*, *C. breneri*, *C. brigsae*, *H. sapiens* and *M. musculus*.

Sequences were aligned using T-coffee and visualized in Jalview. Sequence features including the minimal Cyclin-Cdk consensus sites as well as the Polo-docking sites matching self or non-self priming are indicated.

B-C Western blots corresponding to Figs. 5E, 5F and including the Far-Western blot using the PBD mutated on the phosphate pincer as control (PBD^{mut})**.** *In vitro* kinase assays were performed with CyclinB-Cdk1 or PLK-1 kinases and the NPP-10N^{NUP98} [513-821] fragments WT, 3A or 8A tagged with the maltose-binding protein (MBP) as substrates. The samples were subjected to SDS-PAGE, followed by a Far-Western ligand-binding assay using the Polo-box domain WT (PBD WT) (panel B) or the PBD harboring mutations in the phospho-pincers (PBD WT) fused to GST (panel C). The bottom panels show the Stain-Free Blot (Chemidoc, Bio-Rad) of the same membranes.

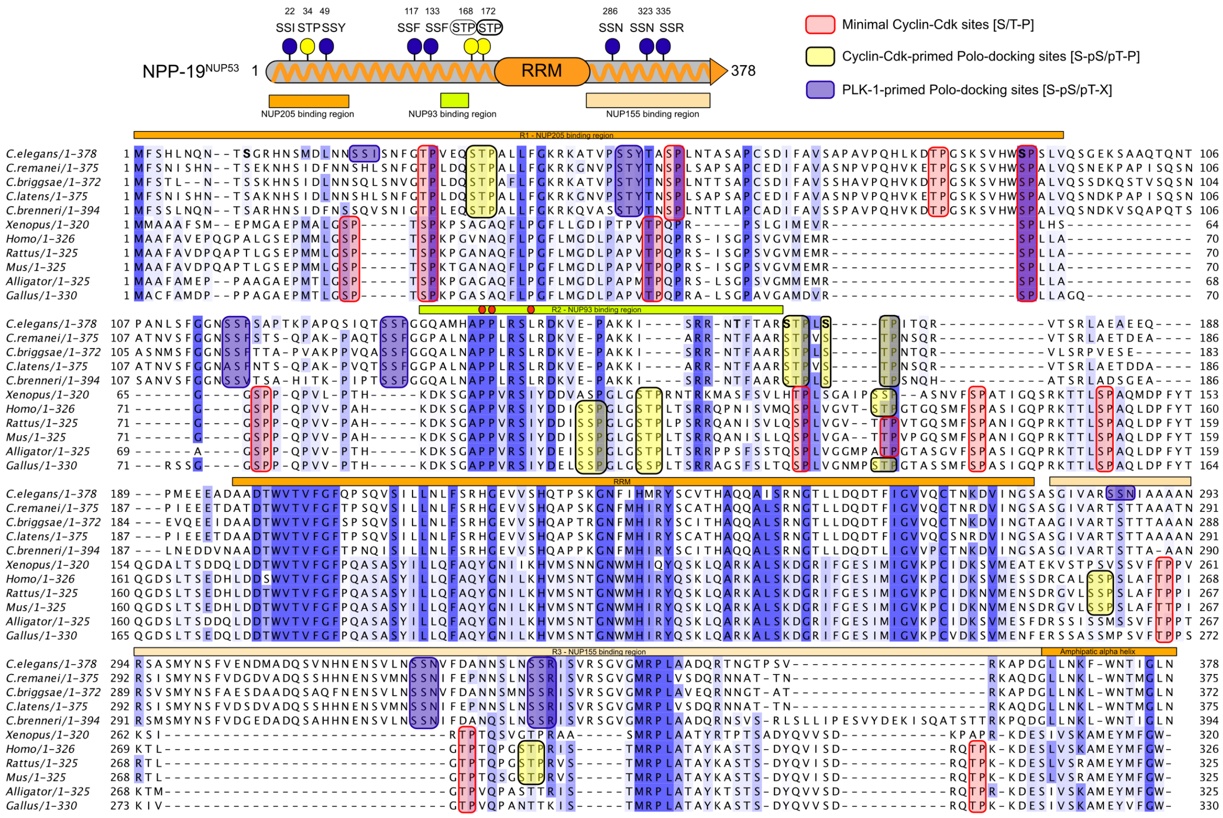

Fig. S6: Multiple protein sequence alignment of NPP-19^{NUP53}

Multiple protein alignments of NPP-19^{NUP53} from different species. Sequences were aligned using T-coffee and visualized in Jalview. Sequence features including the minimal Cyclin-Cdk consensus sites as well as the Polo-docking sites matching self or non-self priming are indicated.

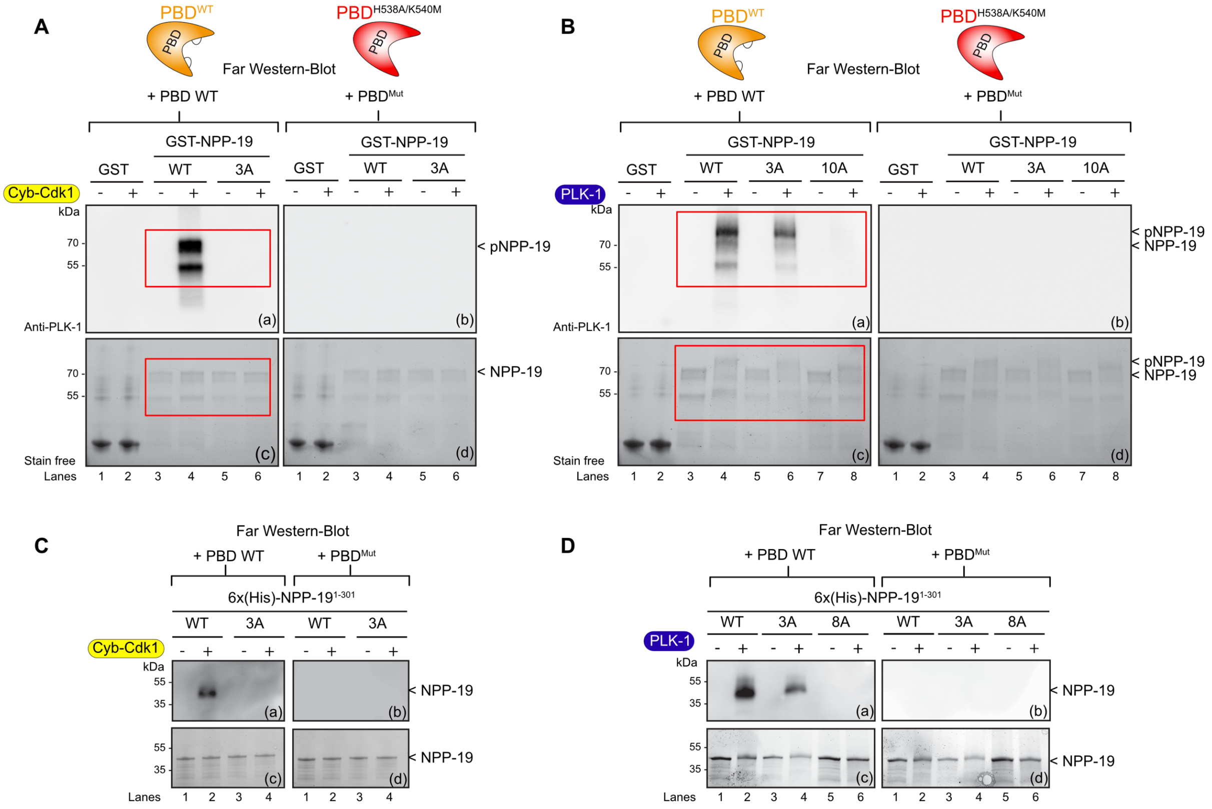

Fig. S7: NPP19^{NUP53} interacts with the Plk1 PBD in a phospho-dependent manner via self and non-self priming and binding mechanism:

A- Full scans of Western blots corresponding to Figure 6B and including the Far-Western blot using the PBD mutated on the phosphate pincer as control (PBD^{mut}). *In vitro* kinase assay was performed with Cyclin-Cdk1 and the GST-NPP-19^{NUP53} full-length wild-type or mutant (3A) as substrate. The samples were subjected to SDS-PAGE, followed by a Far-Western ligand-binding assay using GST-PBD wild-type **(a)** or the corresponding phosphate pincer (GST-PBD H538A/K540M) mutant **(b)**. The bottom panel shows the Stain-Free Blot (Chemidoc, Bio-Rad) of the same membrane **(c, d)**.

B- Full scans of Western blots corresponding to Figure 5B and including the Far-Western blot using the PBD mutated on the phosphate pincer as control (PBD^{mut}). *In vitro* kinase assay was performed with PLK-1 and the GST-NPP-19^{NUP53} full-length wild-type or mutant (3A and 10A) as substrates. The samples were subjected to SDS-PAGE, followed by a Far-Western ligand-binding assay using GST-PBD wild-type **(a)** or the corresponding phosphate pincer (GST-PBD H538A/K540M) mutant **(b)**. The bottom panel shows the Stain-Free Blot (Chemidoc, Bio-Rad) of the same membrane **(c, d).**

C- *In vitro* kinase assay was performed with CyclinB-Cdk1 and the 6xHis-NPP-19^{NUP53} 1-301 fragment wild-type or mutant (3A) as substrates. The samples were subjected to SDS-PAGE, followed by a Far-Western ligand-binding assay using GST-PBD wild-type **(a)** or the corresponding phosphate pincer (GST-PBD H538A/K540M) mutant **(b)**. The bottom panel shows the Stain-Free Blot (Chemidoc, Bio-Rad) of the same membrane **(c, d).**

D- *In vitro* kinase assay was performed with PLK-1 and the 6xHis-NPP-19^{NUP53} 1-301 fragment wild-type or mutant (3A, 8A) as substrates. The samples were subjected to SDS-PAGE, followed by a Far-Western ligand-binding assay using GST-PBD wild-type **(a)** or the corresponding phosphate pincer (GST-PBD H538A/K540M) mutant **(b)**. The bottom panel shows the Stain-Free Blot (Chemidoc, Bio-Rad) of the same membrane **(c, d).**

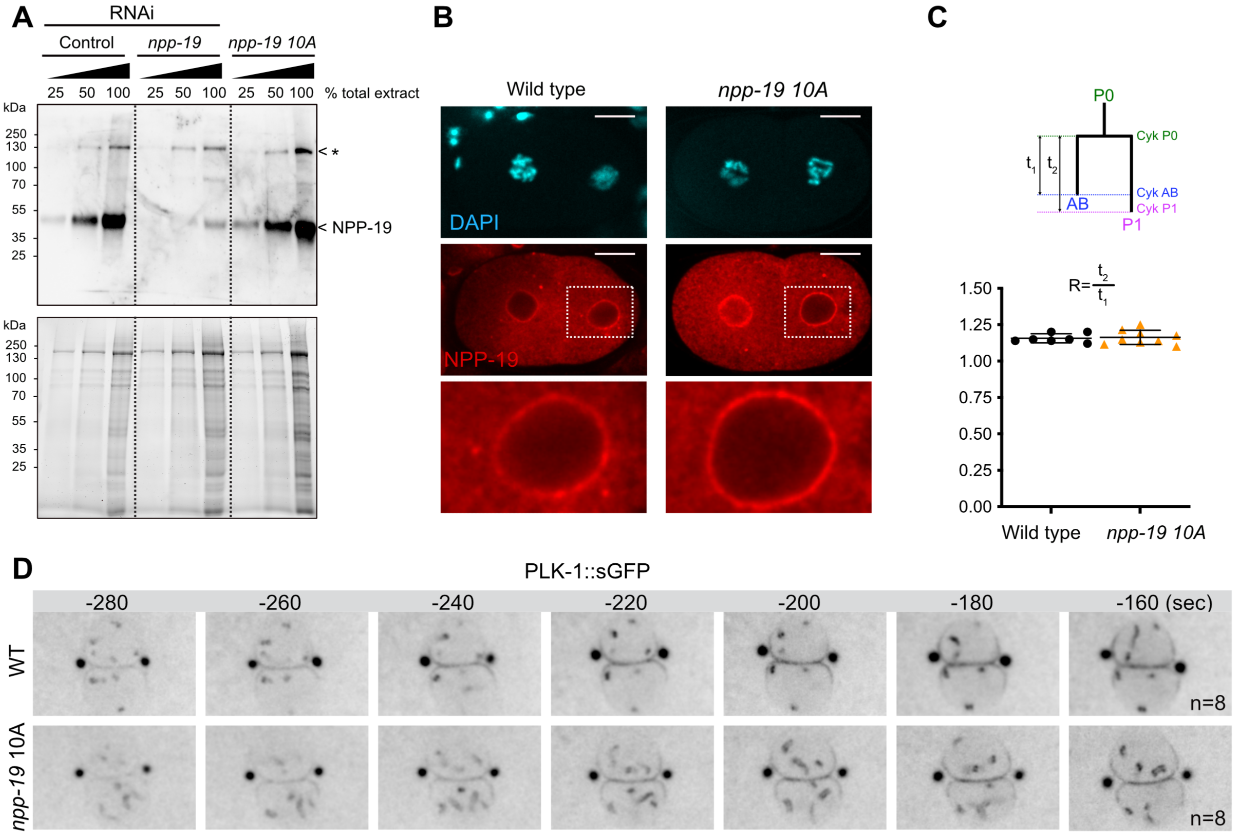

Fig. S8: NPP-19^{NUP53} 10A is normally expressed in early embryos and localizes to the nuclear envelope

A- Embryonic extracts from control (lanes 1-3), *npp-19(RNAi) (lanes 4-6) and npp-1910A embryos* were separated by 10% SDS-PAGE and immunoblotted with NPP-19^{NUP53} antibodies (top). Before transfer to nitrocellulose membrane, the gel was stained using tryptophan labeling (stain free, Bio-Rad) (bottom).

B- Confocal images of fixed wild-type and NPP-19^{NUP53} 10A embryos stained with NPP-19^{NUP53} antibodies (red) and counterstained with DAPI (bleu). Anterior is to the left in this and other figures. Scale bar: 5 µm. The boxed regions are shown at higher magnification beneath.

C- Cell cycle analysis of wild-type and *npp-19^{NUP53}* 10A mutant embryos. The mean elapsed time in seconds between ingression of the cytokinesis furrow in P0 (Cyk P0) and AB (Cyk AB) [t1] or between P0 and P1 (Cyk P1) [t2] was determined and the [t2]/[t1] ratio was plotted for wild-type or *npp-19^{NUP53}* 10A mutant embryos. Data were collected from three independent experiments.

D- Spinning confocal micrographs of wild-type and *npp-19^{NUP53}* 10A mutant embryos expressing sGFP::PLK-1 at the one-cell stage. The time indicated in second (sec) is relative to anaphase onset. n corresponds to the number of embryos analyzed.

Data S1: Phosphopeptides identified on nucleoporins in embryonic extracts.

Unambiguously assigned phosphosites are highlighted in yellow and in **bold**.

Phosphorylated peptides with unassigned phosphosites are highlighted in yellow.

Phosphosites previously identified by Gnad et al. (*56*) are marked by an asterisk highlighted in light blue [*].

Polo-docking sites (S-pS/pT-X or S-pS/pT-P) are underlined.

Polo consensus sites (D/E/N/Q-X-pS/pT) are highlighted in dark blue.

> **NPP-1**$^{\mathrm{NUP54}}$, isoform c

MSLFGS**ST***PQKQAFTFPTPNAPTTSSGTLFGSTTPSKPLFGSTAQASSTPSLFGTTNTSTPSGGLFGKTGTSTTTTST
AGTLFGAAPTTSTATPSLFGASTTGLFGTSSTTSGGLGGIGSTQQTAKSPVATRLAAFKSGTLGAGSLSGNTPTSFATPS
ALPGTNAPQSSFLSPANNLNVAPAAYRPPYSTFGGSTPFGAASTGTAAGSTLFGSSTAKPATGGFFGSSSGSTLGGLGAT
QQQQQPVVQQQQVIQQYHPFVKAVGDPKLFGNDNDGVVAKLNQVAAGLGVGKAPYKDGNQLLSFSMEGNLFERFVGIGYN
RISERTDDEGFVTLVLRHPITNLNTEERRDKILEIIKAILGGGPNVEVRYAPGTSMRTLSDGCTEICIIAKEGGFVAGAI
KLAQILNDAPKMTQLESQLQVDKTRVLPKVGMSKAQRDRYLETVPDGIDERIWRQAIKENPAPNKLLPVPVRGWEALRDR
QKAQVGESKLFHEAINALGNRVEEANHEHADAVVKMEIIRNRHKTLSYRIVRMLAQWIVSRYSRQIDTDEDVIEAKADT
LLAQMNRHNQVKFYVDKFYEILESKPDKLQESMWKMFDMTIEDEHYARRVLTKFVNICSGLYESTHQQIESLEACRRALE
G

> **NPP-4**$^{\mathrm{NUP58}}$

MSLFGTSTTAPAASTTPLFGSTAAAAPKPGGLFGAPAAAMSSGLFGASQTAPAPASAGLFGSTTAPTPASTSSVPLFGST
TTTASPSGGLFGAKTATTAAPAPTGGLFGASTAAPATGSLFGSTAPATGGLFGAKSTTSAAPTLGGGLFGSSTAPAAAAT
TSFGAPPATAAAPLGLGGIQQQNSSGGGLPNASGTATSSLTGTGDSKDASKDGEWTQAATLIRDLLPLIEDKFKKNREFM
EETENMSIDNVAIEEQIDKTRGWIEEVRRDVLTATQS***S**ERIAYLVSTDKTLCDTTKRVQEHSGSANQTTYMNAIKEHL
AELCNFYGNDMDALQERVNFLRNRFEKLLTGEKSITMEELDAYFLRCDANVSNAHHHVTELGKEIEEIRDFLIEQGYTQL
RKWSTTAASAAAPIASNSEMIVREGAEFFPSQSSLAIIGSSLRAPAAPAPAVGGLGLGTGTSLFGNTGTTS
LFGSTATKPAFSGGSLFGATTSTATSSAAATTTTPTTSLFGSTKPATTTTPFASTVANNSSGLLFSSKK

>**NPP-5**$^{\mathrm{NUP107}}$

MTDLFASGDNSSN**S**FDDS*NDEIARKNETAFKYTTIFHTNLSEKLYHELCALAFGEFDIPQGQISPLMWTRLHEIYSEV
EMQTRKLPAGSNYSASSQKYANEAVQIASVLSIYTALAHEYDETVEVSLLSKLVVEDVEFRRIYALLLWSEKAISEQQYE
KGLGDKVRKLEGIKSSRINSMMALKRPTFGAANAPKDASLDPDAPGTKEDQALEQMAMNVFFQLIRSGETSKAANLAIDL
GMGAIGAQLQLHSMLRNPLDIPLEASKQNFGEYKRSRRAKYYQMTQKLIEQSQGSEDDAYWMLISAIRGNIQPMLKAGKS
VIEKVWAYANSAVLARILAAEGAMTQETISTLFNVPLTSKSILDELRSEADRTKEVYILLRVIDDMLNDDIEDLYKFANE
TVGEFVPNDKNCQVNMLALDIFFHLVAVSYASGFEPNDDGNAVIILGFDDLRARSGTSSHKKMAAFYSRFLPEDMKLPEI
VETMKAVDSDEEREILAESLKQSDIDFGRCACTLIEQIRKDDKTKVVTLEEQIDHWHWLLIGGEETALAALEECNRLVRK
VMLSTPIDESVIRQIIRKALHFEVPKLLSQAVENEATVLSLITDGTLFEQKPGSQLAINKIEHAALEFYGLCSFVDVNNF
MITIALKLGLMFKYTPITDDELSMIGGVKRLDNTTAADWEASLRVRARAEQTLREEVLKKRAAEHNTRVGMVQQHLDTVL
PMLRGLVNNIGVRPEYFLSPRANGDPMRVHRKEIQEIRNLFLPQFFILLAQAAVRLDDTTNFNDFFTSFNNDLGLDQEWM
VFIKAFYAELNLKVE

>**NPP-6**$^{\mathrm{NUP160}}$ isoform a

MELVYGSEISFTDGFAIKPARTFVVNTNAPLHSNGFDVQPSAGVATFPHCSNYGDRIFLWRAIGQKLFIEERSLLYSITD
GSLCIDFTRTPIIPGTSITIFEEGVLCIVVPTASAIHRFYARLHSKGHDTFSILARIKEDDDFRRFRTSHSLSTPGRPIR
ASVTHHPSRNTISYCTAEGQLVIVTLGSYTESSDKHEEFTMGEVGLLGKLLGGTDKRVSDACVMKNLRP**T**SGVTRERKI
SVQSDIVFAVTRDGWVQAWNVETKKQLPSTIDLNNYFASDGKSLNRDLESADFEEEKSPMEPTETVYSIRAYSFDVDILL
IVGCDLAVSGKSVGMRVHILKVSNDQIQHLQMFETSMSVDERLVDLELIQNYFPSKESESLDETQDGELYNPLTAALFSL
SALFKSSSAKKTYSMKRISFAIQWKKGRVFTDFDWHSVRQFTSPATTKEEPIGNTDEVEPAIERPYNLSADSSIETLIDV
VFDTDIYPFDIVFRAVQIVSDNFRGTLGQVRNNNWPELSKLVDTYLTSVEFNRKFQQKTDRSIRLRLNAPQESQKTALKD

FWWALLRACEELDFAARGAISLAPIQFCGDLRIMAVVHRDRMTFLGDNNTEFLEIISSENIPKQGNVEEKFRKLPNKHFI
DLIEEASKFADRRVFLMNRERARLTNAKNGVAVVEDDNVENAYDYDSDGRFINIKTALEGPIVKLAAAFVEASTFDNSKP
FEDPHPQIFGGSFTQSVVSANIRVSVESRVHFALTLQTLLNAITEKKSRAGAPSFGDVEALSCELREIIRVYRELNEQLD
IKIVQNGAKMSIGTWLTSDAEGLSMMKKEGGYGPHGYDEIRENDFNWFVGVTTEAAIRALLSSSEILVLPRRLVLQKQYK
VLLTILNSYISETRALKPVITFYRGIAYSGTDHPVKALNSFQSCLDAFSEGNNALRKAVYFLLPKRFDVAQGKDPVEELT
ASEYFLTVVRFLQEHNHAEEVCSVAVKAIENLPIENESVQLISNTLFNHLTNRREWFQSLKLILRTTLRSETRRASIREL
LSLMLACGEWEAIATMKFGEHEQVVEDFLRDAACRQSPSEKKHYFELLFAFYVARKDFRKGACAMYEYARHIESTTCMTP
ELLRRKRDCLAVVLNLQSVLGMEPDDDRAIYDDATDTNLVFPEPSDDEDELITESDSSGENGKLPSGNKDSGSGTGNNSS
*PDNSNGTSSSGTGVDLVALKARQMAYALGSDDMDTDDTEDDMKGGSIPLFKRRKLLVLSEKDIRDELVLCSARVGLLSS
DQFKGVPPTDLNELFSLLVDNQHFDEAFDIARQFNLDSHRLFFTMTREAIMIDALRDDLNIEMAATHQAGWVRLNRRHCA
AVATAEEHWSVVRGLVDAAQAEWPGDSRPLRGSTEAFLSFKLNVPVWLHSVFENNDANDYLRCLVDYESYSIALQVLSDI
VEQETLQASQSNARTWLPYGIIDELMIRSADYIRKMSLKSPEDQVVAAEVANLRKSADQKMLIYFRKVADFEQAQKMSSR
FFDK

>NPP-7[NUP153]

MSDKSGGFFSSVGRFFSVGAS*ATKSKDGDKSNDEGTSNSSSKSSPSASPALKNILSVDNEAKIASDETSVNTSQ
NHRIGSRLRYLS*PTALDRRT*S*ASNGLEIDVDAVPDIFFSTPAVSTNRKRQLTELDKQILPQSFRSESVKRSRFLNR
SLLEPTLNSSMNNERKMDMTWCGELTANHSPASSITNFSLLSRRSGATTNGSLSTRTQEIFKKLEGANTPAKEVQRMSM
LRAGIARPEKWGSFSESKVPNGSAT*GTPPPPLKKAGDAIPSRIQLISKTMGMSARRAPYWTDLTRKRTSSKNGDTGSS
DSMKSLNGNFATAELSSLFSLDLPAPKKTASTASTTTINSSNSRKQHASIMKGPDGKPVSRNTFKLS*DDIEEVDEDSQK
LPPIPTTTLQNPQPLKLAPEYAPKRGFLDDLAFSFTAPVDLVTAVGTAKTASTASSHKASESSSESNAESTEKQTSESSG
NESDSEESEDSEVDVGEENGSEVKESPQTSADTISSAGGSNQSSKDSSPKVAKDAEPVVVAPAPVVDAGSKKWECQSCFC
SWDSTLSECGACGEARPGGSGTGPKSQPKPSEKQLVSNLSSFASNTPSTVKFGFGSGASTTTTIASTTSNTIPFGSGSSV
APLFGAPKTTAPPPTTVPATIPVAPTTIASAPVAAVTSSSNGTRVDWECPDCMVSNKASDDKCPCCSHVKYASAEASSN
VFGNRAFKPLSSTGSTISFGVGGSTATTQPSAFAFGLSKTTEVAPTSTPAFGLSAKPAVSSAPVEKPSAPEVPKTAGSLF
GNIAKPADSATTSFLAPGAASSTSSLAPTAGSSLFGGSTGSIFNLNKTNTTETAKPGLFGSILDKETHVTTTPVSVALPS
STDSTQSKSAIPTIAPTMSLFGNSSTGSFGGSSMFGNSKTELPKIS*S*T*LS*FGNPTTAAAPVSTATAE
AVKTNVFGSNSASASTSLFGAGSSSTATNLFTTKPADSTTSSIFGKPISFGDDSGTTTTDGAPAKRGLFSSDSQKLQFG
GQQKVEMPKFTGFGNPASTASTTSGSLFGGASTNPPMFGGPSSSSIPAFSTSNSSSTSGFPSSTATPFGNAGTTSTSGVF
GAFGNKPSQPGLSSSSSTNSLFGQAPADSSNPFGGTSNNGFNFGASSSTTGATAGGGGVFQFGNAATSAPAPTAAPGGGA
FQFGGNMSVPQAPAPGGMENAFSYQAPSGVGARKMAMARRRNMRK

>NPP-8[NUP155], isoform b

MSVSFMELGSADSAEAAANKVAHHVEQMMETSDFFDRLTQHGTTPVSGLGEKFYVKGAPEFVSTRRIPIPGELQMQMSN
IHSEFSMGFFTQISRVWVVIDNNLYMWNYETNDDLAFFDSSDAAILKVSLVNIKPGVFEPEIQYGLVVGTISDICLYPVF
DFVENGASSISIDSKRCFKIALDGATVNDISYTSNGRVFYTADDQLFEFVYEKQNGWFGSTNHKCRGVNQTASILGTLIS
LPFFGSSKEPLDQITIDKSRNIMYLLGRAGTVSVWDLGADGAACAKFLSVPISKIAHEAHILTQFGHDETSFHSITSIKA
LEASQSAALNLVATTAKGVRLYFSVSTGPQSTMAMFNNSGTPNERNRPQTSVRPQCLRVAHVRFAPGVTPASIYGDGPNG
VSVVYADESICAMATANRNTIFAFSNFFYPSSQFFVESTTECDISGHVWEIETVSRCKVKSRPPPRHLVDRVHPHSYFRS
QLESGNQKLLVCSNEGVFEFTHVNAVDALREALFDGGVEGNATLQLWQKLGSTEMLCLAFRILTSDAPIDERIRGKAEQI
LYSLKEAPEIVENEQQRMLDQSTTTSWSPNDSSMAEWRHRMKTPLLSSTPRTDGRNGNAGGAPPQAHFSSPFSPMLDMS
MASGHHNGNMRMSPSRRHDALFYYFSRLVAPVWNDTICEVLNGKQLTITFEPESIQSLKEEIQKLARLMDDYRLVPMMEF
NGYSSNMTDRLNHEATSLERHSLIGLRKLIDATLETLSLWLLAYEYNLTAISSGMNPQLLPNFSSRKLAHLVSDGSNLNA
ELIRAMIKYFLGDEAGTKILSESLRQLCPNLYSEDDACVTFAMEQLEAARKQGPGAARRRLVQSAVEMFKQSIGKVVLAS
TCQQLAESVEDYEPIVELCLLRAAKDDPKQLALLAYKHGRSGSDAEMLGAEKKREDCYRVITDVLDKLEDEATSEVPRDT
AVNRDLMINAVLNSDDQLAHAAVFRWLLTKNKTNVILQSKSPFIEFFLVQEINAGRGQKYFDLLWRFYEKSGNYDKAARL
LSKLAENDNWKMGLTQRCAYLSHAILCAQSCKDSTVTTNIDELRDRLDVANVQMRIKDALGCSASASARNQEFVRKLDGP
ILSLQELLLQYVVPFKLHKIKLSLLHCAGMYVEKHIFETWEDIIQDEFTTAQDEGTLCEQLSNTIGELFSVYRDTKYFPR
EFVIRRILEIGSGGVIGESVQQQRHILPPSFYPLLCKKINLSNCEFLRTASDEFRAGGDAWWTHNSRGQEYITKVVLKMA
RTVVRELENMPTAHSRRSTARDCLTHILPFIRRSCDVSASLSLQNLGTELTALQNRLSEFSN

> NPP-9[NUP358], isoform a

MSDQKPNMGRIVASVVDVQQLMTMQFDSLIKSMDSLKIEHQTGVTQLRDDIRRSEDRFQKQLSDLSTNHGKELERLHQII
HTLLARDANPLGSMIPPQQLQQQQQMLILQRQMEMAHVQAAQAQAHAHAQAQAQAQAQSQVMANLLNAAKPAIPVTQPLV
ATTAQAKSTVPASGVIAPKT*SPPEVVIPPAKPTFSTPTPAVVPKPATTGFSFGGTNPATSIFGKKPET*AS*PVVVPAA
KDDEDEEHDEDYEPEGEFKPVIPLPDLVEVKTGEEGEQTMFCNRSKLYIYANETKEWKERGTGELKVLYNKDKKSWRVVM
RRDQVLKVCANFPILGSMTIQQMKSNEKAYTWFCEDFSEDQPAHVKLSARFANVDIAGEFKTLFEKAVAEAKSSSNAGKT

IDKEIKPAAEVKKEVKQEVVIPSNNKPEETGFGDQFKPKPGSWECPGCYVTCKADEIECACCGTSKDGSVKEKNIFS*KP
S*ILQPAPGT*PKVTFGFGASAPAKEPLAQTSQFGGSLSGS*PSTSSSIFGGGTPKGTSVFGGGAANTPTFSFNKPAAA
VNATTPSFNFNNPAASTASPATSTTPGNSLFGGGLSKTESTASSTTTPSFMFAKNSESAFPKPTFSFGKQQTPSTTAP
AKQEENKQSETPKSVFGSGFTSGGATFAALSANSAKSGSIFDAANVKKAQEELAAQKKAS*IFGS*KNTTLNTTSATS
HDGDETNEDGDGEYEPEVEFKPVIPLPDLVEVKTGEEDEEVMFSARCKLYKYYSDLKENKERGLGDIKLLKSNDNKYRIV
MRREQVHKLCANFRIEKSMKLSPKPNLPNVLTFMCQDFSEDASNADPAIFTAKFKDEATAGAFKTAVQDAQSKM

>NPP-10N^{NUP98}, isoform b

MFGQNKSFGSSSFGGGSSGSGLFGQNNQNNQNKGLFGQPANNSGTTGLFGAAQNKPAGSIFGAASNTSSIFGSPQQPQN
NQSSLFGGGQNNANRSIFGSTSSAAPASSSLFGNNANNTGTSSIFGSNNNAPSGGGLFGASTVSGTTVKFEPPISSDTMM
RNGTTQTISTKHMCISAMSKYDGKSIEELRVEDYIANRKAPGTGTTSTGGGLFGASNTTNQAGSSGLFGSSNAQQKTSLF
GGASTSSPFGGNTSTANTGSSLFGNNNANTSAASGSLFGAKPAGSSLFGSTATTGASTFGQTTGSSLFGNQQPQTNTGGS
LFGNTQNQNQSGSLFGNTGTTGTGLFGQAQQQPQQQSSGFSFGGAPAATNAFGQPAAANTGGSLFGNTSTANTGSSLFGA
KPATSTGFTFGATQPTTTNAFGSTNTGGGLFGNNAAKPGGLFGNTTNTGTGGGLFGSQPQASSGGLFGSNTQATQPLNTG
FGNLAQPQIVMQQQVAPVPVIGVTADVLQMQANMKSLKSQLTNAPYGDSPLLKYNANPEIDGKSS*PASTQRQLRFLAA
KKGALSSSSDAQDSSFIIPPISKVMSDLS*PAVTRSADVTKDLNYTSKEAPPSLARGLRNSTFNPNMSLTNRS
VHESSALDKTIDSALDASMNGTSNRLGVRGSVRRSNLKQLDMSLLADSSRVGRESRVADPDALPRIS*ESERRQDV
VTSTPAVDPVQAVIQRHNDRNRDPPSLNLDTTCDEHTGLEPVSAATSSAASVVSTPSEETVNVNSAAGVKLTKPDYFSL
PTINEMKNMIKNGRVVLEDGLTVGRSSYGSVYWPGRVELKDVALDEIVVFRHREVTVYPNEEEKAPEGQELNRPAEVTLE
RVWYTDKKTKKEVRDVVKLSEIGWREHLERQTIRMGAAFKDFRAETGSWVFRVDHFS

KYGLADDDEPMDGS*PPQQALQASSPLQVIDMNTSARDVNNQVQRKKVHKATDAHHQEIILERVPAPAALGDVVPIIRR
VNRKGLGGGTLDDS*REESCIGNMTTEFNESGHDSIIEEGQQPEKKPKLELLADLEYESSRFIRNLQELKVMPKANDPA
HRFHGGGHSAKMIGYGKSKLIDIGIVKGRSSHVGWSETGCLVWSAQPRHNQVLFGTIDRTSDVNENTLISMLDVNVHVSE
TSRKGPSSQSNSVKSSLTSNFVTYSDSYSSMFAKYIDVAQAGGYDGHVSVWKLISALFPYERREGWSFERGEEIGEWLRT
EAVKSVPDDRSADTSSNGVWNQLCLGDIDKAFQIAIDNNQPQLATMLQTSAVCPEATVHCFKAQLDNWKKCETLHLIPKE
TLKCYVLMSGLSHYEWDQDGKNHSINCLDGLNWIQALGLHVWYLRAWTGLEESYDAYQKDVNAGRAASNRGDLPGELIKL
ACESQHSVEVVLDCAAGENPNDYFLQWHVWSLLYSVGYRTMSKTSETRLHRNYSSQLEASSLSKYALFVLQHIDDDEERS
TAVRSLLDRIARFTDNDMFDSISEQFDIPSEWIADAQFSIAKSVDDSTQLFELAVAAKNYLEICRLFVDDIAPTAVVAGD
HDALKAACAMVRPFENQIPEWGATGMVYTDYCRLINLIENDAEEELLQDVLESLETRLHAPTISKNSLQKLSLQTIGRVL
FEYRADKNTLPEWTKLLGHRQMFKIFRDRSSWGIERFTIEFD

>NPP-11^{NUP62}

MFGGSAPKPSIFGGTAATTTASSGFSFGNSSTSTANTGGNTNTTGGFSFGSAQPSTGSTGLFGNSTATGSMFGGSSAAAP
APASAGIFGNSGAAAPAPASTSIFGSSANSAAPATVTFGASAPSAGAGMFGANKPAAPTGGLFGSSTSTATTAPTGGLFG
SSTAAPSSGLFGSTAAPAAPGGLFGSTSTSTAAPSGGLFGSSAAPTSTAPAPSGGLFGAAPATSNAAPTSGLFGNSAPAA
TASSGGLFGAAPKPAAPSGGLFGSTAPATTAATTTATSGLFGAPTSAPSSAPATGGLFGASTAPAAATGGLFSIGAASAS
TPSVGLFGNSSASTTAAAAPATAPAAASTGGLFGATTAAAAAPTSSTTGGLFGSTAAAPAASLPTGGLFGSSTPAKTPAA
PTAGLFGASSTTTTSAPATGSLFGTAPATTTATSAAPAASTGGLFGASSTTTPASTAPTGGLFGAATTTAPAAAAPTGGL
FGAATTTAPATVGPTGGLFGAATTTTPATAATLGQTPSTVSATPSLPTTTSTTSTLPKPAEATPTLGLGLGLTST*PLAK
GTGWTTSGLKGAATTSAGLKIGASGSDTLSEEEIKAGLGGNDTKAFFTALQEVVNSYHSEIAKQERVFHNKMLELNAYD
RELITLEPKVLGLYNEMDDLSGSCKKLHFNVASMTSVLNDIEQNVVELENKLSLPEWHTLDYKFPLDSRFASRHDVQRVQ
IAQMMLNVDSQMKCADFDLDQITKSLNTMQSTVLKTKTETPLEKTELIMKKQLQKLMDLSTQHDATRDKLNKLKDDHNLK
TNNSSKA

>NPP-12^{gp210}

MIILRSLLVLALVQITISYRLNVPRVLLPYHPTVPVSFVLEVTHPTGGCFTWRSTRPDIVSVKRIETNEAGCSDKAEIRS
VAKPGTVGSSELSAVIFAEDKGSGTTLSCGVTVDEIATISIETTTKVLFVDAAPARMTVDAFNADGDRFSTLSEIALEWE
LASTSSNKAKPLRIVPFEQSTYEAPSEIVKLEKNRKKGYLILIEGVGTGTATLTTKFSDAYLQKVAAHNVELAVVANLLL
VPSQDVYLPVHSVLPFQVLIVKQRGTEIVNMPNPSYELQIDGGDVASLDKKSSSVRALTIGNTAVHLLSSHVDVRAKAGL
RPPSTVIHVVDAESVQWHVSGDNWMLETGKQYTINVELLDEHGNVMFVADNSRFDTHIDEQFLHVDFKSENGTWFLVTPL
KPSKTTLRTKFVAIIDAKGNRIAQSGKIGGEQRVTIVDPVRIVPPVIYLPFVSEKRSQIDLTATGGSGLFEWTSEDGHVA
TVDLLTGRMTANSLGSTKVKATDKRNDQLRDIASVHILEVSGIGFGETVRETFVGDTLTLNIKATGLTSDGLLVEMSDCR
NIRAHVQITDNALLRHESSADSSLPMMGTGCGTITFKGLSSGDARVSISYLGHKASIDVAVYEKLSISEESSSIALGSTH
PLTVSGGPRPWILDPANFYKTQETKQSQLQVTFENEKVLFKCGSSEVTEAVRLRIGNLKSSTLPLPIHSEITVSICCAKP
TRLEIFDKKQRPSKCPLNVHSMLINTNVELVLRGSGVCNGAATPLASINGLSPKWTTSDSGLLTVNRHGIEADATSGKKE
GQVTIQAQAGSLSTKYEITVKKGLNVEPARLVLWNEAVSKGTFTITGGSGHFHVDNLPTSDSPVAIALRARSLTVTPKNN

GQVNLRISDACLVGQHADASVRIADIHSLAIDAPQFVEIGQEVEVEILAQDETGASFEKEHRPLADAQLDASNNHVILTK
VDGLRYTLRANSIGTVSLSASSKSSSGRVLSSRPHTVQIFSPIFLQPKRLTLIPDSKFQLEVVGGPQPTPPLDFSLNNSM
IASIEPNALITSSELGYTAITGTVRVGDGHVTLDTVVLRVASLGGIILSASSRKVETGGRVNLRLRGVIAGAEDEEPFAF
GGAIYPFKVTWSVSDPSVLFTTHPLGGDVVEPTDNQFAIWFNAIRGGSVTVKAVVELNEKARKHFTGRTSTFTAETTITV
EDGLSLVQPEMDINTVRVAPNSQLKMVTAWSQASFSVPSDFSSRIVISADGHLITNGKEGSAAITVRNVNSPDNETVLIP
VTVSRVASLDVHPTIELKSAFENSPLIHLPVGAQIQLNVVPRDARGRRLAAASNSINFRPHRFDLTDIVATNSNQTLTIT
LKTAGDTVLRIGDASNTHIATFIRLSASESILPRAAHKYANDLVVSDVICLQSNIFTVDGSRWSSQSSSEGRISWLDENL
GVAQLTKAGNTFIRLHADKQTIHSKISVVLPSSLRFPDGQKPEFVSNDEHSVFVIPVIAATNNTSGSKVSSIYGECTADQ
IRSFDAIGAPFECQVAFTRRSKIISAVNWLTVSAVFSPVFGYGCEIRRFDSSVSTSSIVVPEELLKDQFDARITAKWISD
GTVQVNDATIDVPFHFAFIVEEKELVFSNMNQIEAALSIWAPTYDSKHIVVSGCEGDIVSVEKTSRSSDKHSAKANVFYN
IRLNIKSAALFTEHAKKCQISVENTLTGQVIRVPVTVQLLDETAKQVYNALESRGVVDVLLILAHKYSHAIPTLLWTCLV
GIIILVIGIYVKMNVFDKTGSFGDNTLNNTTHQTSMASSLSSTNVSLREPVFRSTPIAGSPQVSLPTARDRLRNQMGSG
GDNRLWSY

>NPP-13[NUP93], isoform a
MLQFTEILGRVDHQGFAQLLANPIFQTNRVGQENSQQFTVRNGVEQELCGILGGSEGDWTRQTHSVQKQMMFGERGIELP
SRQPKSHTAEDTLTDEGVVDVPEAVMDDIDEEELDEDISRVKVETDRAFFNHMLLSRPAAVPMNPAQAMERDNLPFGGGK
FVNKNISDRRELIFGDKLHKFLKNQGKKSLVDLMKEAIDESGTDGALGDVWNDVTSVLNRKTSASRDDLTTTAN
LVEDACKYLQAVFTEHMQTVVERNLEVAERGGIPGTRGLVNAFLKVGTEESFQPEDDSIDGMPTWQVTYHCVRAGDM
KSASETLNRLKSFPQCATLVAALNHVAKHGKLDSELKKKLKVEWRHNLAHTKDKYKRALYAALLGGLDSAALADTLENWI
WFKLYPLHVDPQLTDVLFKEVQKAVSVDYGEQYFMSNGPSEFQYFFTALWLSGQFERAIYLLHECGQRVDSVHVAVLAHK
LGYLRMSKKSTDEMLVVDQNDSTKCHLNLARLIVAYTKSFELVDVPRSLDYWFLLKGITTPTGSDVFEMAVSRSVYLTGQ
TDEILGKLTPDGRREKGLIDEYLDDPSEVICRVASDTEITGEWDQAVGLYLLASKPTNAAILLSSEISETLRTENKEKIA
DLVHVAEQFKKVQRGCQASEYATLSLLVDLAVLFDHCRNEEAEIAYGISTHLRLIPTEPDQVTVIVNEFHMVPQKVREVL
PDMCLHLMKCLVDHCIRQSTTQANRGANSATTSMFSSSNRYVKQIKAIVLYSATVPYKFPTHVTSRLLQLQASLGI

>NPP-14[NUP214]
MSNEDVAEDVSQVTDFHFHTCRKFRLFSSKSDGYSQNEINIRNRVSQLGVTFVTVNSNQLSCFHTKSLLGYKITRENMNV
EVTDLPIKTIRLHGVVLINDMGVNSDGTVLGVLHTKNNDVSVDVFDIKKICTSSSIEPFKPLCTTRVGTEQINQGSCLEW
NPAFPDTFAASSTDRSILVAKINVQSPANQKLVGIGKFGAVTTAISWSPKGKQLTIGDSLGKIVQLKPELEVVRSQHGP
ENKPNYGRITGLCWLATTEWLVSLENGTDQDAYLMRCKKDKPTEWIQFHELSYSSSKWPLPPQLFPATQLLVDWNVVIVG
NSKTSEISTVGKRDDWQTWVPVEGESIYLPTTSSGKDTVPIGVAVDRSMTDEVLLNPDGSQRHRPSPLVLCLTNDGILTA
HHIISTFAAHIPCQMSSQNLAINDLKKLQFDSQKPISAPPSDQTPVTKPSTVFGQKPEAETLKSSLVGSPSSVQTPKPS
SSLFNPKSIASNIETSQLTESKPSTPAAPSSQPKIASTPKSEAIPKISDKTLEHKKAELIATKKQVLIERMDKINDSM
AGAKDATMKLSFAVGKVKTTIMECADVVRASLGDSKEVMDELKNLILSIERMSDRTQHTVKEMDFEIDEKMELVAGVEDG
NQVLEKLRNMSETEKLMRFNKLETAADLLNGKYEECSDLIKKLRMSLSEKESLRKQAILS*PLRLSSNLNQLRSGSETE
LALKVMRNVSKIIMDTREQIQRTELEFVRFQRDVKFQNFKKGKENLNFTQPLEMSSLDGDAPQGKSLTDAESIKVRQALV
NQIQKRGIVKTRNVIVESYKKSENSAAMKNDLLDTSNLSNAILKLSMTPRRVMPSSSLFSASPSTPSTKSDAATQADE
PPIVKTVVVTVESPAKPIASAPAVSSPLIKLNTTTATTTMTTPKVTVPKEEANKTQDQKPIISTPASSSIFSSGSLFGT
KTQTPLVSKEESTLTTGVPSLINSSLSISPQEIEKASSKVETLNKTEEVKDEKSENEVTPDLKSEEPKSLETKVKEEP
KPAVQTPVKEEETGSNIQKTPS*FSFNSTTTPKSTSSTSSIFGGGLKTQTPSSSNSTNIFGARTTTTATPTPASNT
SSIFGGGSKAASSPFGSFGQAGCQPAKTSNPATSTASVTFSFNTGATSASAKPAGFGSFGAGASAKPSSVFGGSVTAPT
VPNVDDGMEDDSMANGGGSGGFMSGLGNARTSNTSGGNNPFAPKTSTGTSASSSSWLFGGGGNQQQQQQKPSFSFNTA
GSSAQQASAPATGTSSVFGGAPKFGSQPAFGAKPFGGGANAGLSKNASIFGGATSSTTNNPATGGFAQFASGQKTSSLFG
GGATPQTNTSIFGGGANTTPAPTSSVFGGGASANANKPTSFTSWR

>NPP-15[NUP133]
MSGRDLELTLDRVSSIEYPALVKEAFLNNWHASAHRSEVTSNCASLNDRYCWVLSRNQIFIWERAKSSHRAIIPTQLPLP
TSGLPRSVKCVVVYDGVHRGANKTPCPGILVVSPEGVLRHWTSIESQTYIEEVLDINNEVALRVELTDEPIDGKSASFLL
TTTSGTVYFLNGKGQDSAKTGALECNKVAGREAHGFRRRLS*SIMFGGESKESTSLITNSFQHQSKDLLVVTVSPDVLT
VYNMYTPCELWSLKTKEFFQPKIASFFEADLKRTPLKVRARLIDAAVFRDGLMILIGGTHEESQSVHMFMVWMSANWQTE
QPTGVVWSARVPMNEHRALFSKIDDSIYSNLTLCIPKNTAESKKADRTDGIIIINPYFAVSLYLPFDLAKPKKPESLYRH
VSIPPRDQLLGYAICSQYVYIMMLESGVSTIRLLPRGFADSSIYTHEQVVVPSLSVGTDDWPILSELLSEMVASGLPKTP
LYQSLHRAFELFAEKHMAESEEELKAIIKMPDQEIARIVSQFLYAIIDYSDAANKTDTELHAKRVLTSRIMLFLKHMGVY
ERIISSPLGISRGGILSLRVGGTMLGEVSERVAASTAIWTWKTSNETNSAVFDAIIEKVLRIPEVQDLGLKDKDALFGRC
GLVHHIPVVAAQQLEKNVIGKTKSHRFEVFHAVCELLSGIKETIISWRNCRTKVAIPKFPIWWTLETFASCYRDVAEKII

EELKNGSSTDSERARLLMYILSIYDFYLSESDSQPDNDKVLQEMIALGKPADAMELAEKHKDFGTLVKNYLTTDVGTRQK
TFERYKKMFEKDDFEMYLCDYLKEHGRNDVLLQQGGSRVDAYLDNFKELRYSREIANKQFGKAALTLMSLADAETKSFSK
FVEFLTRAYYCACSSIDGTDVSEVLDFYKRRYPEMKHRKRIPTEILKICFGNDLDAMMSVEDMLEWNMAVQPNDEASVEG
FARAFHLLADLLAVHPDSDELKKKIDKTWKALVDYDEWNRVRSKEDVEKKTIFGKFCNYLINSYPADKGDSFPIWMPISR
RLIFPTDIDTVLDECIANTTGNHLSWIKGHLKWIGEQLCKQALLPKSAFFRPDMKQVGSISQAALEAFGPILQRREQRFI
DQLNRDSMMET

>NPP-16^{NUP50}

MNSLIPPPTSEQQMNMFRLRDKMSLLNAEFLKVINGYFTEKNHYDFSGTMKSYMDHVAQLKQIYKVDDDVAADMTVPRR
TENSSESSGETVAPRKIAKAVRKNGTPKNPLNS*TVFAAS*S*PAATVASVPKFGDISVITKETP
APLAKTAEPLVAPAAPATARKRAIRGGGPLGGAESVVFKSGEDGQAATSSVKIPATTIKFPEPTKDFWTKKSDAPAAPS
NSGSLFAFLGKDGDKPKETPKFSGFSFGKKPAEPSEEPKAADSTPKLTFGS*PKEADLPKPASSLFGASPSKPLVFGGS
SADSTTSAPKPFSALSTAASLFGSSSASTTTTATQPLSFGSSSTGGSSLFSGFAGLAQKAMENQNQAKPEGSGEDDEGEY
VPPKVETVENQEPDAVLSSKVSVFKFTGKEYTKLGVGMLHIKDNDGKFSVLIRAATATGTVWLNSLCNKAMKATVVDAKG
DRIRLTCPSSSTEMATMMIRFGTADGAKKFTDKILEVAV

>NPP-19^{NUP53} isoform b

MFSHLNQNTSGRHNS*MDLNNSSISNFGTPVEQSTPALLFGKRKATVPSSYTASPLNTASAPCSDIFAVSAPAVPQHLK
DTPGSKSVHWSPSLVQSGEKSAAQTQNTPANLSFGGNSSFSAPTKPAPQSIQTSSFGGQAMHAPPLRSLRDKVEPAKKI
SRRNTFTARSTPLSTPITQRVTSRLAEAEEQPMEEEADAADTWVTVFGFQPSQVSILLNLFSRHGEVVSHQTPSK
GNFIHMRYSCVTHAQQAISRNGTLLDQDTFIGVVQCTNKDVINGSASGIVARSSNIAAAANRSASMYNSFVENDMADQSV
NHNENSVLNSSNVFDANNSLNSSRISVRSGVGMRPLAADQRTNILQGTPSVRKAPDGLLNKFWNTIGLN

>NPP-21^{TPR} isoform b

MDVDAPLQAPEQPVADADDEEANWEMEKAEMKRIEFNQNRELTDMRERVEDVSRSNSRMLEELTRHAAEIKEHINRQRVL
ESTRNELTDKNLELETVVSRLKIEKEERDAAVVNATKLAQTAQVETFALKDEIKKLTNEQASLRHSKEALEKEIQGIQFE
RQKYATERSLHAESKTWLMQEVSERDNKVSSLRLELSNKDIQGANERLQYVQQINLLNSQVENLNEKLDMLKFTNADLIK
RMENTELSKVSEIANLEEEIRCQTELQRVMKSSMEESKNAADLFKDQLEAQENVLVEVRKVLQEHQDEMERENLAHADAI
KHRDEELAQTRAELVKVTEMMKSMSDVKLNVS*EEELSELAPAAAETVRYLRGGQSLSSLVLEHARVRGKLTEVEEDNV
NLRNTLEELLETIDQNKPQMISQKMVTDELFDKNNRFEKQLDLAESERRQLLSQRDTAQRDLAYVRAELEKYQRDYEFVS
KRNAELLYAVERQSRMQDPNWSEQADEQLFQNIVQLQRRNVELESDIENAKASAAQAAINAQSEEMAQLRADLAVTKKSE
AELKTKVEQTKAAFDSLKERTEHFKELVRDSVTAAEARTARLRAEEAIAAKVVADATIERLRTQAEDYKADHLRREQDLE
QRIRNTEANIASVTETNIKLNAMLDAQKTNTASMDQEFKSALKEKENIFEELKKVTAVNAENEQRLVDLGRQTLEAVEQA
GSLRVRVRSLEDELQSARTEINSLQFTANGQRNILEKEEQVRMSVVEMANFLSRVEAERLTHANTQLDVLRLERDSLKAS
TTRLSDQLTHTKNESKLVQQRLEKELEIARQRLSEKETQVTRDEMELADLRSKLASMHSQYTGSDASGMTPDRLKREYM
QLKTRTQFLESELDDAKRKLLESETTQKRMDAEHAISASHNTVLEENLKQSEQMGVMEKERLVAKAKCFEDRSKQLAESL
EQNQKKLDELRSKNDEQLFAHERETNELRRQLQVASLNLDGVRRELEVVNNNLISMQNEATRNSSALEQHTTIVRQFEDR
ITEIESANLRLQTELNNKCAALVAESTAKREADQMIEHAERLLQKKTEELNSIEEENRQKQAEYDEKLAQLSLQYESLSA
NLTNQNTTMEVKVNTDGSSSTVENLQSLLQFVRQSKDEATSRAMTAEVEMRRLRAETAEYERGRNELLRKIRDLETEKIA
TTAALVEKASLMEKIQALTDVHNINAKLTEEKTKLQAQLHQIQKEKADLENQRSRLSASNEEQKLKIASSDQEANQRKRE
IEQLKQRVQTNARGAASQPQLDQLKAQLATARQESAAATAKAKAAEDKFNQTRQLAIKYRNENTELKKLAEAPPGEGDPC
AARLKLQFDDFTAKINDYKTEIENLNMKVLRMGILEKSLKNTNDQINQLKQENLKLTENIRMAQLQSVSATDVE
SKPGPSSASKSVSSIRQT*PTKVLDPLSSAAKQPNEPDQTTGLKTSQQPPSFAAKRPS*FAPTPTSQQKVSPVKR
PIPPSIPNEPLDIIPPVPSDNIPDPTPPTNSFGTVLPVPHTFQTSVRVPTQSLFSSSSTTTVQPQPEKKNVLPSIDSAPS
TPGGNSSMVTTTSSMAPGQSIFGNIGNVPVPTTAPTDNLALPEESVIEGSAGQSSLVSGSIDQRKVQDIDLVANDGES*
RDS*T*NVGGVSSSDVRRKRT*ANDFELSEAKRLRES*PNET*VTS*SETRQQSNVADIPELDDDDGVLGMEHEVSDE
DPNDNTIQEQRPDVIDLENDEEVLEDEMDEEEDDDSFGNDEEFEDEEEIPEDDDDDDVVVLSDGDDEPANDNDEESLNDI
DDDDGIEEIEMVEESNNRDIEEVLGGEDS*QPS*LDDQDREAASAVEEAEDEGRDPLGTIDEPSAPADPTGAAGIGSSG
RMGQDVQRVRLPTGLRDAEREDQCSSRFFSNETNDERPAERLTARNLARMQRPTRGAKPTRGVYTPARGNRGGRGGGTA

>NPP-22^{NDC1}

MMGDSHSSFTTTTDEHLYNQFSPGRRKNDFPAASSSSSSPNLRRSPNRTVSSPRVQQKPITIFDQIVDWFQAEISVR
KRLAGAACGYLSTIFFIVTVSILKLTIWAPFSSVQDSLAWWIYPNAWASIIFVGIASVAMSLFSIIKFCKVDQLPRLAAT
DTFALAGVALEFVTRLTFVYTAFCVADFSFSREFAFVAISLAIAISSALVVFRSDYQLNFSHIQVNSVKTLIDFGTSLPY
ANISEICGIDAAISYTAAVALILVVGPMVSGFSAWWLLLNIPFHVVLFGLCFTQQFYSKISMKIVNQIVMKPISFPFPPP

YTVHSPTPEQTRTLPNVIETDDSLLKFFALHDLRTIAWNDEKRRVDVFSLSQPGKHPRNWKAVSLPCVRMLDELCSRMTV
SAARLVGYSWDDHDIENEDVPRDALLMPRKMREMAYRGTGQSRQQKSMAPIRSHNTQTVGLLSKISNFLGFGVTEKLVIS
RFDAHMNAYAAEALYMLVVDSMGEDRFGVVQKDLKDLITLLCKLIAAIDTYERAKASVADKSDVTFLRIVDASLKSSLQR
VVTTFGSHLSSLNLPEEHSRTIRMICLTDEL

>NPP-23$^{\mathrm{NUP43}}$, isoform a

MAVVTIT**T**DTED**S**EIPQRTEVGSSTPLVDEIMQHADVKLSKILFTGETSSQIISLGKGRGRCISLWERDDG
IDPFKVLATKNSEIDPNDACTMTDNRVCIGYADGSLAVFSTDKDDLALMSRIPSIHSGSASRKICRHGNSILSASSNGSL
VAVDVETGMPRTIFTGQAGIRSVCTTFGTNVVMAGDANGQITMWDLRENNEHSNTLDPIKTLIPSKKALDAVTALCSHPA
QSNLVCCGTDDGIVGLIDARNVRGANITSTYLVAKKAISQVMFHPKCGDNLLVSSNDGSLIRIDASGAPIAVGPRSTKDT
IWLEGDLVNSLRLDPIRNESVHSISSFDIRSDTVVTASSIGLISLYQQLPFFPNSNFGRF

>NPP-24$^{\mathrm{NUP88}}$

MLITHLIDSLSDRAVITPLTPTSVIIYDKNELKVYVGFTNNRNSLEYTKEVVLLLSTEIPKKAEIREIIVSKNGDYVILE
GPRSLFVVRIGAEILVAKPDRLPSECFCECYPLHDSLLLQNISLSVVKVRLLPEKCDEKTFVTAVLFSDNCIRFYNLQKK
FDSLLLAVDFRNHLHQVHDENVANN**T**FGLQKALVSFDLIPPKPNTSHFSIISIDSDCDFYTSFVHFSCFKEGYAPRIHR
IEPVDGLPCDPLDLRYIQTTNPRICSVFVLVSGGGVLSHLVVFPNEFGEFRFLVKDQLRLPSSNGDPRIVQNQIRSLKVS
RYEIATSSSLFSVNIFPWFEALTSISPTSTLEKETRVSELVDAVIPSDELSNTTKWTGARALRAVSVQLTQSLATEEEEL
LPESENIMHLVILENKDGQPAHLFNISTFDNIWSTENKTSFGRDSVSQPPMKSTGSLEQQLAALKPLAACVISEKVSCEE
AIDAAMKFFDAVDERLKKHCEISKLFVERCLAVSSSAQALDEKQQSVDQRLIEETNTVEELKIRMHETKERMEGARKGIN
VLFHRVDENVPLSDNEIRIFERLKEHQKMLSDMTKLVPKMTLDSNEIHRMANIVLKKRGTGEEQNRFAAVEKNATEIESL
EARENKLNTGISELSI

>**NPP-26$^{\mathrm{GLE1}}$**

MDLVKVDDDVVREMEIRRQQHSKKYDRKFEELVKSMSEAHVEPEKMKIPFSRKPTEQETNNLREEWEKEYAALCTSGRTL
ARPNPNSSIFSKILAQG**S**PSSAKAGTTRSTPRTSKPPSADENIIFGQKLPETPAPKLLCPPRASPGSSRRLSIFVENLP
ENHAEIVRGTIFAGREAKIMQNDPENVPSTSTYGEPIRPYLLFARYISLEKGLLDDKNRYKSATDPRFREFLKENIKYKV
KQSIKHRTSSEACAQILNYFKTLLQKQEVQVLGGYAPVLQLKTNKDIEYASLCIVHNYVILAERDESLIPLISSHITRLS
VFYGYIEQVFCIILLSRSALLRQHPEECMVKFLATSMNPSSPDAIKKWSRQKAHIILFCSVFTKNAYCFSKGFKLKLTDE
ILWKYVDASTAHILEIPRGSFILWQLITTCKPRLQVDKERWIQLLLNIKFKIIPELDLDSFDGDEAMLMQLKQTIDELLH
P

>MEL-28^{ELYS}

MDNENSSIFKSYQGYECWRGEKQIILKDSIGRQLPYIVNFKKNTCQIFDIEWERVTHSFVFPEGCALIDADYFPTEEGKL
GILVGVEDPRQSCGAEHFVLALAVDPDSPAMTITHSLEVPSKITVVKTLFSSADMADETQRTVLKLYHRLMTWQHIVAIG
CKETQCYLARLVAVETPSSPVITVHSEKKYLINLMNAYVSGSVLQYTLDDGAYREYPTAAVYISALSLMPRSR
TLLVGLSMGGILAASLNPSNQMMLLELRHERLVRKIAPLEPEDDPDKFEYFIATVDCSPRHPIMIQLWRGSFKTLEDVDG
EEKYDRPSFSVCLEHKILFGERWLAVNPIVTERDHMMLTRKRGTEDSMHNV**S**QTFGSTSNRNSVLLAYERKKMVIGTED
PNAEPEYIVEAAIFDIDSWYYKRVPGRVSTDGTVLKQCAFLSTIKSNIRSEDVNDIGILTNEATDVSSFSSMVSDADQLF
YPSALSFERVFVAKNTRIDWMKIQNIQDTILNKCAVKLPALIRNPEMISSVVMAAGLVRKNILSGSPNSSAAEI
NELQLSSDQKVLLNVIVYYGKIEEFCQLASRPDISDTLKRELAEWALHEAVDYKRTISDKMVSLFQGRSLALSPLA
EESIAQGIKLFRVVYEYLKACSKALKDDRLRNLAHSVICMRNHTKLTSQFINFAIIPVDPIRQQRMKDLHSKRKNMARKN
SSSLPVQSVVRKMNRQAPNAQFWNDIPHDEWYPPTPLDLLECLLNVSISESIKRELVVQYVIDWISTSPEDSEHSEKQLA
LETIKIMTNQMLNVNLEKIYYILDQGKKALTSSKTSDDMRALGEKVFSMKDDEISYEKLWGKDAPMTVTIGKHDLQRFEQ
RMKMQMEGGKVRLPVLDPESEILYQMFLFENEKFEAMSSEAISSNKLLSAFLPGMIKKDGRGRQKTAKEQEIEISVKKMF
ERKVQNDDEDMPEVFASVNDKTERKRKSSQFGEDDESSVSSSQYVPPTAKRIQQWKSAVESVANNSSINSITSPDSHQNA
EINMMIATPARYYKRHNEEENVQDGFL**S***PAGNRPPPVSAHNSILKTAKGGQSASRGRIRFRADVPRGADE**S**IEDNGRK
GLALNFAILEDEEEETMTIRKSRSMGKHDEEKDSEKNVVDEMEEVKDQEQENDECIESEKTFENQDDFEVLEDTSAPEAA
NTENGSETPPMEDTFEVRDDDVMPPTDETYLSHLQTDKTGILEEEGEDEDIWDGVQR**S**FEVQMDEDCEAVPTIDVADDL
ESKSEEVNEEEVVESEEVQQDAKEPEKTEKRQEEPEPEVMQPVIPEEPQNESLESSIKLQEELQEEPDIVPT*GDEDTAD
KVQEQAVEEDRPP**S***RNTRSSSVQKSTSQVEDRDPKELVEEERPP**S***RN**T***RSASVQKSSNQEKTSESGEVTEEDRPP
SRNTRSASVQKSSSKVKDQKPEELIEEDRPP**S***RN**T**RSASAQKTVAANK**S**VLESEIP**S**RSASRRTRSTSLRNDTVAEP
DETSVAM**T**TRRRTRAT**S***EVVSKQSSEDDGRSTPKTGRTPTKKAAASTSSSRAGSVTRGKKSIIQKMPSPLEVTMEVQE
EEEEEAEEERPASRSTRSASVKNTTVDPSSSALA**S**TKRTTSRKRGN**S***ETIDFNQDDK**S**APT**T**PKRGRPAKKDAGSPK

VGSKARGTKPKSIFENQEDEEDRSS*S*PDIEQPATPTRSSKRTARSRANSESIDDDSKQKTPKKKNAAVNEAGTSKQS
RSVTRSRAS*S*IDVQQEVEEPTTPKRGRGRPPKTVLENIEEGEEERKETAATPLLRSARRAKQ

Data S2: Phosphosites identified in GST-Plk1 PBD Pull-downs

Unambiguously assigned phosphosites are highlighted in yellow and in **bold**

Phosphorylated peptides with unassigned phosphosites are highlighted in yellow

Polo-docking sites are underlined

> NPP-1^{NUP54}, isoform c

MSLFGSSTPQKQAFTFPTPNAPTTSSGTLFGSTTPSKPLFGSTAQASSTPSLFGTTNTSTPSGGLFGKTGTSTTTTST
AGTLFGAAPTTSTATPSLFGASTTGLFGTSSTTSGGLGGIGSTQQTAKSPVATRLAAFKSGTLGAGSLSGNTPTSFATPS
ALPGTNAPQSSFLSPANNLNVAPAAYRPPYSTFGGSTPFGAASTGTAAGSTLFGSSTAKPATGGFFGSSSGSTLGGLGAT
QQQQQPVVQQQQVIQQYHPFVKAVGDPKLFGNDNDGVVAKLNQVAAGLGVGKAPYKDGNQLLSFSMEGNLFERFVGIGYN
RISERTDDEGFVTLVLRHPITNLNTEERRDKILEIIKAILGGGPNVEVRYAPGTSMRTLSDGCTEICIIAKEGGFVAGAI
KLAQILNDAPKMTQLESQLQVDKTRVLPKVGMSKAQRDRYLETVPDGIDERIWRQAIKENPAPNKLLPVPVRGWEALRDR
QKAQVGESKLFHEAINALGNRVEEANHEHADAVVKMEIIRNRHKTLSYRIVRVMLAQWIVSRYSRQIDTDEDVIEAKADT
LLAQMNRHNQVKFYVDKFYEILESKPDKLQESMWKMFDMTIEDEHYARRVLTKFVNICSGLYESTHQQIESLEACRRALE
G

>NPP-7^{NUP153}

MSDKSGGFFSSVGRFFSVGASATKSKDGDKSNDEGTSNSSSKSSPSASPALKNILSVDNEAKIASDETSVNT**S**
QNHRIGSRLRYL**S**PTALDRRTSASNGLEIDVDAVPDIFFSTPAVSTNRKRQLTELDKQILPQSFRSESVKRSRFLNRSL
LEPTLNSSMNNERKMDMTWCGELTANHSPASSITNFSLLSRRSGATTNGSLSTRTQEIFKKLEGAN**T**PAKEVQRMSMLR
AGIARPEKWGSFSESKVPNGSATGTPPPPLKKAGDAIPSRIQLISKTMGMSARRAPYWTDLTRKRTSSKNGDTGSSDSMK
SLNGNFATAELSSLFSLDLPAPKKTASTASTTTINSSNSRKQHASIMKGPDGKPVSRNTFKLSDDIEEVDEDSQKLPPIP
TTTLQNPQPLKLAPEYAPKRGFLDDLAFSFTAPVDLVTAVGTAKTASTASSHKASESSSESNAESTEKQTSESSGNESDS
EESEDSEVDVGEENGSEVKESPQTSADTISSAGGSNQSSKDSSPKVAKDAEPVVVAPAPVVDAGSKKWECQSCFCSWDST
LSECGACGEARPGGSGTGPKSQPKPSEKQLVSNLSSFASN**T**PSTVKFGFGSGASTTTTIASTTSNTIPFGSGSSVAPLF
GAPKTTAPPPTTVPATIPVAPTTIASAPVAAVTSSSNGTRVDWECPDCMVSNKASDDKCPCCSHVKYASAEASSNVFGNR
AFKPLSSTGSTISFGVGGSTATTQPSAFAFGLSKTTEVAPTSTPAFGLSAKPAVSSAPVEKPSAPEVPKTAGSLFGNIAK
PADSATTSFLAPGAASSTSSLAPTAGSSLFGGSTGSIFNLNKTNTTETAKPGLFGSILDKETHVTTTPVSVALPSSTDST
QSKSAIPTIAPTMSLFGNSSTGSFGGSSMFGNSKTELPKISSTLSFGNPTTAAAPVSTATAEAVKTNVFGSNSASASTSL
FGAGSSSTATNLFTTKPADSTTSSIFGKPISFGDDSGTTTTDGAPAKRGLFSSDSQKLQFGGQQKVEMPKFTGFGNPAST
ASTTSGSLFGGASTNPPMFGGPSSSSIPAFSTSNSSSTSGFPSSTATPFGNAGTTSTSGVFGAFGNKPSQPGLSSSSSTN
SLFGQAPADSSNPFGGTSNNGFNFGASSSTTGATAGGGGVFQFGNAATSAPAPTAAPGGGAFQFGGNMSVPQAPAPGGME
NAFSYQAPSGVGARKMAMARRRNMRK

>NPP-10N^{NUP98}, isoform b

MFGQNKSFGSSSFGGGSSGSGLFGQNNQNNQNKGLFGQPANNSGTTGLFGAAQNKPAGSIFGAASNTSSIFGSPQQPQNN
QSSLFGGGQNNANRSIFGSTSSAAPASSSLFGNNANNTGTSSIFGSNNNAPSGGGLFGASTVSGTTVKFEPPISSDTMMR
NGTTQTISTKHMCISAMSKYDGKSIEELRVEDYIANRKAPGTGTTSTGGGLFGASNTTNQAGSSGLFGSSNAQQKTSLFG
GASTSSPFGGNTSTANTGSSLFGNNNANTSAASGSLFGAKPAGSSLFGSTATTGASTFGQTTGSSLFGNQQPQTNTGGSL
FGNTQNQNQSGSLFGNTGTTGTGLFGQAQQQPQQQSSGFSFGGAPAATNAFGQPAAANTGGSLFGNTSTANTGSSLFGAK
PATSTGFTFGATQPTTTNAFGSTNTGGGLFGNNAAKPGGLFGNTTNTGTGGGLFGSQPQASSGGLFGSNTQATQPLNTGF
GNLAQPQIVMQQQVAPVPVIGVTADVLQMQANMKSLKSQLTNAPYGD**S**PLLKYNANPEIDGKS**S**PASTQRQLRFLAAKK
GALSSSSDAQDS**S**FIIPPISKVMSDL**S**PAVTR**S**ADVTKDLNYTSKEAPP**S**LARGLRNSTFNPNMSLTNRSVHESSALD
KTIDSALDA**S**MNGTSNRLGVRG**S**VRRSNLKQLDM**S**LLADSSRVGRESRVADPDALPRI**S**ESERRQDVVTS**T**PAVDPVQ
AVIQRHNDRNRDPPSLNLDTTCDEHTGLEPVSAATSSAASVVSTPSEETVNVNSAAGVKLTKPDYFSLPTINEMKNMIKN
GRVVLEDGLTVGRSSYGSVYWPGRVELKDVALDEIVVFRHREVTVYPNEEEKAPEGQELNRPAEVTLERVWYTDKKTKKE
VRDVVKLSEIGWREHLERQTIRMGAAFKDFRAETGSWVFRVDHFS

>NPP-12^{gp210}

MIILRSLLVLALVQITISYRLNVPRVLLPYHPTVPVSFVLEVTHPTGGCFTWRSTRPDIVSVKRIETNEAGCSDKAEIRS
VAKPGTVGSSELSAVIFAEDKGSGTTLSCGVTVDEIATISIETTTKVLFVDAAPARMTVDAFNADGDRFSTLSEIALEWE
LASTSSNKAKPLRIVPFEQSTYEAPSEIVKLEKNRKKGYLILIEGVGTGTATLTTKFSDAYLQKVAAHNVELAVVANLLL

VPSQDVYLPVHSVLPFQVLIVKQRGTEIVNMPNPSYELQIDGGDVASLDKKSSSVRALTIGNTAVHLLSSHVDVRAKAGL
RPPSTVIHVVDAESVQWHVSGDNWMLETGKQYTINVELLDEHGNVMFVADNSRFDTHIDEQFLHVDFKSENGTWFLVTPL
KPSKTTLRTKFVAIIDAKGNRIAQSGKIGGEQRVTIVDPVRIVPPVIYLPFVSEKRSQIDLTATGGSGLFEWTSEDGHVA
TVDLLTGRMTANSLGSTKVKATDKRNDQLRDIASVHILEVSGIGFGETVRETFVGDTLTLNIKATGLTSDGLLVEMSDCR
NIRAHVQITDNALLRHESSADSSLPMMGTGCGTITFKGLSSGDARVSISYLGHKASIDVAVYEKLSISEESSSIALGSTH
PLTVSGGPRPWILDPANFYKTQETKQSQLQVTFENEKVLFKCGSSEVTEAVRLRIGNLKSSTLPLPIHSEITVSICCAKP
TRLEIFDKKQRPSKCPLNVHSMLINTNVELVLRGSGVCNGAATPLASINGLSPKWTTSDSGLLTVNRHGIEADATSGKKE
GQVTIQAQAGSLSTKYEITVKKGLNVEPARLVLWNEAVSKGTFTITGGSGHFHVDNLPTSDSPVAIALRARSLTVTPKNN
GQVNLRISDACLVGQHADASVRIADIHSLAIDAPQFVEIGQEVEVEILAQDETGASFEKEHRPLADAQLDASNNHVILTK
VDGLRYTLRANSIGTVSLSASSKSSSGRVLSSRPHTVQIFSPIFLQPKRLTLIPDSKFQLEVVGGPQPTPPLDFSLNNSM
IASIEPNALITSSELGYTAITGTVRVGDGHVTLDTVVLRVASLGGIILSASSRKVETGGRVNLRLRGVIAGAEDEEPFAF
GGAIYPFKVTWSVSDPSVLFTTHPLGGDVVEPTDNQFAIWFNAIRGGSVTVKAVVELNEKARKHFTGRTSTFTAETTITV
EDGLSLVQPEMDINTVRVAPNSQLKMVTAWSQASFSVPSDFSSRIVISADGHLITNGKEGSAAITVRNVNSPDNETVLIP
VTVSRVASLDVHPTIELKSAFENSPLIHLPVGAQIQLNVVPRDARGRRLAAASNSINFRPHRFDLTDIVATNSNQTLTIT
LKTAGDTVLRIGDASNTHIATFIRLSASESILPRAAHKYANDLVVSDVICLQSNIFTVDGSRWSSQSSSEGRISWLDENL
GVAQLTKAGNTFIRLHADKQTIHSKISVVLPSSLRFPDGQKPEFVSNDEHSVFVIPVIAATNNTSGSKVSSIYGECTADQ
IRSFDAIGAPFECQVAFTRRSKIISAVNWLTVSAVFSPVFGYGCEIRRFDSSVSTSSIVVPEELLKDQFDARITAKWISD
GTVQVNDATIDVPFHFAFIVEEKELVFSNMNQIEAALSIWAPTYDSKHIVVSGCEGDIVSVEKTSRSSDKHSAKANVFYN
IRLNIKSAALFTEHAKKCQISVENTLTGQVIRVPVTVQLLDETAKQVYNALESRGVVDVLLILAHKYSHAIPTLLWTCLV
GIIILVIGIYVKMNVFDKTGSFGDNTLNNTTHQTSMASSLSSTNVSLREPVFRSTPIAG**S**PQVSLPTARDRLRNQMGSG
GDNRLWSY

>NPP-14^{NUP214}
MSNEDVAEDVSQVTDFHFHTCRKFRLFSSKSDGYSQNEINIRNRVSQLGVTFVTVNSNQLSCFHTKSLLGYKITRENMNV
EVTDLPIKTIRLHGVVLINDMGPSSSLFSASVNSDGTVLGVLHTKNNDVSVDVFDIKKICTSSSIEPFKPLCTTRVGTEQ
INQGSCLEWNPAFPDTFAASSTDRSILVAKINVQSPANQKLVGIGKFGAVTTAISWSPKGKQLTIGDSLGKIVQLKPELE
VVRSQHGPENKPNYGRITGLCWLATTEWLVSLENGTDQDAYLMRCKKDKPTEWIQFHELSYSSSKWPLPPQLFPATQLLV
DWNVVIVGNSKTSEISTVGKRDDWQTWVPVEGESIYLPTTSSGKDTVPIGVAVDRSMTDEVLLNPDGSQRHRPSPLVLCL
TNDGILTAHHIISTFAAHIPCQMSSQNLAINDLKKLQFDSQKPISAPPSDQTPVTKPSTVFGQKPEAETLKSSLVGSPSS
VQTPKPSSSLFNPKSIASNIETSQL**T**ESKPSTPAAPSSQPKIAS**T**PK**S**EAIPKISDKTLEHKKAELIATKKQVLIERMD
KINDSMAGAKDATMKLSFAVGKVKTTIMECADVVRASLGDSKEVMDELKNLILSIERMSDRTQHTVKEMDFEIDEKMELV
AGVEDGNQVLEKLRNMSETEKLMRFNKLETAADLLNGKYEECSDLIKKLRMSLSEKESLRKQAIL**S**PLRLSSNLNQLRS
GSETELALKVMRNVSKIIMDTREQIQRTELEFVRFQRDVKFQNFKKGKENLNFTQPLEMSSLDGDAPQGKSLTDAESIKV
RQALVNQIQKRGIVKTRNVIVESYKKSENSAAMKNDLLDTSNLSNAILKLSM**T**PRRVMPSSSLFSA**S**PS**T**PSTKSDAAT
QADEPPIVKTVVVTVESPAKPIASAPAVSSPLIKLNTTTATTTMT**T**PKV**T**VPKEEANKTQDQKPIISTPASSSIFSSGS
LFGTKTQTPLVSKEESTLTTGVPSLINSSLSISPQEIEKASSKVETLNKTEEVKDEKSENEVTPDLKSEEPKSLETKVKE
EPKPAVQTPVKEEETGSNIQKTPSFSFNSTT**T**PKSTSST**SS**IFGGGLKTQ**T**PSSSNSTNIFGARTTTTA**T**PTPASN
TSSIFGGGSKAASSPFGSFGQAGCQPAKTSNPATSTASVTFSFNTGATSASAKPAGFGSFGAGASAKPSSVFGGSV
TAPTVPNVDDGMEDDSMANGGGSGGFMSGLGNARTSNTSGGNNPFAPKTSTGTSASSSSWLFGGGGNQQQQQQKPSFSF
NTAGSSAQQASAPATGTSSVFGGAPKFGSQPAFGAKPFGGGANAGLSKNASIFGGATSSTTNNPATGGFAQFASGQKTSS
LFGGGATPQTNTSIFGGGANTTPAPTSSVFGGGASANANKPTSFTSWR

>NPP-19^{NUP53} **isoform b**
MFSHLNQN**T**SGRHNSMDLNNSSISNFGTPVEQSTPALLFGKRKATVPSSYTASPLNTASAPCSDIFAVSAPAVPQHLKD
TPGSKSVHWSPSLVQSGEKSAAQTQNTPANLSFGGNSSFSAPTKPAPQSIQTSSFGGQAMHAPPLRSLRDKVEPAKKISR
RNTFTARS**T**PLS**T**PITQRVTSRLAEAEEQPMEEEADAADTWVTVFGFQPSQVSILLNLFSRHGEVVSHQTPSKGNFIHM
RYSCVTHAQQAISRNGTLLDQDTFIGVVQCTNKDVINGSASGIVARSSNIAAAANRSASMYNSFVENDMADQSVNHNENS
VLNSSNVFDANNSLNSSRISVR**S**GVGMRPLAADQRTNILQG**T**PSVRKAPDGLLNKFWNTIGLN

>NPP-24^{NUP88}
MLITHLIDSLSDRAVITPLTPTSVIIYDKNELKVYVGFTNNRNSLEYTKEVVLLLSTEIPKKAEIREIIVSKNGDYVILE
GPRSLFVVRIGAEILVAKPDRLPSECFCECYPLHDSLLLQNISLSVVKVRLLPEKCDEKTFVTAVLFSDNCIRFYNLQKK
FDSLLLAVDFRNHLHQVHDENVANN**T**FGLQKALVSFDLIPPKPNTSHFSIISIDSDCDFYTSFVHFSCFKEGYAPRIHR
IEPVDGLPCDPLDLRYIQTTNPRICSVFVLVSGGGVLSHLVVFPNEFGEFRFLVKDQLRLPSSNGDPRIVQNQIRSLKVS
RYEIATSSSLFSVNIFPWFEALTSISPTSTLEKETRVSELVDAVIPSDELSNTTKWTGARALRAVSVQLTQSLATEEEEL

LPESENIMHLVILENKDGQPAHLFNISTFDNIWSTENKTSFGRDSVSQPPMKSTGSLEQQLAALKPLAACVISEKVSCEE
AIDAAMKFFDAVDERLKKHCEISKLFVERCLAVSSSAQALDEKQQSVDQRLIEETNTVEELKIRMHETKERMEGARKGIN
VLFHRVDENVPLSDNEIRIFERLKEHQKMLSDMTKLVPKMTLDSNEIHRMANIVLKKRGTGEEQNRFAAVEKNATEIESL
EARENKLNTGISELSI

>MEL-28^{ELYS}

MDNENSSIFKSYQGYECWRGEKQIILKDSIGRQLPYIVNFKKNTCQIFDIEWERVTHSFVFPEGCALIDADYFPTEEGKL
GILVGVEDPRQSCGAEHFVLALAVDPDSPAMTITHSLEVPSKITVVKTLFSSADMADETQRTVLKLYHRLMTWQHIVAIG
CKETQCYLARLVAVETPSSPVITVHSEKKYLINLMNAYVSGSVLQYTLDDGAYREYPTAAVYISALSLMPRSRTLLVGLS
MGGILAASLNPSNQMMLLELRHERLVRKIAPLEPEDDPDKFEYFIATVDCSPRHPIMIQLWRGSFKTLEDVDGEEKYDRP
SFSVCLEHKILFGERWLAVNPIVTERDHMMLTRKRGTEDSMHNVSQTFGSTSNRNSVLLAYERKKMVIGTEDPNAEPEYI
VEAAIFDIDSWYYKRVPGRVSTDGTVLKQCAFLSTIKSNIRSEDVNDIGILTNEATDVSSFSSMVSDADQLFYPSALSFE
RVFVAKNTRIDWMKIQNIQDTILNKCAVKLPALIRNPEMISSVVMAAGLVRKNILSGSPNSSAAEINELQLSSDQKVLLN
VIVYYGKIEEFCQLASRPDISDTLKRELAEWALHEAVDYKRTISDKMVSLFQGRSLALSPLAEESIAQGIKLFRVVYEYL
KACSKALKDDRLRNLAHSVICMRNHTKLTSQFINFAIIPVDPIRQQRMKDLHSKRKNMARKNSSSLPVQSVVRKMNRQAP
NAQFWNDIPHDEWYPPTPLDLLECLLNVSISESIKRELVVQYVIDWISTSPEDSEHSEKQLALETIKIMTNQMLNVNLEK
IYYILDQGKKALTSSKTSDDMRALGEKVFSMKDDEISYEKLWGKDAPMTVTIGKHDLQRFEQRMKMQMEGGKVRLPVLDP
ESEILYQMFLFENEKFEAMSSEAISSNKLLSAFLPGMIKKDGRGRQKTAKEQEIEISVKKMFERKVQNDDEDMPEVFASV
NDKTERKRKSSQFGEDDESSVSSSQYVPPTAKRIQQWKSAVESVANNSSINSITSPDSHQNAEINMMIATPARYYKRHNE
EENVQDGFLSPAGNRPPPVSAHNSILKTAKGGQSASRGRIRFRADVPRGADE**S**IEDNGRKGLALNFAILEDEEEETMTI
RKSRSMGKHDEEKDSEKNVVDEMEEVKDQEQENDECIESEKTFENQDDFEVLEDTSAPEAANTENGSETPPMEDTFEVRD
DDVMPPTDETYLSHLQTDKTGILEEEGEDEDIWDGVQRSFEVQMDEDCEAVPTIDVADDLESKSEEVNEEEVVESEEVQQ
DAKEPEKTEKRQEEPEPEVMQPVIPEEPQNESLESSIKLQEELQEEPDIVPTGDEDTADKVQEQAVEEDRPPSRNTRSSS
VQKSTSQVEDRDPKELVEEERPPSRNTRSASVQKSSNQEKTSESGEVTEEDRPPSRNTRSASVQKSSSKVKD
QKPEELIEEDRPPSRNTRSASAQKTVAANKSVLESEIPSRSASRRTRSTSLRNDTVAEPDETSVAMTTRRR
TRAT**S**EVVSKQSSEDDGRSTPKTGRTPTKKAAASTSSSRAGSVTRGKKSIIQKMPSPLEVTMEVQEEEEEEAEEERPAS
RSTRSASVKNTTVDPSSSALA**S**TKRTTSRKRGNSETIDFNQDDKSAPTTPKRGRPAKKDAGSPKVGSKARGTKPKSIF
ENQEDEEDRSSSPDIEQPATPTRSSKRTARSRANSESIDDDSKQK**T**PKKKNAAVNEAGTSKQSRSVTRSR
ASSIDVQQEVEEPT**T**PKRGRGRPPKTVLENIEEGEEERKETAATPLLRSARRAKQ

Table S4, (related to Figure 4): LC-MS/MS table presenting the phosphopeptides identified on nucleoporins from embryonic extracts.

Table S5 (related to Figure 4): Summary table presenting the nucleoporins significantly enriched on GST-PBD pull-downs ($p<0.05$).

Table S6 (related to Figure 4): LC-MS/MS table presenting the phosphopeptides identified on nucleoporins specifically retained on the GST-PBD.

__Macro generated in Fiji to convert .tif movie to an image sequence of channel 1 compatible with Ilastik and quantify the integral density value at the nuclear envelope.__

```
roiManager("reset");
run("Options...", "iterations=1 count=1 black do=Nothing");
run("Set Measurements...", "area mean integrated stack display redirect=None decimal=3");
run("Colors...", "foreground=white background=black selection=yellow");
close("*");
run("Image Sequence...");
imagesList = getList("image.titles");
level = getNumber("Threshold between O and 1: ",0.20);
for (i=0; i<imagesList.length-1; i++){Berg et al., 2019, #88830}agesList[i], "Probabilities.tif") ==
true)
	{print("	"+imagesList[i]);
		selectImage(imagesList[i]);
		run("Duplicate...", "duplicate channels=1");
		setThreshold(level, 1000000000000000000000000000000.0000);
		setOption("BlackBackground", true);
		run("Convert to Mask");
selectImage(imagesList[i]);close();
}
}
run("Images to Stack", "name=Stack title=[Probabilities] use");
//run("Invert LUT");
for (i=0; i<imagesList.length-1; i++){Berg et al., 2019, #88830}af0 setSlice(i+1);
	run("Create Selection");
//	run("Make Inverse");
	RoiExist = getValue("selection.size");
	if (RoiExist > 0)
	{
		roiManager("Add");
		roiManager("Select", i);
//		roiManager("Rename", i);
	}
```

REFERENCES AND NOTES

1. L. Champion, M. I. Linder, U. Kutay, Cellular reorganization during mitotic entry. *Trends Cell Biol.* **27**, 26–41 (2017).

2. P. De Magistris, W. Antonin, The dynamic nature of the nuclear envelope. *Curr. Biol.* **28**, R487–R497 (2018).

3. B. Hampoelz, A. Andres-Pons, P. Kastritis, M. Beck, Structure and assembly of the nuclear pore complex. *Annu. Rev. Biophys.* **48**, 515–536 (2019).

4. D. H. Lin, A. Hoelz, The structure of the nuclear pore complex (an update). *Annu. Rev. Biochem.* **88**, 725–783 (2019).

5. E. Dultz, M. Wojtynek, O. Medalia, E. Onischenko, The nuclear pore complex: Birth, life, and death of a cellular Behemoth. *Cell* **11**, 1456 (2022).

6. B. B. Hülsmann, A. A. Labokha, D. Görlich, The permeability of reconstituted nuclear pores provides direct evidence for the selective phase model. *Cell* **150**, 738–751 (2012).

7. H. B. Schmidt, D. Görlich, Transport selectivity of nuclear pores, phase separation, and membraneless organelles. *Trends Biochem. Sci.* **41**, 46–61 (2016).

8. U. Kutay, R. Jühlen, W. Antonin, Mitotic disassembly and reassembly of nuclear pore complexes. *Trends Cell Biol.* **31**, 1019–1033 (2021).

9. E. Laurell, K. Beck, K. Krupina, G. Theerthagiri, B. Bodenmiller, P. Horvath, R. Aebersold, W. Antonin, U. Kutay, Phosphorylation of Nup98 by multiple kinases is crucial for NPC disassembly during mitotic entry. *Cell* **144**, 539–550 (2011).

10. M. I. Linder, M. Köhler, P. Boersema, M. Weberruss, C. Wandke, J. Marino, C. Ashiono, P. Picotti, W. Antonin, U. Kutay, Mitotic disassembly of nuclear pore complexes involves CDK1- and PLK1-mediated phosphorylation of key interconnecting nucleoporins. *Dev. Cell* **43**, 141–156.e7 (2017).

11. E. Dultz, E. Zanin, C. Wurzenberger, M. Braun, G. Rabut, L. Sironi, J. Ellenberg, Systematic kinetic analysis of mitotic dis- and reassembly of the nuclear pore in living cells. *J. Cell Biol.* **180**, 857–865 (2008).

12. H. Chug, S. Trakhanov, B. B. Hülsmann, T. Pleiner, D. Görlich, Crystal structure of the metazoan Nup62•Nup58•Nup54 nucleoporin complex. *Science* **350**, 106–110 (2015).

13. M. Heusel, M. Frank, M. Köhler, S. Amon, F. Frommelt, G. Rosenberger, I. Bludau, S. Aulakh, M. I. Linder, Y. Liu, B. C. Collins, M. Gstaiger, U. Kutay, R. Aebersold, A global screen for assembly state changes of the mitotic proteome by SEC-SWATH-MS. *Cell Syst.* **10**, 133–155.e6 (2020).

14. L. Pintard, B. Bowerman, Mitotic cell division in *Caenorhabditis elegans*. *Genetics* **211**, 35–73 (2019).

15. O. Cohen-Fix, P. Askjaer, Cell biology of the *Caenorhabditis elegans* nucleus. *Genetics* **205**, 25–59 (2017).
16. J. Liu, T. Rolef Ben-Shahar, D. Riemer, M. Treinin, P. Spann, K. Weber, A. Fire, Y. Gruenbaum, Essential roles for *Caenorhabditis elegans* lamin gene in nuclear organization, cell cycle progression, and spatial organization of nuclear pore complexes. *Mol. Biol. Cell* **11**, 3937–3947 (2000).
17. V. Galy, I. W. Mattaj, P. Askjaer, *Caenorhabditis elegans* nucleoporins Nup93 and Nup205 determine the limit of nuclear pore complex size exclusion in vivo. *Mol. Biol. Cell* **14**, 5104–5115 (2003).
18. K. K. Lee, Y. Gruenbaum, P. Spann, J. Liu, K. L. Wilson, *C. elegans* nuclear envelope proteins emerin, MAN1, lamin, and nucleoporins reveal unique timing of nuclear envelope breakdown during mitosis. *Mol. Biol. Cell* **11**, 3089–3099 (2000).
19. V. Hachet, C. Busso, M. Toya, A. Sugimoto, P. Askjaer, P. Gonczy, The nucleoporin Nup205/NPP-3 is lost near centrosomes at mitotic onset and can modulate the timing of this process in *Caenorhabditis elegans* embryos. *Mol. Biol. Cell* **23**, 3111–3121 (2012).
20. L. Martino, S. Morchoisne-Bolhy, D. K. Cheerambathur, L. Van Hove, J. Dumont, N. Joly, A. Desai, V. Doye, L. Pintard, Channel nucleoporins recruit PLK-1 to nuclear pore complexes to direct nuclear envelope breakdown in *C. elegans*. *Dev. Cell* **43**, 157–171.e7 (2017).
21. G. Velez-Aguilera, B. Ossareh-Nazari, L. Van Hove, N. Joly, L. Pintard, Cortical microtubule pulling forces contribute to the union of the parental genomes in the *Caenorhabditis elegans* zygote. *eLife* **11**, e75382 (2022).
22. G. Velez-Aguilera, S. Nkombo Nkoula, B. Ossareh-Nazari, J. Link, D. Paouneskou, L. Van Hove, N. Joly, N. Tavernier, J. M. Verbavatz, V. Jantsch, L. Pintard, PLK-1 promotes the merger of the parental genome into a single nucleus by triggering lamina disassembly. *eLife* **9**, e59510 (2020).
23. M. Rahman, I. Y. Chang, A. Harned, R. Maheshwari, K. Amoateng, K. Narayan, O. Cohen-Fix, *C. elegans* pronuclei fuse after fertilization through a novel membrane structure. *J. Cell Biol.* **219**, e201909137 (2020).
24. A. Audhya, A. Desai, K. Oegema, A role for Rab5 in structuring the endoplasmic reticulum. *J. Cell Biol.* **178**, 43–56 (2007).
25. V. Galy, W. Antonin, A. Jaedicke, M. Sachse, R. Santarella, U. Haselmann, I. Mattaj, A role for gp210 in mitotic nuclear-envelope breakdown. *J. Cell Sci.* **121**, 317–328 (2008).
26. N. Portier, A. Audhya, P. S. Maddox, R. A. Green, A. Dammermann, A. Desai, K. Oegema, A microtubule-independent role for centrosomes and Aurora A in nuclear envelope breakdown. *Dev. Cell* **12**, 515–529 (2007).

27. D. Chase, C. Serafinas, N. Ashcroft, M. Kosinski, D. Longo, D. K. Ferris, A. Golden, The Polo-like kinase PLK-1 is required for nuclear envelope breakdown and the completion of meiosis in *Caenorhabditis elegans*. *Genesis* **26**, 26–41 (2000).
28. M. M. Rahman, M. Munzig, K. Kaneshiro, B. Lee, S. Strome, T. Müller-Reichert, O. Cohen-Fix, C. *Caenorhabditis elegans* Polo-like kinase PLK-1 is required for merging parental genomes into a single nucleus. *Mol. Biol. Cell* **26**, 4718–4735 (2015).
29. A. E. Elia, L. C. Cantley, M. B. Yaffe, Proteomic screen finds pSer/pThr-binding domain localizing Plk1 to mitotic substrates. *Science* **299**, 1228–1231 (2003).
30. A. E. Elia, P. Rellos, L. F. Haire, J. W. Chao, F. J. Ivins, K. Hoepker, D. Mohammad, L. C. Cantley, S. J. Smerdon, M. B. Yaffe, The molecular basis for phosphodependent substrate targeting and regulation of Plks by the Polo-box domain. *Cell* **115**, 83–95 (2003).
31. M. Boxem, Z. Maliga, N. Klitgord, N. Li, I. Lemmens, M. Mana, L. de Lichtervelde, J. D. Mul, D. van de Peut, M. Devos, N. Simonis, M. A. Yildirim, M. Cokol, H. L. Kao, A. S. de Smet, H. Wang, A. L. Schlaitz, T. Hao, S. Milstein, C. Fan, M. Tipsword, K. Drew, M. Galli, K. Rhrissorrakrai, D. Drechsel, D. Koller, F. P. Roth, L. M. Iakoucheva, A. K. Dunker, R. Bonneau, K. C. Gunsalus, D. E. Hill, F. Piano, J. Tavernier, S. van den Heuvel, A. A. Hyman, M. Vidal, A protein domain-based interactome network for *C. elegans* early embryogenesis. *Cell* **134**, 534–545 (2008).
32. J. Link, D. Paouneskou, M. Velkova, A. Daryabeigi, T. Laos, S. Labella, C. Barroso, S. P. Piñol, A. Montoya, H. Kramer, A. Woglar, A. Baudrimont, S. M. Markert, C. Stigloher, E. Martinez-Perez, A. Dammermann, M. Alsheimer, M. Zetka, V. Jantsch, Transient and partial nuclear lamina disruption promotes chromosome movement in early meiotic prophase. *Dev. Cell* **45**, 212–225.e7 (2018).
33. Y. C. Lussi, I. Hügi, E. Laurell, U. Kutay, B. Fahrenkrog, The nucleoporin Nup88 is interacting with nuclear lamin A. *Mol. Biol. Cell* **22**, 1080–1090 (2011).
34. T. Al-Haboubi, D. K. Shumaker, J. Köser, M. Wehnert, B. Fahrenkrog, Distinct association of the nuclear pore protein Nup153 with A- and B-type lamins. *Nucleus* **2**, 500–509 (2011).
35. L. A. Hawryluk-Gara, E. K. Shibuya, R. W. Wozniak, Vertebrate Nup53 interacts with the nuclear lamina and is required for the assembly of a Nup93-containing complex. *Mol. Biol. Cell* **16**, 2382–2394 (2005).
36. M. S. Mauro, G. Celma, V. Zimyanin, M. M. Magaj, K. H. Gibson, S. Redemann, S. Bahmanyar, Ndc1 drives nuclear pore complex assembly independent of membrane biogenesis to promote nuclear formation and growth. *eLife* **11**, e75513 (2022).
37. R. Maheshwari, M. M. Rahman, S. Drey, M. Onyundo, G. Fabig, M. A. Q. Martinez, D. Q. Matus, T. Müller-Reichert, O. Cohen-Fix, A membrane reticulum, the centriculum, affects centrosome size and function in *Caenorhabditis elegans*. *Curr. Biol.* **33**, 791–806.e7 (2023).

38. J. P. Aris, G. Blobel, Yeast nuclear envelope proteins cross react with an antibody against mammalian pore complex proteins. *J. Cell Biol.* **108**, 2059–2067 (1989).
39. E. Ródenas, E. P. Klerkx, C. Ayuso, A. Audhya, P. Askjaer, Early embryonic requirement for nucleoporin Nup35/NPP-19 in nuclear assembly. *Dev. Biol.* **327**, 399–409 (2009).
40. L. A. Hawryluk-Gara, M. Platani, R. Santarella, R. W. Wozniak, I. W. Mattaj, Nup53 is required for nuclear envelope and nuclear pore complex assembly. *Mol. Biol. Cell* **19**, 1753–1762 (2008).
41. J. Fischer, R. Teimer, S. Amlacher, R. Kunze, E. Hurt, Linker Nups connect the nuclear pore complex inner ring with the outer ring and transport channel. *Nat. Struct. Mol. Biol.* **22**, 774–781 (2015).
42. J. Kosinski, S. Mosalaganti, A. von Appen, R. Teimer, A. L. DiGuilio, W. Wan, K. H. Bui, W. J. Hagen, J. A. Briggs, J. S. Glavy, E. Hurt, M. Beck, Molecular architecture of the inner ring scaffold of the human nuclear pore complex. *Science* **352**, 363–365 (2016).
43. D. H. Lin, T. Stuwe, S. Schilbach, E. J. Rundlet, T. Perriches, G. Mobbs, Y. Fan, K. Thierbach, F. M. Huber, L. N. Collins, A. M. Davenport, Y. E. Jeon, A. Hoelz, Architecture of the symmetric core of the nuclear pore. *Science* **352**, eaaf1015 (2016).
44. S. Petrovic, D. Samanta, T. Perriches, C. J. Bley, K. Thierbach, B. Brown, S. Nie, G. W. Mobbs, T. A. Stevens, X. Liu, G. P. Tomaleri, L. Schaus, A. Hoelz, Architecture of the linker-scaffold in the nuclear pore. *Science* **376**, eabm9798 (2022).
45. D. M. Rivers, S. Moreno, M. Abraham, J. Ahringer, PAR proteins direct asymmetry of the cell cycle regulators Polo-like kinase and Cdc25. *J. Cell Biol.* **180**, 877–885 (2008).
46. Y. Nishi, E. Rogers, S. M. Robertson, R. Lin, Polo kinases regulate *C. elegans* embryonic polarity via binding to DYRK2-primed MEX-5 and MEX-6. *Development* **135**, 687–697 (2008).
47. Y. Budirahardja, P. Gonczy, PLK-1 asymmetry contributes to asynchronous cell division of *C. elegans* embryos. *Development* **135**, 1303–1313 (2008).
48. N. Tavernier, A. Noatynska, C. Panbianco, L. Martino, L. Van Hove, F. Schwager, T. Léger, M. Gotta, L. Pintard, Cdk1 phosphorylates SPAT-1/Bora to trigger PLK-1 activation and drive mitotic entry in *C. elegans* embryos. *J. Cell Biol.* **208**, 661–669 (2015).
49. B. Han, K. R. Antkowiak, X. Fan, M. Rutigliano, S. P. Ryder, E. E. Griffin, Polo-like kinase couples cytoplasmic protein gradients in the *C. elegans* zygote. *Curr. Biol.* **28**, 60–69.e8 (2018).
50. A. Noatynska, C. Panbianco, M. Gotta, SPAT-1/Bora acts with Polo-like kinase 1 to regulate PAR polarity and cell cycle progression. *Development* **137**, 3315–3325 (2010).
51. A. J. Kim, E. E. Griffin, PLK-1 regulation of asymmetric cell division in the early *C. elegans* embryo. *Front Cell Dev. Biol.* **8**, 632253 (2020).
52. C. J. Bley, S. Nie, G. W. Mobbs, S. Petrovic, A. T. Gres, X. Liu, S. Mukherjee, S. Harvey, F. M. Huber, D. H. Lin, B. Brown, A. W. Tang, E. J. Rundlet, A. R. Correia, S. Chen, S. G. Regmi, T. A.

Stevens, C. A. Jette, M. Dasso, A. Patke, A. F. Palazzo, A. A. Kossiakoff, A. Hoelz, Architecture of the cytoplasmic face of the nuclear pore. *Science* **376**, eabm9129 (2022).

53. K. Y. Cheng, E. D. Lowe, J. Sinclair, E. A. Nigg, L. N. Johnson, The crystal structure of the human Polo-like kinase-1 polo box domain and its phospho-peptide complex. *EMBO J.* **22**, 5757–5768 (2003).

54. J. E. Park, N. K. Soung, Y. Johmura, Y. H. Kang, C. Liao, K. H. Lee, C. H. Park, M. C. Nicklaus, K. S. Lee, Polo-box domain: A versatile mediator of Polo-like kinase function. *Cell. Mol. Life Sci.* **67**, 1957–1970 (2010).

55. V. Archambault, G. Lépine, D. Kachaner, Understanding the Polo kinase machine. *Oncogene* **34**, 4799–4807 (2015).

56. F. Gnad, J. Gunawardena, M. Mann, PHOSIDA 2011: The posttranslational modification database. *Nucleic Acids Res.* **39**, D253–D260 (2011).

57. C. Franz, R. Walczak, S. Yavuz, R. Santarella, M. Gentzel, P. Askjaer, V. Galy, M. Hetzer, I. W. Mattaj, W. Antonin, MEL-28/ELYS is required for the recruitment of nucleoporins to chromatin and postmitotic nuclear pore complex assembly. *EMBO Rep.* **8**, 165–172 (2007).

58. V. Galy, P. Askjaer, C. Franz, C. López-Iglesias, I. W. Mattaj, MEL-28, a novel nuclear-envelope and kinetochore protein essential for zygotic nuclear-envelope assembly in *C. elegans*. *Curr. Biol.* **16**, 1748–1756 (2006).

59. G. Gómez-Saldivar, A. Fernandez, Y. Hirano, M. Mauro, A. Lai, C. Ayuso, T. Haraguchi, Y. Hiraoka, F. Piano, P. Askjaer, Identification of conserved MEL-28/ELYS domains with essential roles in nuclear assembly and chromosome segregation. *PLOS Genet.* **12**, e1006131 (2016).

60. B. Vollmer, A. Schooley, R. Sachdev, N. Eisenhardt, A. M. Schneider, C. Sieverding, J. Madlung, U. Gerken, B. Macek, W. Antonin, Dimerization and direct membrane interaction of Nup53 contribute to nuclear pore complex assembly. *EMBO J.* **31**, 4072–4084 (2012).

61. S. Otsuka, J. Ellenberg, Mechanisms of nuclear pore complex assembly–two different ways of building one molecular machine. *FEBS Lett.* **592**, 475–488 (2018).

62. S. Otsuka, J. O. B. Tempkin, W. Zhang, A. Z. Politi, A. Rybina, M. J. Hossain, M. Kueblbeck, A. Callegari, B. Koch, N. R. Morero, A. Sali, J. Ellenberg, A quantitative map of nuclear pore assembly reveals two distinct mechanisms. *Nature* **613**, 575–581 (2023).

63. M. E. Hase, V. C. Cordes, Direct interaction with nup153 mediates binding of Tpr to the periphery of the nuclear pore complex. *Mol. Biol. Cell* **14**, 1923–1940 (2003).

64. S. G. Regmi, H. Lee, R. Kaufhold, B. Fichtman, S. Chen, V. Aksenova, E. Turcotte, A. Harel, A. Arnaoutov, M. Dasso, The nuclear pore complex consists of two independent scaffolds. bioRxiv 2020.11.13.381947; https://doi.org/10.1101/2020.11.13.381947 (2020).

65. N. Belgareh, G. Rabut, S. W. Baï, M. van Overbeek, J. Beaudouin, N. Daigle, O. V. Zatsepina, F. Pasteau, V. Labas, M. Fromont-Racine, J. Ellenberg, V. Doye, An evolutionarily conserved NPC subcomplex, which redistributes in part to kinetochores in mammalian cells. *J. Cell Biol.* **154**, 1147–1160 (2001).

66. E. Ródenas, C. González-Aguilera, C. Ayuso, P. Askjaer, Dissection of the NUP107 nuclear pore subcomplex reveals a novel interaction with spindle assembly checkpoint protein MAD1 in *Caenorhabditis elegans*. *Mol. Biol. Cell* **23**, 930–944 (2012).

67. M. Zuccolo, A. Alves, V. Galy, S. Bolhy, E. Formstecher, V. Racine, J. B. Sibarita, T. Fukagawa, R. Shiekhattar, T. Yen, V. Doye, The human Nup107-160 nuclear pore subcomplex contributes to proper kinetochore functions. *EMBO J.* **26**, 1853–1864 (2007).

68. P. Bensidoun, T. Reiter, B. Montpetit, D. Zenklusen, M. Oeffinger, Nuclear mRNA metabolism drives selective basket assembly on a subset of nuclear pore complexes in budding yeast. *Mol. Cell* **82**, 3856–3871.e6 (2022).

69. D. Kachaner, D. Garrido, H. Mehsen, K. Normandin, H. Lavoie, V. Archambault, Coupling of Polo kinase activation to nuclear localization by a bifunctional NLS is required during mitotic entry. *Nat. Commun.* **8**, 1701 (2017).

70. L. Pintard, V. Archambault, A unified view of spatio-temporal control of mitotic entry: Polo kinase as the key. *Open Biol.* **8**, 180114 (2018).

71. T. Stuwe, C. J. Bley, K. Thierbach, S. Petrovic, S. Schilbach, D. J. Mayo, T. Perriches, E. J. Rundlet, Y. E. Jeon, L. N. Collins, F. M. Huber, D. H. Lin, M. Paduch, A. Koide, V. Lu, J. Fischer, E. Hurt, S. Koide, A. A. Kossiakoff, A. Hoelz, Architecture of the fungal nuclear pore inner ring complex. *Science* **350**, 56–64 (2015).

72. J. Mansfeld, S. Güttinger, L. A. Hawryluk-Gara, N. Panté, M. Mall, V. Galy, U. Haselmann, P. Mühlhäusser, R. W. Wozniak, I. W. Mattaj, U. Kutay, W. Antonin, The conserved transmembrane nucleoporin NDC1 is required for nuclear pore complex assembly in vertebrate cells. *Mol. Cell* **22**, 93–103 (2006).

73. K. Zhu, Z. Shan, L. Zhang, W. Wen, Phospho-pon binding-mediated fine-tuning of Plk1 activity. *Structure* **24**, 1110–1119 (2016).

74. P. Singh, M. E. Pesenti, S. Maffini, S. Carmignani, M. Hedtfeld, A. Petrovic, A. Srinivasamani, T. Bange, A. Musacchio, BUB1 and CENP-U, Primed by CDK1, are the main PLK1 kinetochore receptors in mitosis. *Mol. Cell* **81**, 67–87.e9 (2021).

75. B. J. Blus, J. Koh, A. Krolak, H. S. Seo, E. Coutavas, G. Blobel, Allosteric modulation of nucleoporin assemblies by intrinsically disordered regions. *Sci. Adv.* **5**, eaax1836 (2019).

76. S. Brenner, The genetics of *Caenorhabditis elegans*. *Genetics* **77**, 71–94 (1974).

77. R. S. Kamath, M. Martinez-Campos, P. Zipperlen, A. G. Fraser, J. Ahringer, Effectiveness of specific RNA-mediated interference through ingested double-stranded RNA in *Caenorhabditis elegans*. *Genome Biol.* **2**, RESEARCH0002 (2001).
78. A. E. Friedland, Y. B. Tzur, K. M. Esvelt, M. P. Colaiácovo, G. M. Church, J. A. Calarco, Heritable genome editing in *C. elegans* via a CRISPR-Cas9 system. *Nat. Methods* **10**, 741–743 (2013).
79. J. A. Arribere, R. T. Bell, B. X. Fu, K. L. Artiles, P. S. Hartman, A. Z. Fire, Efficient marker-free recovery of custom genetic modifications with CRISPR/Cas9 in *Caenorhabditis elegans*. *Genetics* **198**, 837–846 (2014).
80. M. L. Schwartz, E. M. Jorgensen, SapTrap, a toolkit for high-throughput CRISPR/Cas9 gene modification in *Caenorhabditis elegans*. *Genetics* **202**, 1277–1288 (2016).
81. C. Muñoz-Jiménez, C. Ayuso, A. Dobrzynska, A. Torres-Mendéz, P. C. Ruiz, P. Askjaer, An efficient FLP-based toolkit for spatiotemporal control of gene expression in *Caenorhabditis elegans*. *Genetics* **206**, 1763–1778 (2017).
82. J. Vicencio, C. Martínez-Fernández, X. Serrat, J. Cerón, Efficient generation of endogenous fluorescent reporters by nested CRISPR in *Caenorhabditis elegans*. *Genetics* **211**, 1143–1154 (2019).
83. G. A. Dokshin, K. S. Ghanta, K. M. Piscopo, C. C. Mello, Robust genome editing with short single-stranded and long, partially single-stranded DNA donors in *Caenorhabditis elegans*. *Genetics* **210**, 781–787 (2018).
84. T. Stiernagle, *Maintenance of C. elegans*. (Oxford Univ. Press, 2006), pp. 1–11.
85. E. Zanin, J. Dumont, R. Gassmann, I. Cheeseman, P. Maddox, S. Bahmanyar, A. Carvalho, S. Niessen, J. R. Yates, K. Oegema, A. Desai, Affinity purification of protein complexes in *C. elegans*. *Methods Cell Biol.* **106**, 289–322 (2011).
86. F. Meier, S. Beck, N. Grassl, M. Lubeck, M. A. Park, O. Raether, M. Mann, Parallel accumulation-serial fragmentation (PASEF): Multiplying sequencing speed and sensitivity by synchronized scans in a trapped ion mobility device. *J. Proteome Res.* **14**, 5378–5387 (2015).
87. N. Joly, E. Beaumale, L. Van Hove, L. Martino, L. Pintard, Phosphorylation of the microtubule-severing AAA^+ enzyme Katanin regulates *C. elegans* embryo development. *J. Cell Biol.* **219**, e201912037 (2020).
88. S. Berg, D. Kutra, T. Kroeger, C. N. Straehle, B. X. Kausler, C. Haubold, M. Schiegg, J. Ales, T. Beier, M. Rudy, K. Eren, J. I. Cervantes, B. Xu, F. Beuttenmueller, A. Wolny, C. Zhang, U. Koethe, F. A. Hamprecht, A. Kreshuk, ilastik: Interactive machine learning for (bio)image analysis. *Nat. Methods* **16**, 1226–1232 (2019).
89. E. Voronina, G. Seydoux, The *C. elegans* homolog of nucleoporin Nup98 is required for the integrity and function of germline P granules. *Development* **137**, 1441–1450 (2010).

90. L. Thomas, B. Taleb Ismail, P. Askjaer, G. Seydoux, Nucleoporin foci are stress-sensitive condensates dispensable for *C. elegans* nuclear pore assembly. *EMBO J.*, e112987 (2023).
91. M. Sarov, J. I. Murray, K. Schanze, A. Pozniakovski, W. Niu, K. Angermann, S. Hasse, M. Rupprecht, E. Vinis, M. Tinney, E. Preston, A. Zinke, S. Enst, T. Teichgraber, J. Janette, K. Reis, S. Janosch, S. Schloissnig, R. K. Ejsmont, C. Slightam, X. Xu, S. K. Kim, V. Reinke, A. F. Stewart, M. Snyder, R. H. Waterston, A. A. Hyman, A genome-scale resource for in vivo tag-based protein function exploration in *C. elegans*. *Cell* **150**, 855–866 (2012).
92. N. Hattersley, D. Cheerambathur, M. Moyle, M. Stefanutti, A. Richardson, K. Y. Lee, J. Dumont, K. Oegema, A. Desai, A nucleoporin docks protein phosphatase 1 to direct meiotic chromosome segregation and nuclear assembly. *Dev. Cell* **38**, 463–477 (2016).
93. R. S. Kamath, A. G. Fraser, Y. Dong, G. Poulin, R. Durbin, M. Gotta, A. Kanapin, N. Le Bot, S. Moreno, M. Sohrmann, D. P. Welchman, P. Zipperlen, J. Ahringer, Systematic functional analysis of the *Caenorhabditis elegans* genome using RNAi. *Nature* **421**, 231–237 (2003).
94. M. Kumar, S. Michael, J. Alvarado-Valverde, B. Mészáros, H. Sámano-Sánchez, A. Zeke, L. Dobson, T. Lazar, M. Örd, A. Nagpal, N. Farahi, M. Käser, R. Kraleti, N. E. Davey, R. Pancsa, L. B. Chemes, T. J. Gibson, The eukaryotic linear motif resource: 2022 release. *Nucleic Acids Res.* **50**, D497–D508 (2022).
95. A. M. Waterhouse, J. B. Procter, D. M. Martin, M. Clamp, G. J. Barton, Jalview version 2—A multiple sequence alignment editor and analysis workbench. *Bioinformatics* **25**, 1189–1191 (2009).
96. C. A. Schneider, W. S. Rasband, K. W. Eliceiri, NIH Image to ImageJ: 25 years of image analysis. *Nat. Methods* **9**, 671–675 (2012).
